# Supplementary material for: Impact of evidence-based guidelines on healthcare utilisation and costs for disc related sciatica in the Netherlands: a population-based, cross-sectional study
Source: BMJ Open. 2024 Mar 11;14(3):e078459. doi: 10.1136/bmjopen-2023-078459 (PMC10936503; doi:10.1136/bmjopen-2023-078459)
Supplement: Supplementary data [file bmjopen-2023-078459supp001.pdf]

APPENDIX

Table S1. Used diagnosis treatment codes

**Diagnosis treatment codes**	**Description**
Included	
0330-12-00-1203	Pseudo radicular syndrome
0330-12-00-1204	Radicular syndrome
0330-12-00-1211<br>0305-13-00-1350	Spinal stenosis
0305-13-00-1360	Hernia Nuclei Pulposis
0305-13-00-1370	Spondylolisthesis
0308-02-25-2501<br>0308-02-25-2550<br>0308-02-25-2565<br>0308-02-25-2555<br>0308-02-25-2505<br>0308-02-25-2510<br>0308-02-25-2515	Degenerative disc disease
0330-12-00-1231<br>0305-13-00-1330<br>0305-13-00-1340	Low backpain
Excluded	
0330-12-00-1201<br>0330-12-00-1202<br>0330-05-00-0543<br>0305-12-00-1202<br>0305-12-00-1220<br>0305-12-00-1240<br>0308-02-25-2525<br>0308-02-25-2527<br>0308-02-25-2530<br>0308-02-25-2535	Cervical diagnosis codes
0330-02-00-0232<br>0330-02-00-0233<br>0330-02-00-0221<br>0330-02-00-0222<br>0330-02-00-0223<br>0308-02-21-2105<br>0308-02-21-2110	Tumors, malignancies of the spine

0308-02-21-2115	
0308-02-24-2405<br>0308-02-24-2411<br>0305-13-00-1301<br>0305-13-00-1302	Infectious spine disease
0305-13-00-1396<br>0308-02-23-2305<br>0308-02-23-2311	Spine trauma, fractures
0305-13-00-1397<br>0305-13-00-1380<br>0305-13-00-1381<br>0305-13-00-1383<br>0305-13-00-1391	Congenital disc disease, kyphosis, scoliosis

Table S2. Used surgical procedure codes

Care product code	Description
Included	
38437<br>38444<br>38438<br>38467	Discectomy
30327<br>30329	Laminectomy
38458<br>38459<br>38460<br>38464<br>38468<br>38469	Fusion

Table S3. Inclusion criteria for the LDH subgroups

**Lumbar disc herniation diagnosis group**	
Included diagnosis codes	
0305-13-00-1360	Hernia Nuclei Pulposis
0330-12-00-1203	Radicular syndrome
Excluded diagnosis codes	
0330-12-00-1201<br>0330-12-00-1202<br>0330-05-00-0543	Cervical diagnosis codes

0305-12-00-1202<br>0305-12-00-1220<br>0305-12-00-1240<br>0308-02-25-2525<br>0308-02-25-2527<br>0308-02-25-2530<br>0308-02-25-2535	
0330-02-00-0232<br>0330-02-00-0233<br>0330-02-00-0221<br>0330-02-00-0222<br>0330-02-00-0223<br>0308-02-21-2105<br>0308-02-21-2110<br>0308-02-21-2115	Tumors, malignancies of the spine
0308-02-24-2405<br>0308-02-24-2411<br>0305-13-00-1301<br>0305-13-00-1302	Infectious spine disease
0305-13-00-1396<br>0308-02-23-2305<br>0308-02-23-2311	Spine trauma, fractures
0305-13-00-1397<br>0305-13-00-1380<br>0305-13-00-1381<br>0305-13-00-1383<br>0305-13-00-1391	Congenital disc disease, kyphosis, scoliosis
**Included surgical procedure codes**	
38437<br>38444<br>38438<br>38467	Discectomy
30327<br>30329	Laminectomy
38458<br>38459<br>38460<br>38464<br>38468<br>38469	Fusion

**Discectomy group**

Included diagnosis codes	
0330-12-00-1203	Radicular syndrome
0330-12-00-1204	Pseudo radicular syndrome
0330-12-00-1211<br>0305-13-00-1350	Spinal stenosis
0305-13-00-1360	Hernia Nuclei Pulposis
0305-13-00-1370	Spondylolisthesis
0308-02-25-2501<br>0308-02-25-2550<br>0308-02-25-2565<br>0308-02-25-2555<br>0308-02-25-2505<br>0308-02-25-2510<br>0308-02-25-2515	Degenerative disc disease
0330-12-00-1231<br>0305-13-00-1330<br>0305-13-00-1340	Low backpain
Excluded diagnosis codes	
0330-12-00-1201<br>0330-12-00-1202<br>0330-05-00-0543<br>0305-12-00-1202<br>0305-12-00-1220<br>0305-12-00-1240<br>0308-02-25-2525<br>0308-02-25-2527<br>0308-02-25-2530<br>0308-02-25-2535	Cervical diagnosis codes
0330-02-00-0232<br>0330-02-00-0233<br>0330-02-00-0221<br>0330-02-00-0222<br>0330-02-00-0223<br>0308-02-21-2105<br>0308-02-21-2110<br>0308-02-21-2115	Tumors, malignancies of the spine
0308-02-24-2405<br>0308-02-24-2411<br>0305-13-00-1301<br>0305-13-00-1302	Infectious spine disease
0305-13-00-1396<br>0308-02-23-2305	Spine trauma, fractures

0308-02-23-2311	
0305-13-00-1397<br>0305-13-00-1380<br>0305-13-00-1381<br>0305-13-00-1383<br>0305-13-00-1391	Congenital disc disease, kyphosis, scoliosis
**Included surgical procedure codes**	
38437<br>38444<br>38438<br>38467	Discectomy

**Age (<56 years) group**	
Included diagnosis codes	
0330-12-00-1203	Pseudo radicular syndrome
0330-12-00-1204	Radicular syndrome
0330-12-00-1211<br>0305-13-00-1350	Spinal stenosis
0305-13-00-1360	Hernia Nuclei Pulposis
0305-13-00-1370	Spondylolisthesis
0308-02-25-2501<br>0308-02-25-2550<br>0308-02-25-2565<br>0308-02-25-2555<br>0308-02-25-2505<br>0308-02-25-2510<br>0308-02-25-2515	Degenerative disc disease
0330-12-00-1231<br>0305-13-00-1330<br>0305-13-00-1340	Low backpain
Excluded diagnosis codes	
0330-12-00-1201<br>0330-12-00-1202<br>0330-05-00-0543<br>0305-12-00-1202<br>0305-12-00-1220<br>0305-12-00-1240<br>0308-02-25-2525<br>0308-02-25-2527<br>0308-02-25-2530<br>0308-02-25-2535	Cervical diagnosis codes

0330-02-00-0232<br>0330-02-00-0233<br>0330-02-00-0221<br>0330-02-00-0222<br>0330-02-00-0223<br>0308-02-21-2105<br>0308-02-21-2110<br>0308-02-21-2115	Tumors, malignancies of the spine
0308-02-24-2405<br>0308-02-24-2411<br>0305-13-00-1301<br>0305-13-00-1302	Infectious spine disease
0305-13-00-1396<br>0308-02-23-2305<br>0308-02-23-2311	Spine trauma, fractures
0305-13-00-1397<br>0305-13-00-1380<br>0305-13-00-1381<br>0305-13-00-1383<br>0305-13-00-1391	Congenital disc disease, kyphosis, scoliosis
**Included surgical procedure codes**	
38437<br>38444<br>38438<br>38467	Discectomy
30327<br>30329	Laminectomy
38458<br>38459<br>38460<br>38464<br>38468<br>38469	Fusion

Table S4. Full table of surgical trends between 2007 and 2020 for hospital visitors with a likely diagnosis of lumbar disc herniation

	2007	2008	2009	2010	2011	2012	2013	2014	2015	2016	2017	2018	2019	2020
**Patients with a registered diagnosis of LDH[a]**														
Number of procedures	8009	8638	9048	9046	8708	8077	7987	8085	7183	6372	7290	6917	6983	5693
Per 10,000 inhabitants	6.3	6.7	7.0	6.9	6.6	6.1	6.0	6.0	5.3	4.7	5.3	5.0	5.0	4.0
Per 10,000 hospital visitors	1441	1500	1489	1425	1339	1361	1312	1317	1196	1024	1076	998	1012	974
Male	4278	4675	4822	4827	4645	4230	4177	4194	3802	3156	3737	3460	3567	2845
Female	3731	3963	4226	4219	4063	3847	3810	3891	3381	3216	3553	3457	3416	2848
Neurosurgery	6941	6905	7250	7629	7431	7004	6928	6982	6411	5935	6381	6286	6208	5211
Orthopedic surgery	1282	2023	2078	1735	1602	1364	1383	1422	1063	652	1216	932	1088	773
Teaching hospitals	4463	4279	4389	4074	3757	3698	4177	4281	3598	3542	3607	3394	3277	2634
General hospitals	3150	3059	3193	3071	3127	2550	1769	1801	1913	1770	1732	1794	1640	1187
Private clinics	75	950	1132	1565	1509	1554	1775	1734	1421	840	1698	1511	1888	1741
University hospitals	321	350	334	336	315	275	266	269	251	220	253	218	178	131
**Patients who had a discectomy**														
Number of procedures	7586	8673	9204	9403	9172	8561	8443	8425	6872	5931	6828	6522	6585	5344
Per 10,000 inhabitants	5.9	6.7	7.1	7.2	7.0	6.5	6.3	6.3	5.1	4.4	5.0	4.7	4.7	3.8
Per 10,000 hospital visitors	656	702	719	687	658	689	652	625	524	436	472	447	442	427
Male	4084	4714	4898	5021	4944	4504	4438	4379	3676	2951	3489	3259	3348	2645
Female	3502	3959	4306	4382	4228	4057	4005	4046	3196	2980	3339	3263	3237	2699
Neurosurgery	6727	6973	7380	7909	7832	7507	7463	7445	6271	5605	6153	6079	5992	5015
Orthopedic surgery	1083	2040	2167	1888	1736	1445	1403	1400	929	593	1062	806	1023	701
Teaching hospitals	4261	4263	4493	4255	3939	3967	4482	4508	3634	3530	3634	3392	3276	2616
General hospitals	2963	3079	3210	3151	3353	2711	1832	1851	1683	1407	1447	1616	1510	1055
Private clinics	8	960	1152	1634	1561	1589	1822	1775	1289	750	1495	1305	1604	1557
University hospitals	354	371	349	363	319	294	307	291	266	244	252	209	195	116
**Patients age < 56 years**														
Number of procedures	7192	8052	8363	8683	8383	7708	7414	7742	6827	6052	6691	6243	6484	5184
Per 10,000 inhabitants	8.6	9.6	10.0	10.4	10.0	9.2	8.9	9.3	8.3	7.3	8.1	7.6	7.8	6.2
Per 10,000 hospital visitors	1103	1187	1204	1181	1139	1200	1139	1183	1107	971	1028	969	1016	977
Male	3881	4386	4489	4638	4486	4060	3876	4011	3583	3019	3426	3105	3274	2540
Female	3311	3666	3874	4045	3897	3648	3538	3731	3244	3033	3265	3138	3210	2644
Neurosurgery	6198	6376	6632	7152	7054	6619	6371	6633	6085	5575	5807	5625	5694	4732
Orthopedic surgery	1230	2010	2059	1927	1711	1416	1425	1509	1093	753	1242	922	1158	754
Teaching hospitals	4026	3962	4098	3989	3651	3589	3868	4091	3434	3435	3362	3141	3150	2401
General hospitals	2703	2786	2846	2802	2927	2305	1589	1672	1759	1536	1538	1521	1443	1077
Private clinics	58	864	994	1467	1415	1404	1599	1582	1290	758	1473	1304	1625	1509
University hospitals	405	440	425	425	390	410	358	397	344	323	318	277	266	197

Table S5. Trends between 2007 and 2020 for hospital visitors with a likely diagnosis of lumbar disc herniation

	2007	2008	2009	2010	2011	2012	2013	2014	2015	2016	2017	2018	2019	2020
**Patients with a registered diagnosis of LDH[a]**														
Hospital visitors	55581	57597	60777	63493	65023	59365	60883	61396	60040	62217	67771	69296	68997	58442
Per 10,000 inhabitants	43	45	47	49	49	45	46	46	45	46	50	50	50	42
Male	28528	29674	31064	32271	32624	29446	29838	30190	29260	30104	32362	32836	32934	27733
Female	27053	27923	29713	31222	32399	29919	31045	31206	30780	32113	35409	36460	36063	30709
Neurology	48447	50803	53529	56499	57929	52870	54380	54382	53327	56397	60410	62255	62047	52314
Neurosurgery	8643	8409	7560	7970	7859	7522	9099	9644	10240	10487	12084	12444	12145	10648
Orthopedic surgery	7490	7944	8678	8437	8764	7435	6605	6954	6071	5097	6595	5840	5976	4843
Referred by neurologist to surgical department	7155	7526	7005	7506	7346	6896	7541	7790	7991	8180	9521	9457	9398	7838
Teaching hospitals	23509	23736	25298	25115	25964	24972	30872	30979	30855	31844	33391	34414	33453	28581
General hospitals	27714	26461	27189	28910	29079	24980	21083	22322	22026	22598	22525	22241	22224	18534
Private clinics	1774	4806	5797	6912	7461	7005	6518	5547	4963	5334	9394	10315	11097	9301
University hospitals	2584	2594	2493	2556	2519	2408	2410	2548	2196	2441	2461	2326	2223	2026
Mean per hospital														
Teaching hospitals	871	913	973	966	999	892	1065	1192	1234	1274	1336	1377	1338	1143
General hospitals	462	448	486	535	539	480	449	507	551	595	644	635	635	545
Private clinics	197	267	290	288	298	280	251	264	216	254	447	491	555	443
University hospitals	323	324	312	320	315	301	301	319	275	305	308	291	278	253
**Patients age < 56 years**														
Hospital visitors	65185	67861	69449	73503	73569	64257	65093	65463	61695	62308	65056	64399	63808	53077
Per 10,000 inhabitants	78	81	83	88	88	77	78	79	75	75	79	78	77	64
Male	32213	33336	34010	35468	35050	30228	30358	30501	28412	28623	29788	29004	28938	23817
Female	32972	34525	35439	38035	38519	34029	34735	34962	33283	33685	35268	35395	34870	29260
Neurology	48448	51585	54337	57659	57969	50346	50583	50188	47716	48789	50783	50658	11571	40686
Neurosurgery	8902	8747	7131	7665	7615	7342	9499	10158	10629	10300	11270	11415	49746	10581
Orthopedic surgery	15865	16559	16607	17370	17256	14589	13514	14121	11813	11584	12328	11343	11537	9144
Referred by neurologist to surgical department	5164	5562	5205	5574	5507	5021	5410	5488	5414	5281	6071	5928	5763	4656
Teaching hospitals	27871	28569	29173	30248	30182	27595	33030	32593	31141	31510	32770	32911	31988	25935
General	31533	30973	31641	33444	33092	26749	21881	22868	21473	21901	21370	20161	19668	15801
University hospitals	3969	4037	3655	3541	3437	3224	3153	3200	2873	3218	2897	2548	2480	2101
Private clinics	1812	4282	4980	6270	6858	6689	7029	6802	6208	5679	8019	8779	9672	9240
Mean per hospital														
Teaching hospital	214	284	311	338	337	317	332	372	348	348	413	425	467	422
General	513	525	565	627	620	527	482	523	533	574	628	589	586	491
University hospital	1072	1106	1133	1051	1020	884	1070	1226	1300	1314	1262	1225	1347	1006
Private clinics	496	505	457	443	430	403	394	400	359	402	362	319	310	263

[a] Lumbar Disc Herniation

Table S6. Full table of time between diagnosis, first hospital-visit and surgery between 2012 and 2020 (Nivel and NZA database)

	2012 (n=697)	2013 (n=958)	2014 (n=1174)	2015 (n=986)	2016 (n=804)	2017 (n=947)	2018 (n=902)	2019 (n=1031)	2020 (n=744)
Days between first GP[b] visit and first hospital visit (average)	158	155	163	179	180	174	184	169	193
**First GP visit to surgery**									
Days between first GP visit and surgery, median (25th-75th)	154 (80 – 361)	154 (77 – 328)	166 (87 – 351)	183 (91 – 361)	174 (92 – 371)	187 (103 – 354)	202 (104 – 386)	196 (104 – 383)	239 (137 – 451)
Days between first GP visit and surgery, 5th-95th	15 - 695	22 - 756	29 - 795	34 - 803	26 - 822	35 - 824	35 - 857	36 - 803	43 - 840
Days between first GP visit and surgery for patients <56 years, median (25th-75th)	137 (74-332)	147 (71-310)	142 (79-280)	167 (82-341)	161 (86-337)	160 (85-301)	172 (91-346)	183 (91-347)	230 (111-415)
**Time from first hospital visit to surgery**									
Days between first hospital visit and surgery, median (25th-75th)	51 (25-91)	51 (25-91)	56 (26-99)	62 (32-106)	59 (29-96)	70 (35-118)	67 (35-119)	70 (35-125)	80 (40-141)
Days between first hospital visit and surgery, 5th-95th	2 - 223	6 - 207	7 - 223	7 - 201	8 - 212	8 - 223	7 - 223	7 - 239	8 – 264
Days between first hospital visit and surgery for patients <56 years, median (25th-75th)	40 (21-78)	45 (21-81)	45 (21-81)	48 (23-86)	46 (22-78)	63 (31-105)	56 (23-108)	59 (28-110)	63 (31-118)
**Following the guideline?**									
Patients that had surgery within 2 days from diagnosis (GP) (%)	0.7	0.8	0.5	0.5	0.5	0.4	0.3	0.8	0.4
Patients that had surgery between 2 days and 12 weeks from diagnosis (GP) (%)	26.2	26.3	23.2	22.1	21.8	18.3	17.3	17.6	12.4
Patients that had surgery after 12 weeks and before 20 weeks from diagnosis (GP) (%)	20.2	18.6	19.7	16.3	19.4	17.3	17.3	15.7	13.0
Patients that had surgery after 20 weeks from diagnosis (GP) (%)	52.8	54.3	56.4	61.1	58.3	63.9	65.0	65.9	74.2
Patients <56 years that had surgery within 2 days from diagnosis (GP) (%)	1.4	0.8	0.3	0.7	0.3	0.5	0.3	1.1	0.0
Patients <56 years that had surgery between 2 days and 12 weeks from diagnosis (GP) (%)	30.7	28.7	26.8	25.5	24.6	24.3	20.3	20.0	17.2
Patients <age 56 years that had surgery after 12 weeks and before 20 weeks from diagnosis (GP) (%)	18.9	18.8	22.0	15.1	20.3	17.4	19.0	17.2	14.3
Patients <age 56 years that had surgery after 20 weeks from diagnosis (GP) (%)	49.0	51.7	50.8	58.7	54.8	57.7	60.4	61.6	68.5
Waiting time for surgery (days)	31	29	32	34	36	39	38	43	44

[a] number of surgical treated patients that could be linked between the NIVEL and NZA database [b] General Practitioner
