# Supplementary material for: Impact of evidence-based guidelines on healthcare utilisation and costs for disc related sciatica in the Netherlands: a population-based, cross-sectional study
Source: BMJ Open. 2024 Mar 11;14(3):e078459. doi: 10.1136/bmjopen-2023-078459 (PMC10936503; doi:10.1136/bmjopen-2023-078459)
Supplement: Author's manuscript [file bmjopen-2023-078459.draft_revisions.pdf]

BMJ Open

BMJ Open is committed to open peer review. As part of this commitment we make the peer review history of every article we publish publicly available.

When an article is published we post the peer reviewers' comments and the authors' responses online. We also post the versions of the paper that were used during peer review. These are the versions that the peer review comments apply to.

The versions of the paper that follow are the versions that were submitted during the peer review process. They are not the versions of record or the final published versions. They should not be cited or distributed as the published version of this manuscript.

BMJ Open is an open access journal and the full, final, typeset and author-corrected version of record of the manuscript is available on our site with no access controls, subscription charges or pay-per-view fees (http://bmjopen.bmj.com).

If you have any questions on BMJ Open's open peer review process please email info.bmjopen@bmj.com

BMJ Open

Trends in surgery rates for disc related sciatica in the Netherlands: a population-based study

Journal:	*BMJ Open*
Manuscript ID	bmjopen-2023-078459
Article Type:	Original research
Date Submitted by the Author:	02-Aug-2023
Complete List of Authors:	van Munster, Juliëtte; Leiden University, Department of Otorhinolaryngology and Head and Neck Surgery<br>Noordenbos, Mark; Dutch Healthcare Authority<br>Halperin, I.J.Y.; Leiden University, Department of Otorhinolaryngology and Head and Neck Surgery<br>van den Hout, Wilbert; Leiden University Medical Center, Medical Decision Making<br>van Benthem, Peter Paul; Leiden University Medical Center, Otorhinolaryngology - Head and Neck Surgery<br>Seinen, Ingrid; Dutch Healthcare Authority<br>Moojen, Wouter; Medical Center Haaglanden, Neurosurgery; HAGA hospital, Neurosurgery<br>Peul, Wilco; Leiden University Medical Center, Neurosurgery; Medical Center The Hague, Neurosurgery
Keywords:	Clinical Decision-Making, Protocols & guidelines < HEALTH SERVICES ADMINISTRATION & MANAGEMENT, Neuromuscular disease < NEUROLOGY

SCHOLARONE™
Manuscripts

I, the Submitting Author has the right to grant and does grant on behalf of all authors of the Work (as defined in the below author licence), an exclusive licence and/or a non-exclusive licence for contributions from authors who are: i) UK Crown employees; ii) where BMJ has agreed a CC-BY licence shall apply, and/or iii) in accordance with the terms applicable for US Federal Government officers or employees acting as part of their official duties; on a worldwide, perpetual, irrevocable, royalty-free basis to BMJ Publishing Group Ltd ("BMJ") its licensees and where the relevant Journal is co-owned by BMJ to the co-owners of the Journal, to publish the Work in this journal and any other BMJ products and to exploit all rights, as set out in our licence.

The Submitting Author accepts and understands that any supply made under these terms is made by BMJ to the Submitting Author unless you are acting as an employee on behalf of your employer or a postgraduate student of an affiliated institution which is paying any applicable article publishing charge ("APC") for Open Access articles. Where the Submitting Author wishes to make the Work available on an Open Access basis (and intends to pay the relevant APC), the terms of reuse of such Open Access shall be governed by a Creative Commons licence – details of these licences and which Creative Commons licence will apply to this Work are set out in our licence referred to above.

Other than as permitted in any relevant BMJ Author's Self Archiving Policies, I confirm this Work has not been accepted for publication elsewhere, is not being considered for publication elsewhere and does not duplicate material already published. I confirm all authors consent to publication of this Work and authorise the granting of this licence.

For peer review only

Title: Trends in surgery rates for disc related sciatica in the Netherlands: a population-based, cross-sectional study

Authors: Juliëtte J.C.M. van Munster[1,2] MD, Mark W. Noordenbos, PhD[3], I.J.Y. Halperin[1,2] MD, Wilbert B. van den Hout[4] PhD, Peter Paul G. van Benthem[2] MD, PhD, Ingrid Seinen, MSc[3], Wouter Moojen[1] MD, PhD, Wilco C. Peul[1,5] MD, PhD

[1] University Neurosurgical Centre Holland (UNCH), LUMC | HMC | HAGA, Leiden & The Hague, the Netherlands

[2] Department of Otorhinolaryngology and Head and Neck Surgery, Leiden University Medical Center, Leiden University, the Netherlands

[3] Dutch Healthcare Authority, Utrecht, the Netherlands.

[4] Department of Biomedical Data Sciences, Leiden University Medical Center, Leiden, the Netherlands

[5] Netherlands Organization for Health Research and Innovation (ZonMW)

Corresponding author:

Juliëtte van Munster, Dept. of Neurosurgery, Leiden University Medical Centre, P.O. Box 9600, 2300 RC Leiden, the Netherlands, Phone: +3171 526 5870, e-mail: j.j.c.m.van_munster@lumc.nl

Conflict of interest: Prof. dr. Peul: Boardmember ZonMW, former Chair Netherlands Choosing Wisely FMS committee

Prof. dr. van Benthem: Former president of the Federation Medical Specialists (FMS)

Dr. W. Moojen is a Board member of the Dutch Neurosurgical Society.

Supported by a grant from: the Netherlands Organization for Health Research and Development (Citrienfonds), grant number: 839205002. The funding organization had no role in the present study.

Ethical approval: our study was approved by the Medical Ethical Committee of Leiden and the Hague (metc-ldd@lumc.nl; N20.075)

For peer review only - http://bmjopen.bmj.com/site/about/guidelines.xhtml

Word count: 4194

Target journal: BMJ Evidence-Based Medicine

ABSTRACT

Objective: The aim of this study was to assess the impact of high-quality evidence supporting surgical treatment of lumbar disc herniation (LDH) on healthcare practice in the Netherlands by examining changes in healthcare utilization, including the timing of surgery, and healthcare costs for LDH patients.

Design: A retrospective, cross-sectional study was performed utilizing population-based, longitudinal data obtained from the Dutch Healthcare Authority (2007-2020) and NIVEL's primary care (2012-2020) administrative databases.

Setting: The study was conducted within the healthcare system of the Netherlands.

Participants: We included adults (>18 years) who visited a Dutch hospital or a General Practitioner for lumbar degenerative disc disease. LDH patients were identified based on registered codes, type of surgery (discectomy), and age (<56 years).

Main outcome measures: The primary outcome measure was the difference in the annual number of procedures following the publication of evidence-based guidelines in 2009 (comparing the periods 2007-2009 to 2017-2019). Secondary outcome measures focused on the timing of surgery and associated healthcare costs.

Results: Number of patients suffering from LDH increased from 55.581 to 68.997 (+24%) between 2007 and 2019. A decrease was observed in annual number of LDH procedures (-18%), in number of discectomies (-22%), and in number of procedures for patients age <56 years (-18%). This resulted in lower healthcare costs by €12 million annually. In 2012, 31% of all patients <56 years had surgery before 12 weeks from diagnosis at the GP, whereas 20% did in 2019.

For peer review only

For peer review only - http://bmjopen.bmj.com/site/about/guidelines.xhtml

Conclusions: The implementation of evidence-based guidelines for sciatica caused by LDH had a significant influence on healthcare practice in the Netherlands. The observed decrease in the number of procedures has been accompanied by a corresponding reduction in healthcare costs. These findings underscore the importance of adhering to evidence-based guidelines to optimize the management of LDH patients and prevent unnecessary interventions.

INTRODUCTION

Lumbosacral Radicular Syndrome (LRS), or sciatica due to Degenerative Disc Disease (DDD), is common in everyday healthcare practices with a substantial burden for healthcare and society[1]. LRS is mostly caused by a lumbar disc herniation (LDH). Intolerable pain and cauda equina syndrome are definite indications for surgery, but most patients suffering from sciatica recover without the need of surgery within three months from onset[2], after which period the natural course is less favorable with a longer wait-and-see strategy[3].

In 1999, in response to a provoking practice variation publication[4], the Health Council of the Netherlands published an evidence-based report on this topic and concluded that there was lack of evidence on effectiveness and timing of surgery, but also serious signs of overtreatment in the Netherlands compared to the United Kingdom (UK)[5]. In response, a Randomized Controlled Trial was designed and performed on this topic in the Netherlands (Sciatica trial; 2007)[6]. Comparing the Netherlands with UK guidelines in timing of discectomy, patients suffering from severe sciatica for 6 to 12 weeks were randomly assigned to a timing of early surgery or to prolonged conservative treatment up to 6 months with possible delayed surgery. Around the same time, the SPORT trial (Weinstein et al.; 2006) was performed in the United States of America (USA)[7]. Both trials showed no more differences in pain scores beyond one year, but with faster initial pain relief and better quality-adjusted life years after early surgery compared to conservative treatment[6 8]. As a result, early surgery was a cost-effective option that need not to be withheld for economic reasons, although it was associated with higher healthcare costs[9].

For peer review only - http://bmjopen.bmj.com/site/about/guidelines.xhtml

These trials led to the development of the first multidisciplinary, evidence-based guideline for LRS in the Netherlands in 2009[10] and several other countries[11 12]. An initial conservative treatment strategy is recommended in the first three months after onset of sciatica (in absence of progressive neurological deficit and cauda equina syndrome), whereas a shared decision-making treatment strategy between patient and physician is advised between three and six months. Guideline recommendations were incorporated in 'Wise Choices' in the Dutch version of the North American initiated Choosing Wisely campaign in 2014[13 14]. Also, these recommendations were integrated in the General Practitioner (GP) guideline in 2015[15].

It was decided by the original researchers in collaboration with the Federation Medical Specialists (FMS) and the Dutch Healthcare Authority that now is the right time to evaluate whether the evidence-based guideline recommendations changed healthcare utilization in the Netherlands between 2007 and 2020. We focused on number of procedures, timing of surgical treatment for LDH inflicted sciatica, and healthcare pathways. Secondary, we were interested in changes in societal and healthcare costs.

METHODS

A population-based, longitudinal, cross-sectional study was performed.

Data sources

Two databases were analyzed; firstly, a comprehensive nationwide, administrative healthcare database provided by the Dutch Healthcare Authority. This database contains detailed information on diagnosis by diagnosis-related groups, healthcare activities and fundamental patient characteristics (e.g. gender, year of birth) covering all patients treated in hospitals throughout the Netherlands[16]. Secondly, NIVEL's (Netherlands Institute for Health Services Research) Primary Care Database was analyzed. This database comprises routine healthcare data recorded between 2012 and 2020 and represents a 10% sample of all general practitioner (GP) practices in the country [17]. To ensure patient confidentiality, individuals in both databases were linked using a unique anonymized identifier, thereby preventing access to sensitive personal patient data by the researchers.

For peer review only - http://bmjopen.bmj.com/site/about/guidelines.xhtml

For peer review only

Population

This study focused on Dutch individuals aged 18 years and above who sought medical care for lumbar degenerative disc disease (DDD) at hospitals between 2007 and 2020. Patients' hospital data were collected on the diagnosis codes for lumbar DDD, and the corresponding surgical care products (discectomy, laminectomy, and instrumented spinal fusion; Table S1 and S2). To mitigate potential data incompleteness, multiple diagnosis codes were incorporated, as different healthcare providers may employ varying registration practices. Exclusion criteria included patients with cervical degenerative disc disease, spinal infection, traumatic or oncological fractures, spinal deformities, and those who had undergone previous back surgery within the past year. In the NIVEL database, adult patients diagnosed with lumbo-radicular syndrome (coded as L86) were included.

The objective of this analysis was to evaluate the impact of guideline implementation on patients with sciatica attributed to lumbar disc herniation (LDH). The authors hypothesized that these patients would possess a registered diagnosis of LDH, have undergone discectomy as a treatment modality, and belong to a younger age group (<56 years) in comparison to individuals with spinal stenosis—a degenerative condition commonly observed in the elderly.

For peer review only

Data collection

Firstly, annual number of hospital visits and procedures were collected. Furthermore, we collected patients' age and gender, hospital type (general hospital, (top clinical) teaching hospital, university hospital, or private clinic), attending medical specialist (neurology, neurosurgery, or orthopedic surgery), date of first hospital visit, date of surgery and information on hospital waiting time for hernia-related procedures (days between order request and day of procedure). Surgical patients were assigned to the hospital where the surgery was performed. Non-surgical patients were assigned to the last hospital they visited. From the NIVEL database, date of first visit to the GP was collected.

Analyses

Absolute numbers of diagnoses and procedures were calculated between 2007 and 2020. To provide a more accurate representation, surgical rates were adjusted based on the population of individuals aged 18 years and older residing in the Netherlands. Adjusted surgical rates were calculated per 10,000

For peer review only - http://bmjopen.bmj.com/site/about/guidelines.xhtml

inhabitants, thereby normalizing the data. Additionally, we calculated in-hospital surgical rates to assess the proportion of referrals with an indication for surgical intervention. Directly adjusted surgical rates per 10,000 inhabitants or 10,000 hospital visitors were calculated using the following formula:

$$\frac{Number\ of\ surgical\ procedures}{Number\ of\ inhabitants\ \geq 18\ years\ /\ hospital\ visitors} \times\ 10{,}000$$

Furthermore, the timing of surgical interventions was analyzed by calculating the median number of days between the initial visit to the general practitioner (GP) and the date of surgery. Lastly, we assessed the median number of days between first visit hospital consultation and date of surgical procedure.

Cost analysis

Differences in national healthcare and societal costs were calculated by comparing the annual number of LDH patients per 10,0000 over 2007-2009 to that number over pre-covid years 2017-2019. We updated our previous economic evaluation[9] to the population size and price level in 2022, using current surgical prices and updating other costs by the Dutch consumer price index[16 18]. Thus, the difference between a surgical and a conservative policy was estimated at €2596 in healthcare costs and €107 in societal costs per patient. With a 49% difference in surgery probability between both policies[9], these estimates translate to the slightly more than double €5298 in healthcare savings and €218 societal savings per prevented surgery. Societal savings are negligible, because the conservative policy has higher productivity costs.

Analytic approach

All analyses were conducted at the individual patient level. To assess differences in annual healthcare utilization, a comparison was made between the years preceding the publication of the guidelines (2007-2009) and the most recent years (2017-2019). Due to the limited usefulness of the data from the COVID-impacted year 2020, it was included in the database but not used for direct comparisons. However, the inclusion of 2020 was deemed important to demonstrate the potential impact of the pandemic on healthcare utilization. A larger decrease in procedures in 2020 might reflect undertreatment, which can

For peer review only

For peer review only - http://bmjopen.bmj.com/site/about/guidelines.xhtml

lead to chronic disability of patients waiting too long with persistent sciatica leading to chronic disease. We chose for a descriptive analysis, considering the anticipated statistical significance of even small differences given the large size of the database. All analyses were performed using R version 4.1.3[19].

RESULTS

Study population

During the period from 2007 to 2020, a total of 1.9 million patients sought hospital care for lumbar degenerative disc disease (DDD). Median age of these patients was 56 years and 54% was female (Table 1). Among these patients, 12% underwent surgical treatment (n=217.166). The most common surgical procedure was a discectomy (6%) or laminectomy (6%), while instrumented spinal fusion was less frequently utilized. In 53% of all cases, surgical patients were older than 56 years old and 51% was male. Teaching hospitals accounted for 52% of all procedures. Between 2012 and 2020, the NIVEL database, specifically the L86 code representing lumbo-radicular syndrome, matched with the hospital database, resulting in 52,057 patients being included in the analysis. Of these patients, 53% was female (compared to 54% in the hospital database), and the mean age was 56 years (compared to 52.4 in 2007 and 57.6 in 2020 in the hospital database). Moreover, among these patients, 16% (8,267 individuals) underwent surgery, showing a slightly higher surgical rate compared to the hospital database, which reported a 12% surgical rate.

For peer review only

Changes in healthcare utilization between 2007-2009 and 2017-2019

Absolute number of procedures for sciatica patients decreased between 18% and 22% over time (depending on the chosen definition), although an initial increase in number of procedures was observed in all three groups between 2007 and 2010 (Figure 1a, Table 2). Annual number of surgical procedures in private clinics increased from 719 in the years 2007-2009 to 1699 in the years 2017-2019 (Figure 1b, Table 2). Number of patients in teaching hospitals increased as well (+40%), whereas numbers of surgical procedures decreased in teaching hospitals (-22%). Number of visitors and procedures decreased in general hospitals (-18%; -45%) and university hospitals (-9%; -35%) in the period 2017-2019 compared to 2007-2009.

For peer review only - http://bmjopen.bmj.com/site/about/guidelines.xhtml

Changes in healthcare pathways between 2007 and 2019

The number of hospital visitors diagnosed with lumbar disc herniation (LDH) increased from 55,581 in 2007 to 68,997 in 2019, as shown in Figure 2a and detailed in Table S3. Concurrently, the absolute number of LDH patients referred by neurologists to surgical departments rose from 7,155 in 2007 to 9,398 in 2019, representing a 31% increase over the study period. However, the surgical rate per 10,000 hospital visitors declined from 1,441 per 10,000 in 2007 to 1,012 per 10,000 in 2019 (Figure 2a, Table 2). Notably, this decrease was more pronounced among neurosurgeons, dropping from 80% in 2007 to 51% in 2019, compared to orthopedic surgeons, whose surgical rate decreased from 11% in 2007 to 8% in 2019. In terms of referrals between hospitals for surgical and non-surgical LDH patients, there was no substantial change observed between 2007 and 2020 across different hospital types, as depicted in Figure 2b.

Timing of surgery between 2012 and 2020

The median duration in days between the initial diagnosis at the general practitioner (GP) and the date of surgery exhibited an increase from 154 days in 2012 to 196 days in 2019, as shown in Table 3. However, the time interval between the first GP visit and the first hospital visit, also known as the time to referral, remained stable (155 days). Moreover, days between first hospital visit and date of surgery increased from 71 days to 93 days (+22 days). Concurrently, the waiting time for hernia-related procedures in hospitals also increased, with an interval of 31 days in 2012 expanding to 43 days in 2019, reflecting a 12-day prolongation. Among patients below the age of 56 years, there was a decrease in the percentage of individuals who underwent surgery within 12 weeks from the time of diagnosis, declining from 31% in 2012 to 20% in 2019. Conversely, there was an increase in the proportion of patients who received surgery after 20 weeks from diagnosis, rising from 49% in 2012 to 62% in 2019. These findings highlight a shift towards longer waiting periods for surgical intervention in this patient group.

Cost analysis

Changes in healthcare utilization for sciatica due to LDH led to an estimated decrease of 1.5 per 10,000 inhabitants aged 18 years and older living in the

For peer review only - http://bmjopen.bmj.com/site/about/guidelines.xhtml

Netherlands. For 2022, the associated savings were estimated at a considerable 10 million euro on healthcare costs and only 0.5 million euro on societal costs.

DISCUSSION

Immediately following the publication of the Sciatica trial[6], an initial increase in surgical procedures for LDH patients was observed in the Netherlands. However, after 2010, a gradual decrease in the total numbers of discectomy occurred, resulting in substantial annual reductions in healthcare costs and in societal costs. Noteworthy changes in healthcare pathways revealed an increase in the number of referrals per surgeon, while the in-hospital surgical rate decreased. These findings suggest that both surgeons and patients are increasingly opting for an initial conservative treatment rather than early surgery. Regarding the timing of disc surgery for sciatica, there were notable changes between 2012 and 2019. The median time interval between the initial GP visit and the date of surgery increased, resulting in 82% of all patients undergoing surgery more than 12 weeks after disease onset in 2019. It is important to note that the recommended time window for surgery, as advised in the 2009 Lumbo-Radicular Syndrome (LRS) guideline, indicating surgery at least 12 weeks after disease onset for the expectant wait-and-see period, was already met in 2012. Additionally, hospital waiting times for surgery also increased between 2012 and 2019, which does not necessarily align with surgeon's treatment choices. Moreover, there was an increase in the percentage of patients who received surgery after more than 20 weeks.

In this study, we focused on recommendations from the SPORT- and Sciatica trial[6], but very recent research showed that 20 weeks after disease onset, outcomes are significantly worse in the even more prolonged conservative care group beyond the guideline time window compared to surgical treatment[3]. Therefore, a careful caution in patient guidance is warranted to prevent chronic disabling sciatica, due to irreversible damage to lumbosacral nerve roots by chronic compression[20]. As this Canadian study was published during Covid times and not yet implemented in Dutch evidence-based guidelines[3], it is not possible to do a valid investigation in the proportion of patients with a too late indication for surgery.

Our findings are important, because the Netherlands used to perform the second-most operations of the lumbar spine per 100,000 inhabitants compared to

For peer review only - http://bmjopen.bmj.com/site/about/guidelines.xhtml

other high-income countries[4]. The number of procedures for LDH, however, increased in the first years after publication of the Sciatica trial[6]. This increasing rate probably resulted from the published fact that study outcomes showed that surgery leads to faster pain relief compared to conservative treatment and was cost-effective compared to conservative care with possibly delayed surgery[9]. However, this might also be caused by the fact that data were incomplete for the years before 2011, meaning that the decrease in procedures that we observed is probably an underestimation of the true decrease in procedures. The decrease after 2010 might have been initiated by the publication of guideline recommendations in 2009, but also by the observation of high practice variation in surgical treatment for LDH in the Netherlands in 2010[21] leading to a task force of the medical specialist federation (FMS in collaboration with ZonMW) starting the Netherlands version of the international Choosing Wisely campaign. The active dissemination of the guideline, collaboration of FMS/ZonmW with insurance companies and Patient Federation in the development of wise choices led to awareness of the favorable natural course in most sciatica patients. Not unimportantly the growing attention for evidence-based surgery[22] within the medical specialist scientific societies, probably speeded up the implementation of the guideline in clinical practice[23].

Notably, a significant shift in decision-making was observed among neurosurgeons and orthopedic surgeons, who increasingly adhered to the guideline recommendations. In contrast, general practitioners (GPs) and neurologists showed a tendency to refer even more patients with lumbar disc herniation (LDH) than before. Furthermore, the time interval between the first GP visit and the first hospital visit remained unchanged between 2012 and 2019. This observation may indicate that patients have a preference for consulting a surgeon to discuss treatment options for LDH or that healthcare delivery for LDH patients is increasingly organized through multidisciplinary LDH teams, as described by Michael Porter[24]. Many hospitals in the Netherlands have implemented Integrated Practice Units, so called ‘Herniated Disc-Diagnosis-Treatment Streets’, where patients receive a joint appointment with both a neurologist and a surgeon, facilitating collaborative decision-making and comprehensive care.

In contrast to other hospital types, a tremendous increase in numbers of procedures for LDH patients was observed in private clinics. This might be attributed to a nationwide strategy aimed at redistributing and decentralizing high-volume, low-complexity diagnoses such as sciatica from university and large teaching hospitals to private clinics[25]. This approach helps to concentrate resources and expertise in the larger hospitals, which primarily handle more complex cases.

For peer review only

For peer review only - http://bmjopen.bmj.com/site/about/guidelines.xhtml

This results in a relief of pressure on the large hospitals, which was especially important during the COVID-19 pandemic: care could be maintained for these patients that required urgent or emergency neurosurgical care[26].

A strength of this study is the nationwide coverage of the database. Furthermore, LDH patients were identified by analyzing three patient identifiers (i.e. age, discectomy, and registered code for LDH) in order to validate the findings. The fact that academic physicians performed this analysis together with policy makers from the Dutch Healthcare Authority is another strength of this study and supports the independence of the study results.

It must be mentioned, however, that two of the current supervising investigators were the Sciatica Trial's health economist (WH) and principal investigator (WP), while the latter was also involved in the Guideline construction and leader of the Netherlands Choosing Wisely campaign on behalf of FMS and ZonMW. Although both researchers were initially kept out of the data analysis of the current study, some influence might be inevitable. Another limitation of this study is the fact that it is not possible to prove causation between guideline publication and changes in healthcare utilization. Measuring the true impact of the Sciatica trial was challenging due to the unavailability of high-quality data prior to 2007. The most recent available data on the total number of lumbar disc surgeries for sciatica in the Netherlands dates back to 1996, with 11,323 patients per year[5]. Similarly, in Finland the largest reduction in discectomies occured before 2007[27], probably caused by the starting awareness of ongoing studies in USA and Europe and the earlier landmark publication by Cherkin and Deyo warning against overtreatment in spine surgery[4].

CONCLUSION

Although causation cannot be proven, this study highlights the substantial impact of implementing high-quality randomized clinical trials and evidence-based guidelines on clinical practice. The findings suggest that the guideline recommendations for surgical treatment and timing of surgery for sciatica resulting from lumbar disc herniation (LDH) were effectively implemented in clinical practice in the Netherlands after 2007. This implementation led to a significant decrease in the number of discectomy procedures, with a longer timeframe between the initial visit to the general practitioner and surgery and subsequently a reduction in healthcare costs. To ensure the continued improvement of quality of care, it is essential to incorporate the findings of studies such as Bailey et al. into evidence-based guidelines, thereby preventing undertreatment of LDH patients in the future.

For peer review only

For peer review only - http://bmjopen.bmj.com/site/about/guidelines.xhtml

REFERENCES

For peer review only

1. Ravindra VM, Senglaub SS, Rattani A, et al. Degenerative Lumbar Spine Disease: Estimating Global Incidence and Worldwide Volume. *Global Spine J* 2018;8(8):784-94. doi: 10.1177/2192568218770769 [published Online First: 2018/12/19]
2. Hofstee DJ, Gijtenbeek JM, Hoogland PH, et al. Westeinde sciatica trial: randomized controlled study of bed rest and physiotherapy for acute sciatica. *J Neurosurg* 2002;96(1 Suppl):45-9. doi: 10.3171/spi.2002.96.1.0045 [published Online First: 2002/01/19]
3. Bailey CS, Rasoulinejad P, Taylor D, et al. Surgery versus Conservative Care for Persistent Sciatica Lasting 4 to 12 Months. *New England Journal of Medicine* 2020;382(12):1093-102. doi: 10.1056/NEJMoa1912658
4. Cherkin DC, Deyo RA, Loeser JD, et al. An international comparison of back surgery rates. *Spine (Phila Pa 1976)* 1994;19(11):1201-6. doi: 10.1097/00007632-199405310-00001 [published Online First: 1994/06/01]
5. Gezondheidsraad. Diagnostiek en behandeling van het radiculaire syndroom. In: Gezondheidsraad, ed. Den Haag, 1999.
6. Peul WC, van Houwelingen HC, van den Hout WB, et al. Surgery versus prolonged conservative treatment for sciatica. *N Engl J Med* 2007;356(22):2245-56. doi: 10.1056/NEJMoa064039 [published Online First: 2007/06/01]
7. Weinstein JN, Tosteson TD, Lurie JD, et al. Surgical vs nonoperative treatment for lumbar disk herniation: the Spine Patient Outcomes Research Trial (SPORT): a randomized trial. *Jama* 2006;296(20):2441-50. doi: 10.1001/jama.296.20.2441 [published Online First: 2006/11/23]
8. Weinstein JN, Tosteson TD, Lurie JD, et al. Surgical vs Nonoperative Treatment for Lumbar Disk HerniationThe Spine Patient Outcomes Research Trial (SPORT): A Randomized Trial. *JAMA* 2006;296(20):2441-50. doi: 10.1001/jama.296.20.2441
9. van den Hout WB, Peul WC, Koes BW, et al. Prolonged conservative care versus early surgery in patients with sciatica from lumbar disc herniation: cost utility analysis alongside a randomised controlled trial. *Bmj* 2008;336(7657):1351-4. doi: 10.1136/bmj.39583.709074.BE [published Online First: 2008/05/27]
10. CBO KvdG. Lumbosacraal Radiculair Syndroom 2008.
11. Krendl R. Lumbale Radikulopathie: Springer, 2014.
12. Diagnosis and treatment of lumbar disc herniation with radiculopathy. North American Spine Society; 2012.
13. Verstandige keuzes bij een lage rughernia.
14. Levinson W, Kallewaard M, Bhatia RS, et al. 'Choosing Wisely': a growing international campaign. *BMJ Qual Saf* 2015;24(2):167-74. doi: 10.1136/bmjqs-2014-003821 [published Online First: 2015/01/02]
15. Schaafstra AS, W.E.M.; Bons, S.C.S.; Borg, M.A.J.P.; Koes, B.W.; Ostelo, R.W.J.G.; Spijker-Huiges, A.; Burgers, J.S.; Bouma, M.; Verburg-Oorthuizen, A.F.E. Lumbosacraal radiculair syndroom. In: NHG-werkgroep, ed., 2015.
16. Statistics Netherlands Netherlands [Available from: https://www.cbs.nl/en-gb.
17. Netherlands Institute for Health Services Research [Available from: https://www.nivel.nl/en.
18. Hakkaart-van Roijen L, van der Linden N, Bouwmans C, et al. Kostenhandleiding: Methodologie van kostenonderzoek enreferentieprijzen voor economische evaluatiesin de gezondheidszorg: Institute for Medical Technology Assessment, Erasmus Universiteit Rotterdam, 2012.
19. program]. 4.1.3. version, 2022.
20. Nygaard OP, Romner B, Trumpy JH. Duration of symptoms as a predictor of outcome after lumbar disc surgery. *Acta Neurochir (Wien)* 1994;128(1-4):53-6. doi: 10.1007/bf01400653 [published Online First: 1994/01/01]
21. van Beek EL, K.; van Schooten, G.; Vlieger, E.J. Reduceren van praktijkvariatie: budgettaire effecten van scherpere indicatiestelling. In: Ministerie van Volksgezondheid WeS, ed., 2010.

For peer review only

For peer review only - http://bmjopen.bmj.com/site/about/guidelines.xhtml

22. McCulloch P, Altman DG, Campbell WB, et al. No surgical innovation without evaluation: the IDEAL recommendations. *Lancet* 2009;374(9695):1105-12. doi: 10.1016/s0140-6736(09)61116-8 [published Online First: 2009/09/29]
23. Lomas J. Words without action? The production, dissemination, and impact of consensus recommendations. *Annu Rev Public Health* 1991;12:41-65. doi: 10.1146/annurev.pu.12.050191.000353 [published Online First: 1991/01/01]
24. Porter ME, Teisberg EO. Redefining competition in health care. *Harv Bus Rev* 2004;82(6):64-76, 136. [published Online First: 2004/06/19]
25. Aandeel zelfstandige behandelcentra blijft toenemen 2018 [Available from: https://www.vektis.nl/actueel/aandeel-zelfstandige-behandelcentra-blijft-toenemen.
26. Ashkan K, Jung J, Velicu AM, et al. Neurosurgery and coronavirus: impact and challenges—lessons learnt from the first wave of a global pandemic. *Acta Neurochirurgica* 2021;163(2):317-29. doi: 10.1007/s00701-020-04652-8
27. Ponkilainen VT, Mäntymäki H, Huttunen TT, et al. Decreasing Incidence of Lumbar Discectomy Surgery in Finland in 1997-2018. *Spine (Phila Pa 1976)* 2021;46(6):383-90. doi: 10.1097/brs.0000000000003790 [published Online First: 2021/02/24]

For peer review only

Table 1. baseline characteristics of the total lumbar degenerative disc disease population between 2007 and 2020

Characteristic	Total number of lumbar DDD patients (2007-2020, n=1863627 (100%[a]))	Surgically treated patients (2007-2020, n=217166 (100%))
**Age group (n, %)**		
18-45 years	523431 (28)	58648 (27)
46-55 years	391292 (21)	42370 (20)
56 years and older	948904 (51)	116098 (53)
**Gender, (n, %)**		
Male	856732 (46)	110922 (51)
Female	1006895 (54)	106194 (49)
**Diagnosis, (n, %)**		
Lumbar disc herniation	870878 (47)	108036 (50)
Lumbar stenosis	317360 (17)	65977 (30)
Lumbar disc herniation and lumbar stenosis	46050 (2)	31045 (14)
Low back pain	587695 (32)	4899 (2)
Unknown	41644 (2)	7159 (3)
**Hospital type, (n, %)**		
General hospital	736250 (40)	63892 (29)
Teaching hospital	884234 (47)	113444 (52)
University hospital	94071 (5)	13627 (6)
Private clinic	149072 (8)	26153 (12)
**Department, (n, %)**		
Neurology department	1464011 (79)	142786 (66)
Neurosurgery department	303626 (16)	189418 (87)
Orthopedic surgery department	356674 (19)	39172 (18)

For peer review only

**Type of treatment[b], (n, %)**		
Discectomy	107549 (6)	107549 (50)
Laminectomy	109477 (6)	109477 (9)
Fusion	20449 (1)	20449 (50)
No surgical treatment	1626152 (88)	-
**Referred from another hospital (n=1777532[c]), (n, %)**		
Yes	85164 (5)	44793 (26)
No	1692368 (95)	126835 (74)

[a] due to rounding, the number of percentages might not add up to 100% [b] due to the fact that patients might receive two types of surgery, the percentages might not add up to 100%

[c] Patients who visited more than one hospital on the same day were not included in this group

For peer review only

Table 2. Surgical trends between 2007 and 2020 for hospital visitors with a likely diagnosis of lumbar disc herniation

For peer review only

For peer review only - http://bmjopen.bmj.com/site/about/guidelines.xhtml

	2007	2008	2009	2010	2011	2012	2013	2014	2015	2016	2017	2018	2019	2020
**Patients with a registered diagnosis of LDH[a]**														
Number of procedures	8009	8638	9048	9046	8708	8077	7987	8085	7183	6372	7290	6917	6983	5693
Per 10,000 inhabitants	6.3	6.7	7.0	6.9	6.6	6.1	6.0	6.0	5.3	4.7	5.3	5.0	5.0	4.0
Per 10,000 hospital visitors	1441	1500	1489	1425	1339	1361	1312	1317	1196	1024	1076	998	1012	974
Male	4278	4675	4822	4827	4645	4230	4177	4194	3802	3156	3737	3460	3567	2845
Female	3731	3963	4226	4219	4063	3847	3810	3891	3381	3216	3553	3457	3416	2848
Neurosurgery	6941	6905	7250	7629	7431	7004	6928	6982	6411	5935	6381	6286	6208	5211
Orthopedic surgery	1282	2023	2078	1735	1602	1364	1383	1422	1063	652	1216	932	1088	773
Teaching hospitals	4463	4279	4389	4074	3757	3698	4177	4281	3598	3542	3607	3394	3277	2634
General hospitals	3150	3059	3193	3071	3127	2550	1769	1801	1913	1770	1732	1794	1640	1187
Private clinics	75	950	1132	1565	1509	1554	1775	1734	1421	840	1698	1511	1888	1741
University hospitals	321	350	334	336	315	275	266	269	251	220	253	218	178	131
**Patients who had a discectomy**														
Number of procedures	7586	8673	9204	9403	9172	8561	8443	8425	6872	5931	6828	6522	6585	5344
Per 10,000 inhabitants	5.9	6.7	7.1	7.2	7.0	6.5	6.3	6.3	5.1	4.4	5.0	4.7	4.7	3.8
Per 10,000 hospital visitors	656	702	719	687	658	689	652	625	524	436	472	447	442	427
Male	4084	4714	4898	5021	4944	4504	4438	4379	3676	2951	3489	3259	3348	2645
Female	3502	3959	4306	4382	4228	4057	4005	4046	3196	2980	3339	3263	3237	2699
Neurosurgery	6727	6973	7380	7909	7832	7507	7463	7445	6271	5605	6153	6079	5992	5015
Orthopedic surgery	1083	2040	2167	1888	1736	1445	1403	1400	929	593	1062	806	1023	701
Teaching hospitals	4261	4263	4493	4255	3939	3967	4482	4508	3634	3530	3634	3392	3276	2616
General hospitals	2963	3079	3210	3151	3353	2711	1832	1851	1683	1407	1447	1616	1510	1055
Private clinics	8	960	1152	1634	1561	1589	1822	1775	1289	750	1495	1305	1604	1557
University hospitals	354	371	349	363	319	294	307	291	266	244	252	209	195	116
**Patients age < 56 years**														

For peer review only - http://bmjopen.bmj.com/site/about/guidelines.xhtml

Number of procedures	7192	8052	8363	8683	8383	7708	7414	7742	6827	6052	6691	6243	6484	5184
Per 10,000 inhabitants	8.6	9.6	10.0	10.4	10.0	9.2	8.9	9.3	8.3	7.3	8.1	7.6	7.8	6.2
Per 10,000 hospital visitors	1103	1187	1204	1181	1139	1200	1139	1183	1107	971	1028	969	1016	977
Male	3881	4386	4489	4638	4486	4060	3876	4011	3583	3019	3426	3105	3274	2540
Female	3311	3666	3874	4045	3897	3648	3538	3731	3244	3033	3265	3138	3210	2644
Neurosurgery	6198	6376	6632	7152	7054	6619	6371	6633	6085	5575	5807	5625	5694	4732
Orthopedic surgery	1230	2010	2059	1927	1711	1416	1425	1509	1093	753	1242	922	1158	754
Teaching hospitals	4026	3962	4098	3989	3651	3589	3868	4091	3434	3435	3362	3141	3150	2401
General hospitals	2703	2786	2846	2802	2927	2305	1589	1672	1759	1536	1538	1521	1443	1077
Private clinics	58	864	994	1467	1415	1404	1599	1582	1290	758	1473	1304	1625	1509
University hospitals	405	440	425	425	390	410	358	397	344	323	318	277	266	197

For peer review only - http://bmjopen.bmj.com/site/about/guidelines.xhtml

Table 3. Time between diagnosis, first hospital-visit and surgery between 2012 and 2020 (Nivel and NZA database)

	2012 (n=697)	2013 (n=958)	2014 (n=1174)	2015 (n=986)	2016 (n=804)	2017 (n=947)	2018 (n=902)	2019 (n=1031)	2020 (n=744)
Days between first GP[b] visit and first hospital visit (average)	158	155	163	179	180	174	184	169	193
**First GP visit to surgery**									
Days between first GP visit and surgery, median (25th-75th)	154 (80 – 361)	154 (77 – 328)	166 (87 – 351)	183 (91 – 361)	174 (92 – 371)	187 (103 – 354)	202 (104 – 386)	196 (104 – 383)	239 (137 – 451)
Days between first GP visit and surgery, 5th-95th	15 - 695	22 - 756	29 - 795	34 - 803	26 - 822	35 - 824	35 - 857	36 - 803	43 - 840
Days between first GP visit and surgery for patients <56 years, median (25th-75th)	137 (74-332)	147 (71-310)	142 (79-280)	167 (82-341)	161 (86-337)	160 (85-301)	172 (91-346)	183 (91-347)	230 (111-415)
**Time from first hospital visit to surgery**									
Days between first hospital visit and surgery, median (25th-75th)	51 (25-91)	51 (25-91)	56 (26-99)	62 (32-106)	59 (29-96)	70 (35-118)	67 (35-119)	70 (35-125)	80 (40-141)
Days between first hospital visit and surgery, 5th-95th	2 - 223	6 - 207	7 - 223	7 - 201	8 - 212	8 - 223	7 - 223	7 - 239	8 – 264
Days between first hospital visit and surgery for patients <56 years, median (25th-75th)	40 (21-78)	45 (21-81)	45 (21-81)	48 (23-86)	46 (22-78)	63 (31-105)	56 (23-108)	59 (28-110)	63 (31-118)
**Following the guideline?**									
Patients that had surgery within 2 days from diagnosis (GP) ()	0.7	0.8	0.5	0.5	0.5	0.4	0.3	0.8	0.4
Patients that had surgery between 2 days and 12 weeks from diagnosis (GP) ()	26.2	26.3	23.2	22.1	21.8	18.3	17.3	17.6	12.4
Patients that had surgery after 12 weeks and before 20 weeks from diagnosis (GP) ()	20.2	18.6	19.7	16.3	19.4	17.3	17.3	15.7	13.0
Patients that had surgery after 20 weeks from diagnosis (GP) ()	52.8	54.3	56.4	61.1	58.3	63.9	65.0	65.9	74.2

For peer review only

For peer review only - http://bmjopen.bmj.com/site/about/guidelines.xhtml

Patients <56 years that had surgery within 2 days from diagnosis (GP) ()	1.4	0.8	0.3	0.7	0.3	0.5	0.3	1.1	0.0
Patients <56 years that had surgery between 2 days and 12 weeks from diagnosis (GP) ()	30.7	28.7	26.8	25.5	24.6	24.3	20.3	20.0	17.2
Patients <age 56 years that had surgery after 12 weeks and before 20 weeks from diagnosis (GP) ()	18.9	18.8	22.0	15.1	20.3	17.4	19.0	17.2	14.3
Patients <age 56 years that had surgery after 20 weeks from diagnosis (GP) ()	49.0	51.7	50.8	58.7	54.8	57.7	60.4	61.6	68.5
Waiting time for surgery (days)	31	29	32	34	36	39	38	43	44

[a] number of surgical treated patients that could be linked between the NIVEL and NZA database [b] General Practitioner

For peer review only

For peer review only - http://bmjopen.bmj.com/site/about/guidelines.xhtml

For peer review only

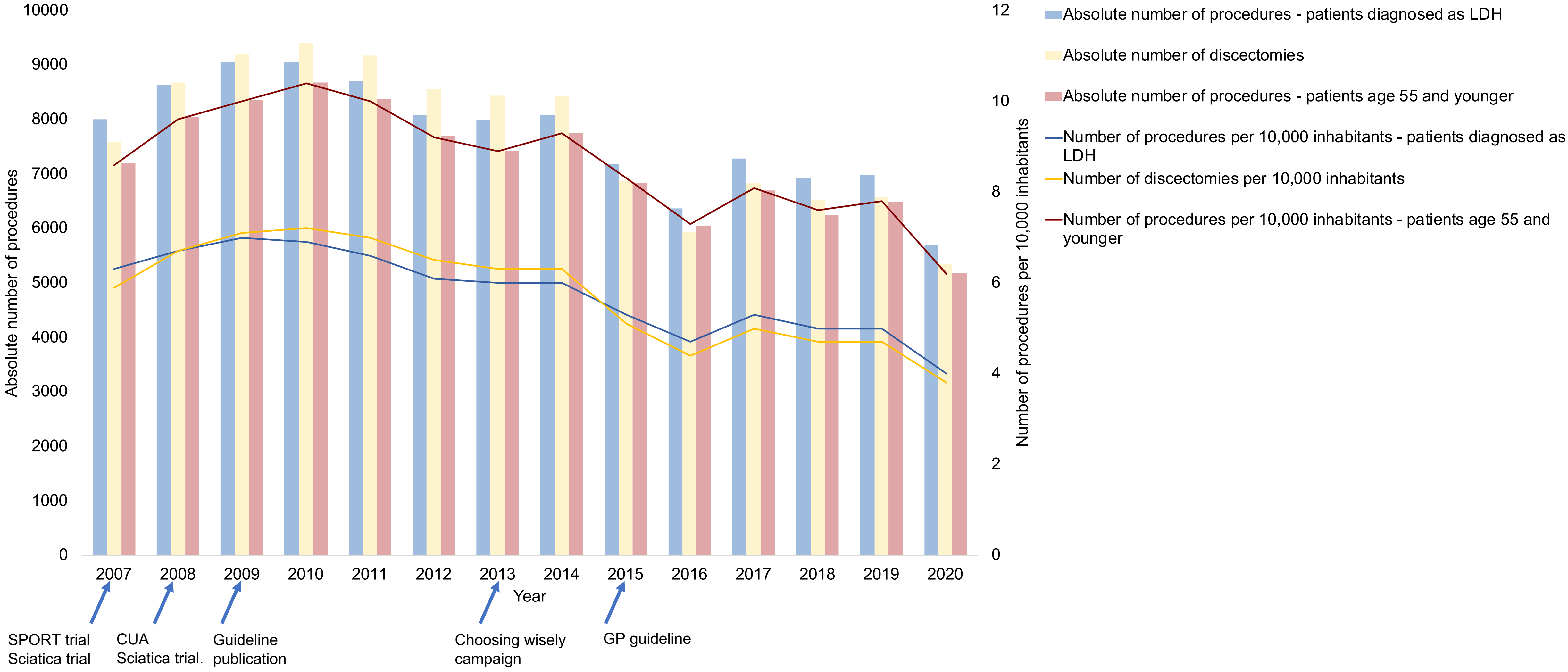
10000
9000
8000
7000
6000
5000
4000
3000
2000
1000
0
Absolute number of procedures
12
10
8
6
4
2
0
Number of procedures per 10,000 inhabitants
2007
2008
2009
2010
2011
2012
2013
2014
2015
2016
2017
2018
2019
2020
Year
SPORT trial Sciatica trial
CUA Sciatica trial.
Guideline publication
Choosing wisely campaign
GP guideline
Absolute number of procedures - patients diagnosed as LDH
Absolute number of discectomies
Absolute number of procedures - patients age 55 and younger
Number of procedures per 10,000 inhabitants - patients diagnosed as LDH
Number of discectomies per 10,000 inhabitants
Number of procedures per 10,000 inhabitants - patients age 55 and younger

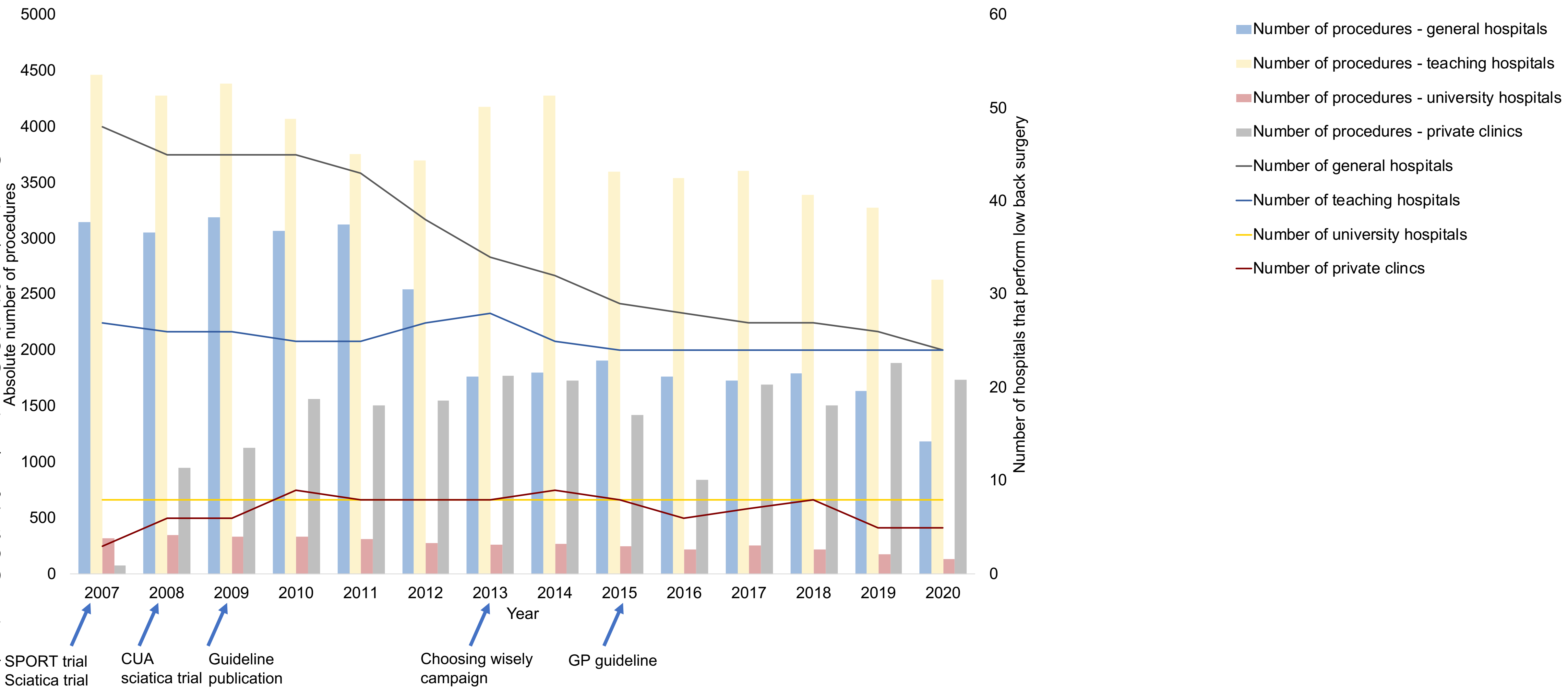
5000
4500
4000
3500
3000
2500
2000
1500
1000
500
0
Absolute number of procedures
60
50
40
30
20
10
0
Number of hospitals that perform low back surgery
2007
2008
2009
2010
2011
2012
2013
2014
2015
2016
2017
2018
2019
2020
Year
Number of procedures - general hospitals
Number of procedures - teaching hospitals
Number of procedures - university hospitals
Number of procedures - private clinics
Number of general hospitals
Number of teaching hospitals
Number of university hospitals
Number of private clincs
SPORT trial
Sciatica trial
CUA
sciatica trial
Guideline
publication
Choosing wisely
campaign
GP guideline

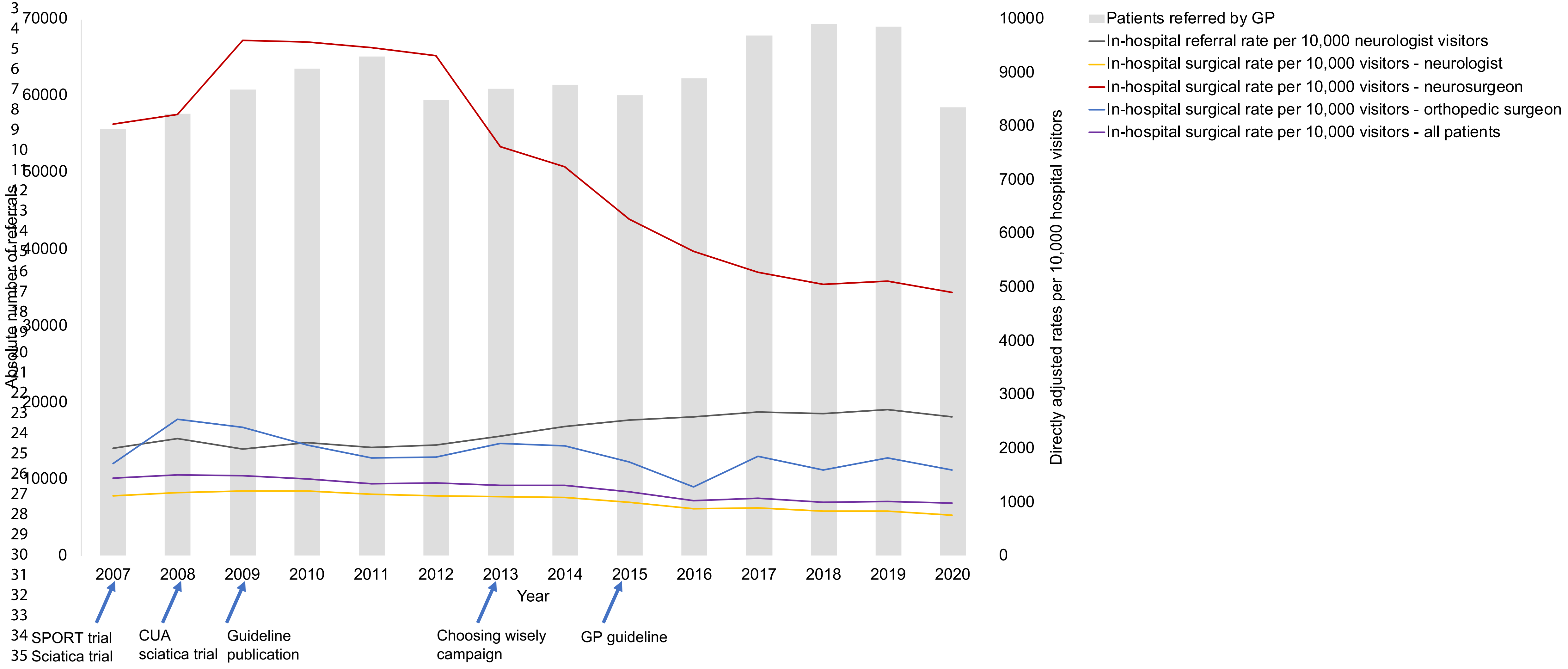
70000
60000
50000
40000
30000
20000
10000
0
Absolute number of referrals
10000
9000
8000
7000
6000
5000
4000
3000
2000
1000
0
Directly adjusted rates per 10,000 hospital visitors
2007
2008
2009
2010
2011
2012
2013
2014
2015
2016
2017
2018
2019
2020
Year
Patients referred by GP
In-hospital referral rate per 10,000 neurologist visitors
In-hospital surgical rate per 10,000 visitors - neurologist
In-hospital surgical rate per 10,000 visitors - neurosurgeon
In-hospital surgical rate per 10,000 visitors - orthopedic surgeon
In-hospital surgical rate per 10,000 visitors - all patients
SPORT trial
Sciatica trial
CUA
sciatica trial
Guideline
publication
Choosing wisely
campaign
GP guideline

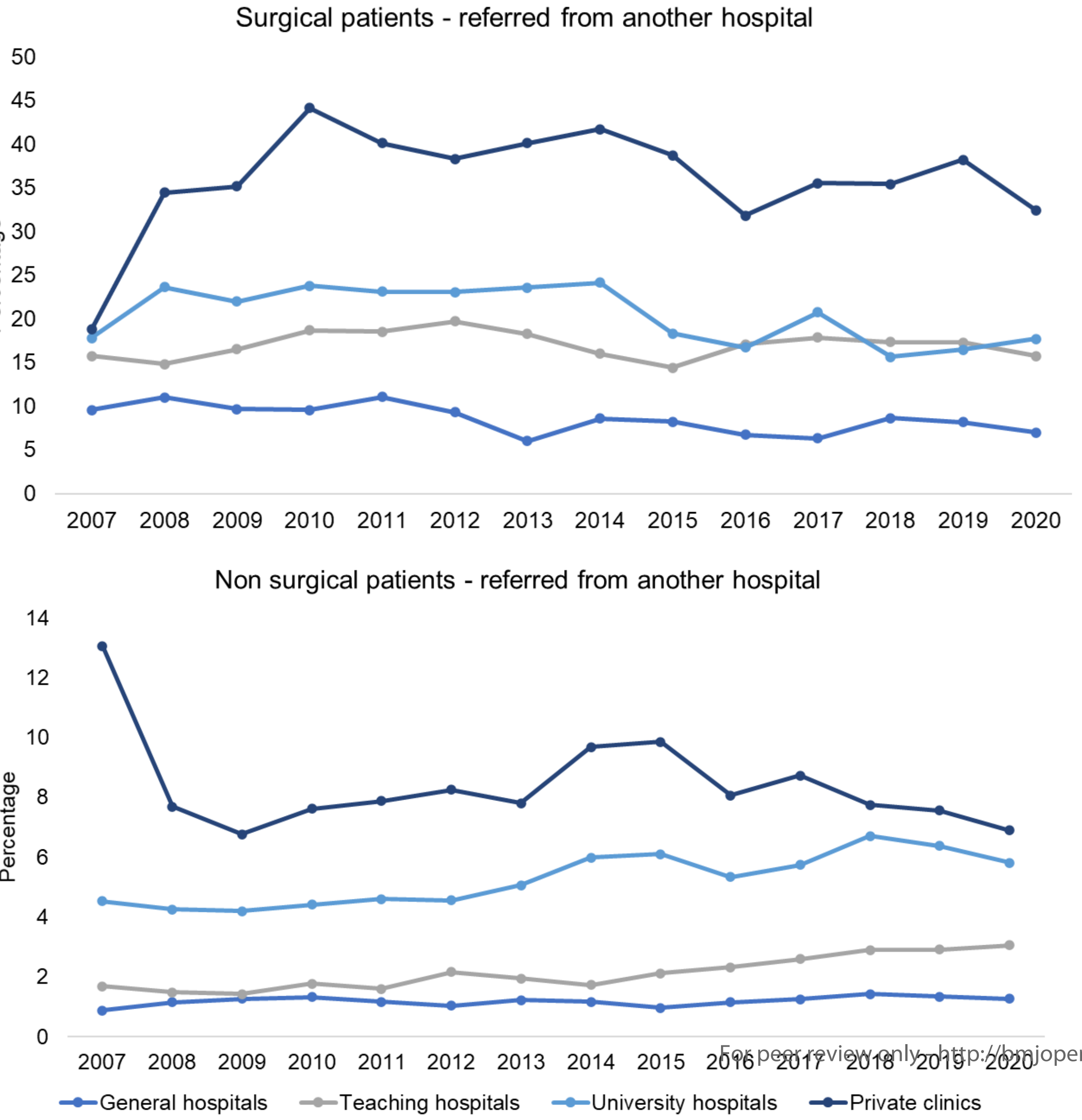

For peer review only - http://bmjopen.bmj.com/site/about/guidelines.xhtml

APPENDIX

Table S1. Used diagnosis treatment codes

Diagnosis treatment codes	Description
Included	
0330-12-00-1203	Pseudo radicular syndrome
0330-12-00-1204	Radicular syndrome
0330-12-00-1211<br>0305-13-00-1350	Spinal stenosis
0305-13-00-1360	Hernia Nuclei Pulposis
0305-13-00-1370	Spondylolisthesis
0308-02-25-2501<br>0308-02-25-2550<br>0308-02-25-2565<br>0308-02-25-2555<br>0308-02-25-2505<br>0308-02-25-2510<br>0308-02-25-2515	Degenerative disc disease
0330-12-00-1231<br>0305-13-00-1330<br>0305-13-00-1340	Low backpain
Excluded	
0330-12-00-1201<br>0330-12-00-1202<br>0330-05-00-0543<br>0305-12-00-1202<br>0305-12-00-1220<br>0305-12-00-1240<br>0308-02-25-2525<br>0308-02-25-2527<br>0308-02-25-2530<br>0308-02-25-2535	Cervical diagnosis codes
0330-02-00-0232<br>0330-02-00-0233<br>0330-02-00-0221<br>0330-02-00-0222<br>0330-02-00-0223<br>0308-02-21-2105<br>0308-02-21-2110	Tumors, malignancies of the spine

For peer review only

0308-02-21-2115	
0308-02-24-2405<br>0308-02-24-2411<br>0305-13-00-1301<br>0305-13-00-1302	Infectious spine disease
0305-13-00-1396<br>0308-02-23-2305<br>0308-02-23-2311	Spine trauma, fractures
0305-13-00-1397<br>0305-13-00-1380<br>0305-13-00-1381<br>0305-13-00-1383<br>0305-13-00-1391	Congenital disc disease, kyphosis, scoliosis

Table S2. Used care product codes

**Care product code**	**Description**
Included	
38437<br>38444<br>38438<br>38467	Discectomy
30327<br>30329	Laminectomy
38458<br>38459<br>38460<br>38464<br>38468<br>38469	Fusion

For peer review only

	2007	2008	2009	2010	2011	2012	2013	2014	2015	2016	2017	2018	2019	2020
**Patients with a registered diagnosis of LDH[a]**														
Hospital visitors	55581	57597	60777	63493	65023	59365	60883	61396	60040	62217	67771	69296	68997	58442
Per 10,000 inhabitants	43	45	47	49	49	45	46	46	45	46	50	50	50	42
Male	28528	29674	31064	32271	32624	29446	29838	30190	29260	30104	32362	32836	32934	27733
Female	27053	27923	29713	31222	32399	29919	31045	31206	30780	32113	35409	36460	36063	30709
Neurology	48447	50803	53529	56499	57929	52870	54380	54382	53327	56397	60410	62255	62047	52314
Neurosurgery	8643	8409	7560	7970	7859	7522	9099	9644	10240	10487	12084	12444	12145	10648
Orthopedic surgery	7490	7944	8678	8437	8764	7435	6605	6954	6071	5097	6595	5840	5976	4843
Referred by neurologist to surgical department	7155	7526	7005	7506	7346	6896	7541	7790	7991	8180	9521	9457	9398	7838
Teaching hospitals	23509	23736	25298	25115	25964	24972	30872	30979	30855	31844	33391	34414	33453	28581
General hospitals	27714	26461	27189	28910	29079	24980	21083	22322	22026	22598	22525	22241	22224	18534
Private clinics	1774	4806	5797	6912	7461	7005	6518	5547	4963	5334	9394	10315	11097	9301
University hospitals	2584	2594	2493	2556	2519	2408	2410	2548	2196	2441	2461	2326	2223	2026
<u>Mean per hospital</u>														
Teaching hospitals	871	913	973	966	999	892	1065	1192	1234	1274	1336	1377	1338	1143
General hospitals	462	448	486	535	539	480	449	507	551	595	644	635	635	545
Private clinics	197	267	290	288	298	280	251	264	216	254	447	491	555	443
University hospitals	323	324	312	320	315	301	301	319	275	305	308	291	278	253
**Patients age < 56 years**														
Hospital visitors	65185	67861	69449	73503	73569	64257	65093	65463	61695	62308	65056	64399	63808	53077
Per 10,000 inhabitants	78	81	83	88	88	77	78	79	75	75	79	78	77	64
Male	32213	33336	34010	35468	35050	30228	30358	30501	28412	28623	29788	29004	28938	23817
Female	32972	34525	35439	38035	38519	34029	34735	34962	33283	33685	35268	35395	34870	29260
Neurology	48448	51585	54337	57659	57969	50346	50583	50188	47716	48789	50783	50658	11571	40686
Neurosurgery	8902	8747	7131	7665	7615	7342	9499	10158	10629	10300	11270	11415	49746	10581
Orthopedic surgery	15865	16559	16607	17370	17256	14589	13514	14121	11813	11584	12328	11343	11537	9144
Referred by neurologist to surgical department	5164	5562	5205	5574	5507	5021	5410	5488	5414	5281	6071	5928	5763	4656
Teaching hospitals	27871	28569	29173	30248	30182	27595	33030	32593	31141	31510	32770	32911	31988	25935
General	31533	30973	31641	33444	33092	26749	21881	22868	21473	21901	21370	20161	19668	15801
University hospitals	3969	4037	3655	3541	3437	3224	3153	3200	2873	3218	2897	2548	2480	2101
Private clinics	1812	4282	4980	6270	6858	6689	7029	6802	6208	5679	8019	8779	9672	9240
<u>Mean per hospital</u>														
Teaching hospital	214	284	311	338	337	317	332	372	348	348	413	425	467	422
General	513	525	565	627	620	527	482	523	533	574	628	589	586	491
University hospital	1072	1106	1133	1051	1020	884	1070	1226	1300	1314	1262	1225	1347	1006
Private clinics	496	505	457	443	430	403	394	400	359	402	362	319	310	263
[a] Lumbar Disc Herniation														

Table S3. Trends between 2007 and 2020 for hospital visitors with a likely diagnosis of lumbar disc herniation

For peer review only

For peer review only

STROBE Statement—Checklist of items that should be included in reports of ***cross-sectional studies***

	Item No	Recommendation
**Title and abstract**	1	(*a*) Indicate the study's design with a commonly used term in the title or the abstract (p1, l1)
		(*b*) Provide in the abstract an informative and balanced summary of what was done and what was found (p2)
**Introduction**		
Background/rationale	2	Explain the scientific background and rationale for the investigation being reported (p3)
Objectives	3	State specific objectives, including any prespecified hypotheses (p3, p4; l89-94)
**Methods**		
Study design	4	Present key elements of study design early in the paper (p4; l96)
Setting	5	Describe the setting, locations, and relevant dates, including periods of recruitment, exposure, follow-up, and data collection (p4; section data sources)
Participants	6	(*a*) Give the eligibility criteria, and the sources and methods of selection of participants (p4; section population)
Variables	7	Clearly define all outcomes, exposures, predictors, potential confounders, and effect modifiers. Give diagnostic criteria, if applicable (p5; section analyses)
Data sources/ measurement	8*	For each variable of interest, give sources of data and details of methods of assessment (measurement). Describe comparability of assessment methods if there is more than one group (p5, p6; section data collection, analyses, costa analysis and analytic approach)
Bias	9	Describe any efforts to address potential sources of bias (p5, p6; section analyses, cost analysis and analytic approach)
Study size	10	Explain how the study size was arrived at (n.a. (population-based))
Quantitative variables	11	Explain how quantitative variables were handled in the analyses. If applicable, describe which groupings were chosen and why (p6; section analytic approach)
Statistical methods	12	(*a*) Describe all statistical methods, including those used to control for confounding (p6; analytic approach)
		(*b*) Describe any methods used to examine subgroups and interactions (n.a.)
		(*c*) Explain how missing data were addressed (we had no missing data due to the administrative study design)
		(*d*) If applicable, describe analytical methods taking account of sampling strategy (n.a.)
		(*e*) Describe any sensitivity analyses (n.a.)
**Results**		
Participants	13*	(a) Report numbers of individuals at each stage of study—eg numbers potentially eligible, examined for eligibility, confirmed eligible, included in the study, completing follow-up, and analysed (p6; section study population)
		(b) Give reasons for non-participation at each stage (n.a.)
		(c) Consider use of a flow diagram (n.a.)
Descriptive data	14*	(a) Give characteristics of study participants (eg demographic, clinical, social) and information on exposures and potential confounders (p6; section study population)
		(b) Indicate number of participants with missing data for each variable of interest (n.a.)
Outcome data	15*	Report numbers of outcome events or summary measures (p7; l179 – p8 l211)

For peer review only - http://bmjopen.bmj.com/site/about/guidelines.xhtml

Main results	16	(*a*) Give unadjusted estimates and, if applicable, confounder-adjusted estimates and their precision (eg, 95% confidence interval). Make clear which confounders were adjusted for and why they were included (n.a. due to the study design)
		(*b*) Report category boundaries when continuous variables were categorized (n.a.)
		(*c*) If relevant, consider translating estimates of relative risk into absolute risk for a meaningful time period (not relevant)
Other analyses	17	Report other analyses done—eg analyses of subgroups and interactions, and sensitivity analyses (p8; section cost analysis)
**Discussion**		
Key results	18	Summarise key results with reference to study objectives (p8; l218-l224)
Limitations	19	Discuss limitations of the study, taking into account sources of potential bias or imprecision. Discuss both direction and magnitude of any potential bias (p10; l283-l294)
Interpretation	20	Give a cautious overall interpretation of results considering objectives, limitations, multiplicity of analyses, results from similar studies, and other relevant evidence (p11; section conclusion)
Generalisability	21	Discuss the generalisability (external validity) of the study results (not important due to the population-based design (all inhabitants are included)
**Other information**		
Funding	22	Give the source of funding and the role of the funders for the present study and, if applicable, for the original study on which the present article is based (p1)

*Give information separately for exposed and unexposed groups.

Note: An Explanation and Elaboration article discusses each checklist item and gives methodological background and published examples of transparent reporting. The STROBE checklist is best used in conjunction with this article (freely available on the Web sites of PLoS Medicine at http://www.plosmedicine.org/, Annals of Internal Medicine at http://www.annals.org/, and Epidemiology at http://www.epidem.com/). Information on the STROBE Initiative is available at www.strobe-statement.org.

For peer review only

BMJ Open

the impact of evidence-based guidelines on healthcare utilization and costs for disc related sciatica in the Netherlands: a population-based, cross-sectional study

Journal:	*BMJ Open*
Manuscript ID	bmjopen-2023-078459.R1
Article Type:	Original research
Date Submitted by the Author:	27-Nov-2023
Complete List of Authors:	van Munster, Juliëtte; Leiden University, Department of Otorhinolaryngology and Head and Neck Surgery<br>Noordenbos, Mark; Dutch Healthcare Authority<br>Halperin, I.J.Y.; Leiden University, Department of Otorhinolaryngology and Head and Neck Surgery<br>van den Hout, Wilbert; Leiden University Medical Center, Medical Decision Making<br>van Benthem, Peter Paul; Leiden University Medical Center, Otorhinolaryngology - Head and Neck Surgery<br>Seinen, Ingrid; Dutch Healthcare Authority<br>Moojen, Wouter; Medical Center Haaglanden, Neurosurgery; HAGA hospital, Neurosurgery<br>Peul, Wilco; Leiden University Medical Center, Neurosurgery; Medical Center The Hague, Neurosurgery
**Primary Subject Heading**:	Evidence based practice
Secondary Subject Heading:	Epidemiology, General practice / Family practice, Neurology, Surgery
Keywords:	Clinical Decision-Making, Protocols & guidelines < HEALTH SERVICES ADMINISTRATION & MANAGEMENT, Neuromuscular disease < NEUROLOGY

SCHOLARONE™ Manuscripts

I, the Submitting Author has the right to grant and does grant on behalf of all authors of the Work (as defined in the below author licence), an exclusive licence and/or a non-exclusive licence for contributions from authors who are: i) UK Crown employees; ii) where BMJ has agreed a CC-BY licence shall apply, and/or iii) in accordance with the terms applicable for US Federal Government officers or employees acting as part of their official duties; on a worldwide, perpetual, irrevocable, royalty-free basis to BMJ Publishing Group Ltd ("BMJ") its licensees and where the relevant Journal is co-owned by BMJ to the co-owners of the Journal, to publish the Work in this journal and any other BMJ products and to exploit all rights, as set out in our licence.

The Submitting Author accepts and understands that any supply made under these terms is made by BMJ to the Submitting Author unless you are acting as an employee on behalf of your employer or a postgraduate student of an affiliated institution which is paying any applicable article publishing charge ("APC") for Open Access articles. Where the Submitting Author wishes to make the Work available on an Open Access basis (and intends to pay the relevant APC), the terms of reuse of such Open Access shall be governed by a Creative Commons licence – details of these licences and which Creative Commons licence will apply to this Work are set out in our licence referred to above.

Other than as permitted in any relevant BMJ Author's Self Archiving Policies, I confirm this Work has not been accepted for publication elsewhere, is not being considered for publication elsewhere and does not duplicate material already published. I confirm all authors consent to publication of this Work and authorise the granting of this licence.

For peer review only

Title: the impact of evidence-based guidelines on healthcare utilization and costs for disc related sciatica in the Netherlands: a population-based, cross-sectional study

Authors: Juliëtte J.C.M. van Munster[1,2] MD, Mark W. Noordenbos, PhD[3], I.J.Y. Halperin[1,2] MD, Wilbert B. van den Hout[4] PhD, Peter Paul G. van Benthem[2] MD, PhD, Ingrid Seinen, MSc[3], Wouter Moojen[1] MD, PhD, Wilco C. Peul[1,5] MD, PhD

[1] University Neurosurgical Centre Holland (UNCH), LUMC | HMC | HAGA, Leiden & The Hague, the Netherlands

[2] Department of Otorhinolaryngology and Head and Neck Surgery, Leiden University Medical Center, Leiden University, the Netherlands

[3] Dutch Healthcare Authority, Utrecht, the Netherlands.

[4] Department of Biomedical Data Sciences, Leiden University Medical Center, Leiden, the Netherlands

[5] Netherlands Organization for Health Research and Innovation (ZonMW)

Corresponding author:

Juliëtte van Munster, Dept. of Neurosurgery, Leiden University Medical Centre, P.O. Box 9600, 2300 RC Leiden, the Netherlands, Phone: +3171 526 5870, e-mail: j.j.c.m.van_munster@lumc.nl

Conflict of interest: Prof. dr. Peul: Boardmember ZonMW, former Chair Netherlands Choosing Wisely FMS committee

Prof. dr. van Benthem: Former president of the Federation Medical Specialists (FMS)

Dr. W. Moojen is a Board member of the Dutch Neurosurgical Society.

Supported by a grant from: the Netherlands Organization for Health Research and Development (Citrienfonds), grant number: 839205002. The funding organization had no role in the present study.

For peer review only

Prof. dr. Peul and dr. van den Hout were involved in the design and execution of the sciatica trial. Furthermore, prof dr. Peul was also involved in the revised Guideline construction and leader of the Netherlands Choosing Wisely campaign on behalf of FMS and ZonMW.

Ethical approval: our study was approved by the Medical Ethical Committee of Leiden and the Hague (metc-ldd@lumc.nl; N20.075)

Word count: 4194

Target journal: BMJ Open

For peer review only

ABSTRACT

Objective: The aim of this study was to assess the impact of high-quality evidence supporting surgical treatment of lumbar disc herniation (LDH) on healthcare practice in the Netherlands by examining changes in healthcare utilization, including the timing of surgery, and healthcare costs for LDH patients.

Design: A retrospective, cross-sectional study was performed utilizing population-based, longitudinal data obtained from the Dutch Healthcare Authority (2007-2020) and NIVEL's primary care (2012-2020) administrative databases.

Setting: The study was conducted within the healthcare system of the Netherlands.

Participants: We included adults (≥18 years) who visited a Dutch hospital or a General Practitioner for lumbar degenerative disc disease. LDH patients were identified based on registered diagnosis code, type of surgery (discectomy), and age (<56 years).

Main outcome measures: The primary outcome measure was the difference in the annual number of LDH procedures following the publication of the revised evidence-based guidelines in 2009 (comparing the periods 2007-2009 to 2017-2019). Secondary outcome measures focused on the timing of surgery and associated healthcare costs. To validate the outcomes, secondary outcomes also include number of discectomies and number of procedures in the younger age group (discectomies, laminectomies, and fusion surgery).

Results: Number of patients suffering from LDH increased from 55.581 to 68.997 (+24%) between 2007 and 2019. A decrease was observed in annual number of LDH procedures (-18%), in number of discectomies (-22%), and in number of procedures for patients age <56 years (-18%). This resulted in lower

healthcare costs by €12 million annually. In 2012, 31% of all patients <56 years had surgery before 12 weeks from diagnosis at the GP, whereas 20% did in 2019.

Conclusions: Healthcare utilization for LDH changed tremendously in the Netherlands between 2007 and 2020, coincided with the publication and implementation of evidence-based guidelines. The observed decrease in the number of procedures has been accompanied by a corresponding reduction in healthcare costs. These findings underscore the importance of adhering to evidence-based guidelines to optimize the management of LDH patients.

Strengths and limitations of this study

- A strength of this study is the nationwide coverage of the database over a considerable number of years.
- Sciatica patients were identified by analyzing three patient identifiers (diagnosis code, age, and type of surgery) to validate our findings and minimize administrative errors.
- Furthermore, policy makers and healthcare professionals performed this study together, supporting the independence of the study results.
- A limitation of the study is that causation between guideline publication, introduction of wise choices and the changes in healthcare practice cannot be proven due to the observational design and lack of data before 2007.

INTRODUCTION

Lumbosacral Radicular Syndrome (LRS), or sciatica due to a lumbar disc herniation (LDH) is common in everyday healthcare practices with a substantial burden for healthcare and society(1). Intolerable pain and cauda equina syndrome are definite indications for surgery, but most patients suffering from sciatica recover without the need of surgery within three months from onset(2), after which period the natural course is less favorable with a longer wait-and-see strategy(3).

For peer review only

In 1999, in response to a provoking practice variation publication(4), the Health Council of the Netherlands published an evidence-based report on this topic and concluded that there was lack of evidence on effectiveness and timing of surgery, but also serious signs of overtreatment in the Netherlands compared to the United Kingdom (UK)(5). In response, a Randomized Controlled Trial was designed and performed on this topic in the Netherlands (Sciatica trial; 2007)(6). Comparing the Netherlands with UK guidelines in timing of discectomy, patients suffering from severe sciatica for 6 to 12 weeks were randomly assigned to a timing of early surgery or to prolonged conservative treatment up to 6 months with possible delayed surgery. Around the same time, the SPORT trial (Weinstein et al.; 2006) was performed in the United States of America (USA)(7). Both trials showed no more differences in pain scores beyond one year, but with faster initial pain relief and better quality-adjusted life years after early surgery compared to conservative treatment(6, 8). As a result, early surgery was a cost-effective option that need not to be withheld for economic reasons, although it was associated with higher healthcare costs(9).

These trials led to the development of the first multidisciplinary, evidence-based guideline for LRS in the Netherlands in 2009(10) and several other countries(11, 12). An initial conservative treatment strategy is recommended in the first three months after onset of sciatica (in absence of progressive neurological deficit and cauda equina syndrome), whereas a shared decision-making treatment strategy between patient and physician is advised after three months. Guideline recommendations were incorporated in 'Wise Choices' in the Netherlands version of the North American initiated Choosing Wisely campaign in 2014(13, 14). In these 'Wise Choices', besides the advice that wait-and-see for a few months is advisable in sciatica, it is also advised not to wait too long with the risk of chronic pain but to proceed to surgery within 9 months after disease onset. These recommendations were integrated in the General Practitioner (GP) guideline in 2015(15).

It was decided by the original researchers in collaboration with the Federation Medical Specialists (FMS) and the Dutch Healthcare Authority that now is the right time to evaluate whether the evidence-based guideline recommendations changed healthcare utilization in the Netherlands between 2007 and 2020. We focused on number of procedures, timing of surgical treatment for LDH inflicted sciatica, and healthcare pathways. Secondary, we were interested in changes in societal and healthcare costs.

For peer review only

METHODS

A population-based, longitudinal, cross-sectional study was performed. The objective of this analysis was to evaluate the impact of guideline implementation on patients with sciatica attributed to lumbar disc herniation (LDH).

Data sources

Two databases were analyzed; firstly, a comprehensive nationwide, administrative healthcare database provided by the Dutch Healthcare Authority. This database contains detailed information on diagnosis by diagnosis-related groups, healthcare activities and fundamental patient characteristics (e.g. gender, year of birth) covering all patients treated in hospitals throughout the Netherlands(16). Secondly, NIVEL's (Netherlands Institute for Health Services Research) Primary Care Database was analyzed. This database comprises routine healthcare data recorded between 2012 and 2020 and represents a 10% sample of all general practitioner (GP) practices in the country (17). To ensure patient confidentiality, individuals in both databases were linked using a unique anonymized identifier, thereby preventing access to sensitive personal patient data by the researchers.

Population

This study focused on Dutch individuals aged 18 years and above who sought medical care at hospitals for sciatica due to a lumbar disc herniation between 2007 and 2020. The authors hypothesized that these patients would more often possess a registered diagnosis of LDH, have undergone discectomy as a treatment modality, and belong to a younger age group (<56 years) in comparison to individuals with spinal stenosis—a degenerative condition commonly observed in the elderly. The latter hypothesis was based on the age distribution of patients included in the SPORT trial and Sciatica trial(6, 18).However, to mitigate potential data incompleteness, as different healthcare providers may employ varying registration practices, the initial study group incorporated multiple hospital diagnosis codes for lumbar degenerative disc disease (DDD), including the code for spinal stenosis, and corresponding surgical care products (discectomy, laminectomy, and instrumented spinal fusion) (Table S1 and S2). Exclusion criteria included patients with cervical degenerative disc disease, spinal infection, traumatic or oncological spine fractures, spinal deformities, and those who had undergone previous back surgery within the past

For peer review only - http://bmjopen.bmj.com/site/about/guidelines.xhtml

year. From this initial study group, we selected the three subgroups for lumbar disc herniations based on type of surgery, age, and diagnosis code as stated above (For details, see table S3). Shortly, the lumbar disc herniation group was based on diagnosis code ‘1360’ (Hernia Nuclei Pulposi) using the same exclusion criteria as the initial study group and including all care products, whereas the discectomy group was based on the same inclusion and exclusion criteria for diagnosis as the initial study group, and the age group was based on the initial study group, but only patients aged younger than 56 years were included. In the NIVEL database, adult patients diagnosed with lumbo-radicular syndrome (coded as L86) were included.

Data collection

Firstly, annual number of hospital visits and procedures were collected. Furthermore, we collected patients’ age and gender, hospital type (general hospital, (top clinical) teaching hospital, university hospital, or private clinic), attending medical specialist (neurology, neurosurgery, or orthopedic surgery), date of first hospital visit, date of surgery and information on hospital waiting time for hernia-related procedures (days between order request and day of procedure). Surgical patients were assigned to the hospital where the surgery was performed. Non-surgical patients were assigned to the last hospital they visited. From the NIVEL database, date of first visit to the GP was collected.

Analyses

Our primary outcome included all patients with a registered diagnosis of LDH. Absolute numbers of diagnoses and procedures were calculated between 2007 and 2020. To provide a more accurate representation, surgical rates were adjusted based on the population of individuals aged 18 years and older residing in the Netherlands. Adjusted surgical rates were calculated per 10,000 inhabitants, thereby normalizing the data. Additionally, we calculated in-hospital surgical rates to assess the proportion of referrals with an indication for surgical intervention. Hospital visitors for the primary outcome only included visitors with a registered LDH diagnosis code. Secondary outcomes to validate our findings included the number of discectomies and the number of procedures

For peer review only

For peer review only - http://bmjopen.bmj.com/site/about/guidelines.xhtml

(discectomies, laminectomies, and fusion) in the age group < 56 years, for which hospitals visitors included all LDD patients. Directly adjusted surgical rates per 10,000 inhabitants or 10,000 hospital visitors were calculated using the following formula:

$$\frac{Number\ of\ surgical\ procedures}{Number\ of\ inhabitants\ \geq 18\ years\ /\ hospital\ visitors} \times 10{,}000$$

Furthermore, the timing of surgical interventions was analyzed by calculating the median number of days between the initial visit to the general practitioner (GP) and the date of surgery. Lastly, we assessed the median number of days between first visit hospital consultation and date of surgical procedure.

Cost analysis

Differences in national healthcare and societal costs were calculated by comparing the annual number of LDH patients per 10,000 over 2007-2009 to that number over pre-covid years 2017-2019. We updated our previous economic evaluation(9) to the population size and price level in 2022, using current surgical prices and updating other costs by the Dutch consumer price index(16, 19). Thus, the difference between a surgical and a conservative policy was estimated at €2596 in healthcare costs and €107 in societal costs per patient. With a 49% difference in surgery probability between both policies(9), these estimates translate to the slightly more than double €5298 in healthcare savings and €218 societal savings per prevented surgery. Societal savings are negligible, because the conservative policy has higher productivity costs.

Analytic approach

All analyses were conducted at the individual patient level. To assess differences in annual healthcare utilization, a comparison was made between the years preceding the publication of the guidelines (2007-2009) and the most recent years (2017-2019). Due to the limited usefulness of the data from the COVID-impacted year 2020, it was included in the database but not used for direct comparisons. However, the inclusion of 2020 was deemed important to demonstrate the potential impact of the pandemic on healthcare utilization. A larger decrease in procedures in 2020 might reflect undertreatment, which can

For peer review only

lead to chronic disability of patients waiting too long with persistent sciatica leading to chronic disease(3). We chose for a descriptive analysis, considering the anticipated statistical significance of even small differences given the large size of the database. All analyses were performed using R version 4.1.3(20).

Patient and public involvement in research

Patients and public were not involved in this research.

For peer review only

RESULTS

Study population

During the period from 2007 to 2020, a total of 1.9 million patients sought hospital care for lumbar degenerative disc disease (DDD). Median age of these patients was 56 years and 54% was female (Table 1). Among these patients, 12% underwent discectomy, laminectomy or fusion surgery (n=217.166). The most common surgical procedure was a discectomy (6%) or laminectomy (6%), while instrumented spinal fusion was less frequently utilized. In 53% of all cases, surgical patients were older than 56 years old and 51% was male. Teaching hospitals accounted for 52% of all procedures. Between 2012 and 2020, the NIVEL database, specifically the L86 code representing lumbo-radicular syndrome, matched with the hospital database, resulting in 52,057 patients being included in the analysis. Of these patients, 53% was female (compared to 54% in the hospital database), and the mean age was 56 years (compared to 52.4 in 2007 and 57.6 in 2020 in the hospital database). Moreover, among these patients, 16% (8,267 individuals) underwent surgery, showing a slightly higher surgical rate compared to the hospital database, which reported a 12% surgical rate.

Changes in healthcare utilization between 2007-2009 and 2017-2019

Absolute number of procedures for sciatica patients decreased 18% in the LDH group, 22% in the discectomy group, and 18% in the age <56 years group, although an initial increase in number of procedures was observed in all three groups between 2007 and 2010 (Figure 1, Table 2 and Table S4). Annual

For peer review only - http://bmjopen.bmj.com/site/about/guidelines.xhtml

number of surgical procedures for LDH patients in private clinics increased from 719 in the years 2007-2009 to 1699 in the years 2017-2019 (Figure 2, Table 2 and table S4). Number of LDH patients in teaching hospitals increased as well (+40%), whereas numbers of surgical procedures for LDH patients decreased in teaching hospitals (-22%). Number of visitors and procedures for LDH patients decreased in general hospitals (-18%; -45%) and university hospitals (-9%; -35%) in the period 2017-2019 compared to 2007-2009.

Changes in healthcare pathways between 2007 and 2019

The number of hospital visitors diagnosed with LDH increased from 55,581 in 2007 to 68,997 in 2019, as shown in Figure 3 and detailed in Table S6. Concurrently, the absolute number of LDH patients referred by neurologists to surgical departments rose from 7,155 in 2007 to 9,398 in 2019, representing a 31% increase over the study period. However, the surgical rate per 10,000 LDH hospital visitors declined from 1,441 per 10,000 in 2007 to 1,012 per 10,000 in 2019 (Figure 2a, Table 2 and Table S4). Notably, this decrease was more pronounced among neurosurgeons, dropping from 80% in 2007 to 51% in 2019, compared to orthopedic surgeons, whose surgical rate decreased from 11% in 2007 to 8% in 2019. In terms of referrals between hospitals for surgical and non-surgical LDH patients, there was no substantial change observed between 2007 and 2020 across different hospital types, as depicted in Figure 4.

Timing of surgery between 2012 and 2020

The median duration in days between the initial diagnosis at the general practitioner (GP) and the date of surgery exhibited an increase from 154 days in 2012 to 196 days in 2019, as shown in Table 3 and Table S5. However, the time interval between the first GP visit and the first hospital visit, also known as the time to referral, remained stable (155 days). Moreover, days between first hospital visit and date of surgery increased from 71 days to 93 days (+22 days). Concurrently, the waiting time for hernia-related procedures in hospitals also increased, with an interval of 31 days in 2012 expanding to 43 days in 2019, reflecting a 12-day prolongation. Among patients below the age of 56 years, there was a decrease in the percentage of individuals who underwent surgery within 12 weeks from the time of diagnosis, declining from 31% in 2012 to 20% in 2019. Conversely, there was an increase in the proportion of patients who

For peer review only - http://bmjopen.bmj.com/site/about/guidelines.xhtml

received surgery after 20 weeks from diagnosis, rising from 49% in 2012 to 62% in 2019. These findings highlight a shift towards longer waiting periods for surgical intervention in this patient group.

Cost analysis

Changes in healthcare utilization for sciatica due to LDH led to an estimated decrease of 1.5 per 10,000 inhabitants aged 18 years and older living in the Netherlands. For 2022, the associated savings were estimated at a considerable 10 million euro on healthcare costs and only 0.5 million euro on societal costs.

DISCUSSION

Immediately following the publication of the Sciatica trial(6), an initial increase in surgical procedures for LDH patients was observed in the Netherlands. However, after 2010, a gradual decrease in the total numbers of procedures occurred, resulting in substantial annual reductions in healthcare costs and in societal costs. The largest decrease was observed between 2014 and 2016. Noteworthy changes in healthcare pathways revealed an increase in the number of referrals per surgeon, while the in-hospital surgical rate decreased. These findings suggest that both surgeons and patients are increasingly opting for an initial conservative treatment rather than early surgery.

Regarding the timing of disc surgery for sciatica, there were notable changes between 2012 and 2019. The median time interval between the initial GP visit and the date of surgery increased, resulting in 82% of all patients undergoing surgery more than 12 weeks after disease onset in 2019. It is important to note that the recommended time window for surgery, as advised in the 2009 Lumbo-Radicular Syndrome (LRS) guideline, indicating surgery at least 12 weeks after disease onset for the expectant wait-and-see period, was already met in 2012. Additionally, hospital waiting times for surgery also increased between 2012 and 2019, which does not necessarily align with surgeon’s treatment choices. Also, nerve root injections were not included in our analyses, but an increase might have influenced time until surgical treatment. Importantly, there was an increase in the percentage of patients who received surgery after more

For peer review only

For peer review only - http://bmjopen.bmj.com/site/about/guidelines.xhtml

than 20 weeks. Although not outlined in the current guideline, the Dutch choosing wisely campaign states that surgery should be performed between 3 and 9 months after disease onset. Although some patients might experience a longer waiting time, our results indicate that most people receive surgery within six months after disease onset. However, recent research showed that 20 weeks after disease onset, outcomes are significantly worse in the even more prolonged conservative care group beyond the guideline time window compared to surgical treatment(3). Therefore, a careful caution in patient guidance is warranted to prevent chronic disabling sciatica, due to irreversible damage to lumbosacral nerve roots by chronic compression(21). As this Canadian study was published during Covid times and not yet implemented in Dutch evidence-based guidelines(3), it is not possible to do a valid investigation in the proportion of patients with a too late indication for surgery. These study findings should be incorporate in a new set of guideline recommendations in the Netherlands.

Our findings are important, because the Netherlands used to perform the second-most operations of the lumbar spine per 100,000 inhabitants compared to other high-income countries(4). The number of procedures for LDH, however, increased in the first years after publication of the Sciatica trial(6). This increasing rate probably resulted from the published fact that study outcomes showed that surgery leads to faster pain relief compared to conservative treatment and was cost-effective compared to conservative care with possibly delayed surgery(9). The decrease after 2010 might have been initiated by the publication of guideline recommendations in 2009, but also by the observation of high practice variation in surgical treatment for LDH in the Netherlands in 2010(22), which reached the general public as this observation was published as a sign of low-value care in newspapers, leading to a task force of the medical specialist federation (FMS in collaboration with ZonMW) starting the Netherlands version of the international Choosing Wisely campaign. This campaign was launched in 2013 and potentially had a huge impact, since the largest decrease in number of procedures was observed between 2014 and 2016. The active dissemination of the guideline, collaboration of FMS/ZonmW with insurance companies and Patient Federation in the development of wise choices led to awareness of the favorable natural course in most sciatica patients. Not unimportantly the growing attention for evidence-based surgery(23) within the medical specialist scientific societies, probably speeded up the implementation of the guideline in clinical practice(24).

For peer review only

For peer review only - http://bmjopen.bmj.com/site/about/guidelines.xhtml

A notable shift in decision-making was observed among neurosurgeons and orthopedic surgeons, who increasingly adhered to the guideline recommendations. In contrast, general practitioners (GPs) and neurologists showed a tendency to refer even more patients with lumbar disc herniation (LDH) than before. Furthermore, the time interval between the first GP visit and the first hospital visit remained unchanged between 2012 and 2019. This observation may indicate that patients have a preference for consulting a surgeon to discuss treatment options for LDH or that healthcare delivery for LDH patients is increasingly organized through multidisciplinary LDH teams, as described by Michael Porter(25). Many hospitals in the Netherlands have implemented Integrated Practice Units, so called 'Herniated Disc-Diagnosis-Treatment Streets', where patients receive a joint appointment with both a neurologist and a surgeon, facilitating collaborative decision-making and comprehensive care.

In contrast to other hospital types, a tremendous increase in numbers of procedures for LDH patients was observed in private clinics. This might be attributed to a nationwide strategy aimed at redistributing and decentralizing high-volume, low-complexity diagnoses such as disc related sciatica from university and large teaching hospitals to private clinics(26). This approach helps to concentrate resources and expertise in the larger hospitals, which primarily handle more complex cases. This results in a relief of pressure on the large hospitals, which was especially important during the COVID-19 pandemic: care could be maintained for these patients that required urgent or emergency neurosurgical care(27).

A strength of this study is the nationwide coverage of the database. The fact that academic physicians performed this analysis together with policy makers from the Dutch Healthcare Authority is another strength of this study and supports the independence of the study results.

It must be mentioned, however, that two of the current supervising investigators were the Sciatica Trial's health economist (WH) and principal investigator (WP), while the latter was also involved in the Guideline construction and leader of the Netherlands Choosing Wisely campaign on behalf of FMS and ZonMW. Although both researchers were initially kept out of the data analysis of the current study, some influence might be inevitable. Another limitation of this study is the fact that it is not possible to prove causation between guideline publication and changes in healthcare utilization. Measuring the true impact of the Sciatica trial was challenging due to the unavailability of high-quality data prior to 2007. The most recent available data on the total number of lumbar disc surgeries for sciatica in the Netherlands dates back to 1996, with 11,323 patients per year(5). Similarly, in Finland the largest reduction in discectomies

For peer review only - http://bmjopen.bmj.com/site/about/guidelines.xhtml

occurred before 2007(28), probably caused by the starting awareness of ongoing studies in USA and Europe and the earlier landmark publication by Cherkin and Deyo warning against overtreatment in spine surgery(4). The last limitation of this study is the use of administrative healthcare data. The use of these data enables to perform this investigation, but results might reflect differences in registration or coding. For example, the GP code L86 might also include lumbar stenosis. By using the three patient identifiers (i.e. age, discectomy, and registered code for LDH), we tried to minimize this limitation and validate our findings.

CONCLUSION

Although causation cannot be proven, this study highlights the substantial impact of evidence-based guidelines on clinical practice. We observed a decrease in the number of discectomy procedures, with a longer timeframe between the initial visit to the general practitioner and surgery and subsequently a reduction in healthcare costs. The decrease in number of procedures suggest that the 2008 guideline recommendations for surgical treatment for sciatica resulting from lumbar disc herniation (LDH) were effectively implemented in clinical practice in the Netherlands, especially after the dissemination of evidence-based guidelines and the Choosing Wisely Campaign. The observed longer-time frame observed in this study, agrees with the current Dutch guideline, but recent research showed that waiting too long might lead to chronic disability. To ensure the continued improvement of quality of care, it is essential to incorporate the findings of studies such as Bailey et al. into evidence-based guidelines and clinical practice, thereby preventing undertreatment of LDH patients in the future.

For peer review only

Figure legends

Figure 1 trend in absolute number of procedures and the directly standardized number of procedures; the left Y-axis and bars show the absolute number of procedures. The right Y-axis and lines show the number of procedures per 10,000 inhabitants (age > 18 years).

Figure 2 trends in absolute number of procedures per hospital type. The left Y-axis and bars show the absolute number of surgical procedures, the right Y-axis and lines show the number of hospitals performing back surgeries.

247 Figure 3 GP referrals and in-hospital surgical rates. The left Y-axis and bars show the absolute number of surgical procedures, the right Y-axis and lines show the number of
248 procedures per 10,000 hospital visitors (in-hospital surgical rate; age 18 years and older).

249 Figure 4 number of referrals from another hospital for surgical and non-surgical patients per hospital type. The Y-axis shows the percentage of patients that were referred from
250 another hospital per hospital type.

For peer review only

REFERENCES

1. Ravindra VM, Senglaub SS, Rattani A, Dewan MC, Härtl R, Bisson E, et al. Degenerative Lumbar Spine Disease: Estimating Global Incidence and Worldwide Volume. Global Spine J. 2018;8(8):784-94.
2. Hofstee DJ, Gijtenbeek JM, Hoogland PH, van Houwelingen HC, Kloet A, Lötters F, et al. Westeinde sciatica trial: randomized controlled study of bed rest and physiotherapy for acute sciatica. J Neurosurg. 2002;96(1 Suppl):45-9.
3. Bailey CS, Rasoulinejad P, Taylor D, Sequeira K, Miller T, Watson J, et al. Surgery versus Conservative Care for Persistent Sciatica Lasting 4 to 12 Months. New England Journal of Medicine. 2020;382(12):1093-102.
4. Cherkin DC, Deyo RA, Loeser JD, Bush T, Waddell G. An international comparison of back surgery rates. Spine (Phila Pa 1976). 1994;19(11):1201-6.
5. Gezondheidsraad. Diagnostiek en behandeling van het radiculaire syndroom. In: Gezondheidsraad, editor. Den Haag1999.
6. Peul WC, van Houwelingen HC, van den Hout WB, Brand R, Eekhof JA, Tans JT, et al. Surgery versus prolonged conservative treatment for sciatica. N Engl J Med. 2007;356(22):2245-56.
7. Weinstein JN, Tosteson TD, Lurie JD, Tosteson AN, Hanscom B, Skinner JS, et al. Surgical vs nonoperative treatment for lumbar disk herniation: the Spine Patient Outcomes Research Trial (SPORT): a randomized trial. Jama. 2006;296(20):2441-50.
8. Weinstein JN, Tosteson TD, Lurie JD, Tosteson ANA, Hanscom B, Skinner JS, et al. Surgical vs Nonoperative Treatment for Lumbar Disk HerniationThe Spine Patient Outcomes Research Trial (SPORT): A Randomized Trial. JAMA. 2006;296(20):2441-50.
9. van den Hout WB, Peul WC, Koes BW, Brand R, Kievit J, Thomeer RT. Prolonged conservative care versus early surgery in patients with sciatica from lumbar disc herniation: cost utility analysis alongside a randomised controlled trial. Bmj. 2008;336(7657):1351-4.
10. CBO KvdG. Lumbosacraal Radiculair Syndroom 2008.
11. Krendl R. Lumbale Radikulopathie. Springer; 2014.
12. Kreiner DS, Hwang S, Easa J, Resnick DK, Baisden J, Bess S, et al., editors. Diagnosis and treatment of lumbar disc herniation with radiculopathy. North American Spine Society; 2012.
13. Verstandige keuzes bij een lage rughernia.
14. Levinson W, Kallewaard M, Bhatia RS, Wolfson D, Shortt S, Kerr EA. 'Choosing Wisely': a growing international campaign. BMJ Qual Saf. 2015;24(2):167-74.
15. Schaafstra AS, W.E.M.; Bons, S.C.S.; Borg, M.A.J.P.; Koes, B.W.; Ostelo, R.W.J.G.; Spijker-Huiges, A.; Burgers, J.S.; Bouma, M.; Verburg-Oorthuizen, A.F.E. Lumbosacraal radiculair syndroom. In: NHG-werkgroep, editor. 2015.
16. Statistics Netherlands Netherlands [Available from: https://www.cbs.nl/en-gb.
17. Netherlands Institute for Health Services Research [Available from: https://www.nivel.nl/en.
18. Cummins J, Lurie JD, Tosteson TD, Hanscom B, Abdu WA, Birkmeyer NJO, et al. Descriptive Epidemiology and Prior Healthcare Utilization of Patients in the Spine Patient Outcomes Research Trial's (SPORT) Three Observational Cohorts: Disc Herniation, Spinal Stenosis, and Degenerative Spondylolisthesis. Spine. 2006;31(7):806-14.

For peer review only - http://bmjopen.bmj.com/site/about/guidelines.xhtml

19. Hakkaart-van Roijen L, van der Linden N, Bouwmans C, Kanters T, Tan SS. Kostenhandleiding: Methodologie van kostenonderzoek enreferentieprijzen voor economische evaluatiesin de gezondheidszorg. Institute for Medical Technology Assessment, Erasmus Universiteit Rotterdam; 2012.
20. studio R. 4.1.3. ed2022.
21. Nygaard OP, Romner B, Trumpy JH. Duration of symptoms as a predictor of outcome after lumbar disc surgery. Acta Neurochir (Wien). 1994;128(1-4):53-6.
22. van Beek EL, K.; van Schooten, G.; Vlieger, E.J. Reduceren van praktijkvariatie: budgettaire effecten van scherpere indicatiestelling. In: Ministerie van Volksgezondheid WeS, editor. 2010.
23. McCulloch P, Altman DG, Campbell WB, Flum DR, Glasziou P, Marshall JC, et al. No surgical innovation without evaluation: the IDEAL recommendations. Lancet. 2009;374(9695):1105-12.
24. Lomas J. Words without action? The production, dissemination, and impact of consensus recommendations. Annu Rev Public Health. 1991;12:41-65.
25. Porter ME, Teisberg EO. Redefining competition in health care. Harv Bus Rev. 2004;82(6):64-76, 136.
26. Aandeel zelfstandige behandelcentra blijft toenemen 2018 [Available from: https://www.vektis.nl/actueel/aandeel-zelfstandige-behandelcentra-blijft-toenemen.
27. Ashkan K, Jung J, Velicu AM, Raslan A, Faruque M, Kulkarni P, et al. Neurosurgery and coronavirus: impact and challenges—lessons learnt from the first wave of a global pandemic. Acta Neurochirurgica. 2021;163(2):317-29.
28. Ponkilainen VT, Mäntymäki H, Huttunen TT, Mattila VM. Decreasing Incidence of Lumbar Discectomy Surgery in Finland in 1997-2018. Spine (Phila Pa 1976). 2021;46(6):383-90.

For peer review only

For peer review only - http://bmjopen.bmj.com/site/about/guidelines.xhtml

Table 1. baseline characteristics of the total lumbar degenerative disc disease population between 2007 and 2020

Characteristic	Total number of lumbar DDD patients (2007-2020, n=1863627 (100%[a]))	Surgically treated patients (2007-2020, n=217166 (100%))
**Age group (n, %)**		
18-45 years	523431 (28)	58648 (27)
46-55 years	391292 (21)	42370 (20)
56 years and older	948904 (51)	116098 (53)
**Gender, (n, %)**		
Male	856732 (46)	110922 (51)
Female	1006895 (54)	106194 (49)
**Diagnosis, (n, %)**		
Lumbar disc herniation	870878 (47)	108036 (50)
Lumbar stenosis	317360 (17)	65977 (30)
Lumbar disc herniation and lumbar stenosis	46050 (2)	31045 (14)
Low back pain	587695 (32)	4899 (2)
Unknown	41644 (2)	7159 (3)
**Hospital type, (n, %)**		
General hospital	736250 (40)	63892 (29)
Teaching hospital	884234 (47)	113444 (52)
University hospital	94071 (5)	13627 (6)
Private clinic	149072 (8)	26153 (12)
**Department, (n, %)**		
Neurology department	1464011 (79)	142786 (66)
Neurosurgery department	303626 (16)	189418 (87)
Orthopedic surgery department	356674 (19)	39172 (18)

For peer review only

**Type of treatment[b], (n, %)**		
Discectomy	107549 (6)	107549 (50)
Laminectomy	109477 (6)	109477 (9)
Fusion	20449 (1)	20449 (50)
No surgical treatment	1626152 (88)	-
**Referred from another hospital (n=1777532[c]), (n, %)**		
Yes	85164 (5)	44793 (26)
No	1692368 (95)	126835 (74)

[a] due to rounding, the number of percentages might not add up to 100% [b] due to the fact that patients might receive two types of surgery, the percentages might not add up to 100%

[c] Patients who visited more than one hospital on the same day were not included in this group

For peer review only

For peer review only - http://bmjopen.bmj.com/site/about/guidelines.xhtml

Table 2. Surgical trends between 2007 and 2020 for hospital visitors with a likely diagnosis of lumbar disc herniation

	2007	2009	2011	2013	2015	2017	2019
**Patients with a registered diagnosis of LDH[a]**							
Number of procedures	8009	9048	8708	7987	7183	7290	6983
Per 10,000 inhabitants	6.3	7.0	6.6	6.0	5.3	5.3	5.0
Per 10,000 hospital visitors	1441	1489	1339	1312	1196	1076	1012
Male	4278	4822	4645	4177	3802	3737	3567
Female	3731	4226	4063	3810	3381	3553	3416
Neurosurgery	6941	7250	7431	6928	6411	6381	6208
Orthopedic surgery	1282	2078	1602	1383	1063	1216	1088
Teaching hospitals	4463	4389	3757	4177	3598	3607	3277
General hospitals	3150	3193	3127	1769	1913	1732	1640
Private clinics	75	1132	1509	1775	1421	1698	1888
University hospitals	321	334	315	266	251	253	178
**Patients who had a discectomy**							
Number of procedures	7586	9204	9172	8443	6872	6828	6585
Per 10,000 inhabitants	5.9	7.1	7.0	6.3	5.1	5.0	4.7
Per 10,000 hospital visitors	656	719	658	652	524	472	442
Male	4084	4898	4944	4438	3676	3489	3348
Female	3502	4306	4228	4005	3196	3339	3237
Neurosurgery	6727	7380	7832	7463	6271	6153	5992
Orthopedic surgery	1083	2167	1736	1403	929	1062	1023
Teaching hospitals	4261	4493	3939	4482	3634	3634	3276
General hospitals	2963	3210	3353	1832	1683	1447	1510
Private clinics / University hospitals	8	1152	1561	1822	1289	1495	1604

For peer review only

	354	349	319	307	266	252	195
**Patients age < 56 years**							
Number of procedures	7192	8363	8383	7414	6827	6691	6484
Per 10,000 inhabitants	8.6	10.0	10.0	8.9	8.3	8.1	7.8
Per 10,000 hospital visitors	1103	1204	1139	1139	1107	1028	1016
Male	3881	4489	4486	3876	3583	3426	3274
Female	3311	3874	3897	3538	3244	3265	3210
Neurosurgery	6198	6632	7054	6371	6085	5807	5694
Orthopedic surgery	1230	2059	1711	1425	1093	1242	1158
Teaching hospitals	4026	4098	3651	3868	3434	3362	3150
General hospitals	2703	2846	2927	1589	1759	1538	1443
Private clinics	58	994	1415	1599	1290	1473	1625
University hospitals	405	425	390	358	344	318	266

For peer review only - http://bmjopen.bmj.com/site/about/guidelines.xhtml

Table 3. Time between diagnosis, first hospital-visit and surgery between 2012 and 2020 (Nivel and NZA database)

	2012 (n=697)	2013 (n=958)	2014 (n=1174)	2015 (n=986)	2016 (n=804)	2017 (n=947)	2018 (n=902)	2019 (n=1031)
Days between first GP[b] visit and first hospital visit (average)	158	155	163	179	180	174	184	169
**First GP visit to surgery**								
Days between first GP visit and surgery, median (25th-75th)	154 (80 – 361)	154 (77 – 328)	166 (87 – 351)	183 (91 – 361)	174 (92 – 371)	187 (103 – 354)	202 (104 – 386)	196 (104 – 383)
Days between first GP visit and surgery, 5th-95th	15 - 695	22 - 756	29 - 795	34 - 803	26 - 822	35 - 824	35 - 857	36 - 803
Days between first GP visit and surgery for patients <56 years, median (25th-75th)	137 (74-332)	147 (71-310)	142 (79-280)	167 (82-341)	161 (86-337)	160 (85-301)	172 (91-346)	183 (91-347)
**Time from first hospital visit to surgery**								
Days between first hospital visit and surgery, median (25th-75th)	51 (25-91)	51 (25-91)	56 (26-99)	62 (32-106)	59 (29-96)	70 (35-118)	67 (35-119)	70 (35-125)
Days between first hospital visit and surgery, 5th-95th	2 - 223	6 - 207	7 - 223	7 - 201	8 - 212	8 - 223	7 - 223	7 - 239
Days between first hospital visit and surgery for patients <56 years, median (25th-75th)	40 (21-78)	45 (21-81)	45 (21-81)	48 (23-86)	46 (22-78)	63 (31-105)	56 (23-108)	59 (28-110)
**Following the guideline?**								
Patients that had surgery within 2 days from diagnosis (GP) (%)	0.7	0.8	0.5	0.5	0.5	0.4	0.3	0.8
Patients that had surgery between 2 days and 12 weeks from diagnosis (GP) (%)	26.2	26.3	23.2	22.1	21.8	18.3	17.3	17.6
Patients that had surgery after 12 weeks and before 20 weeks from diagnosis (GP) (%)	20.2	18.6	19.7	16.3	19.4	17.3	17.3	15.7
Patients that had surgery after 20 weeks from diagnosis (GP) (%)	52.8	54.3	56.4	61.1	58.3	63.9	65.0	65.9

For peer review only

Patients <56 years that had surgery within 2 days from diagnosis (GP) (%)	1.4	0.8	0.3	0.7	0.3	0.5	0.3	1.1
Patients <56 years that had surgery between 2 days and 12 weeks from diagnosis (GP) (%)	30.7	28.7	26.8	25.5	24.6	24.3	20.3	20.0
Patients <age 56 years that had surgery after 12 weeks and before 20 weeks from diagnosis (GP) (%)	18.9	18.8	22.0	15.1	20.3	17.4	19.0	17.2
Patients <age 56 years that had surgery after 20 weeks from diagnosis (GP) (%)	49.0	51.7	50.8	58.7	54.8	57.7	60.4	61.6
Waiting time for surgery (days)	31	29	32	34	36	39	38	43

[a] number of surgical treated patients that could be linked between the NIVEL and NZA database [b] General Practitioner

For peer review only

For peer review only - http://bmjopen.bmj.com/site/about/guidelines.xhtml

For peer review only

For peer review only - http://bmjopen.bmj.com/site/about/guidelines.xhtml

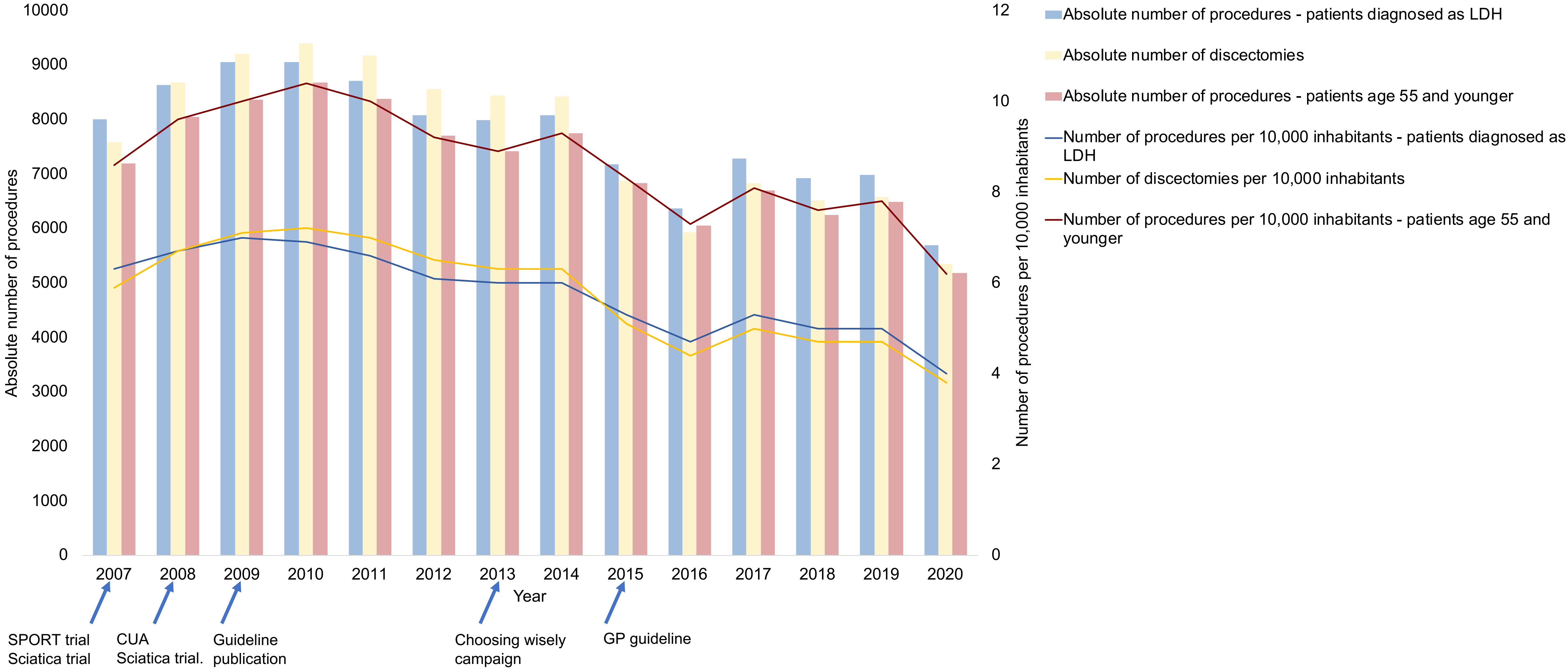
10000
9000
8000
7000
6000
5000
4000
3000
2000
1000
0
Absolute number of procedures
12
10
8
6
4
2
0
Number of procedures per 10,000 inhabitants
2007
2008
2009
2010
2011
2012
2013
2014
2015
2016
2017
2018
2019
2020
Year
SPORT trial Sciatica trial
CUA Sciatica trial.
Guideline publication
Choosing wisely campaign
GP guideline
Absolute number of procedures - patients diagnosed as LDH
Absolute number of discectomies
Absolute number of procedures - patients age 55 and younger
Number of procedures per 10,000 inhabitants - patients diagnosed as LDH
Number of discectomies per 10,000 inhabitants
Number of procedures per 10,000 inhabitants - patients age 55 and younger

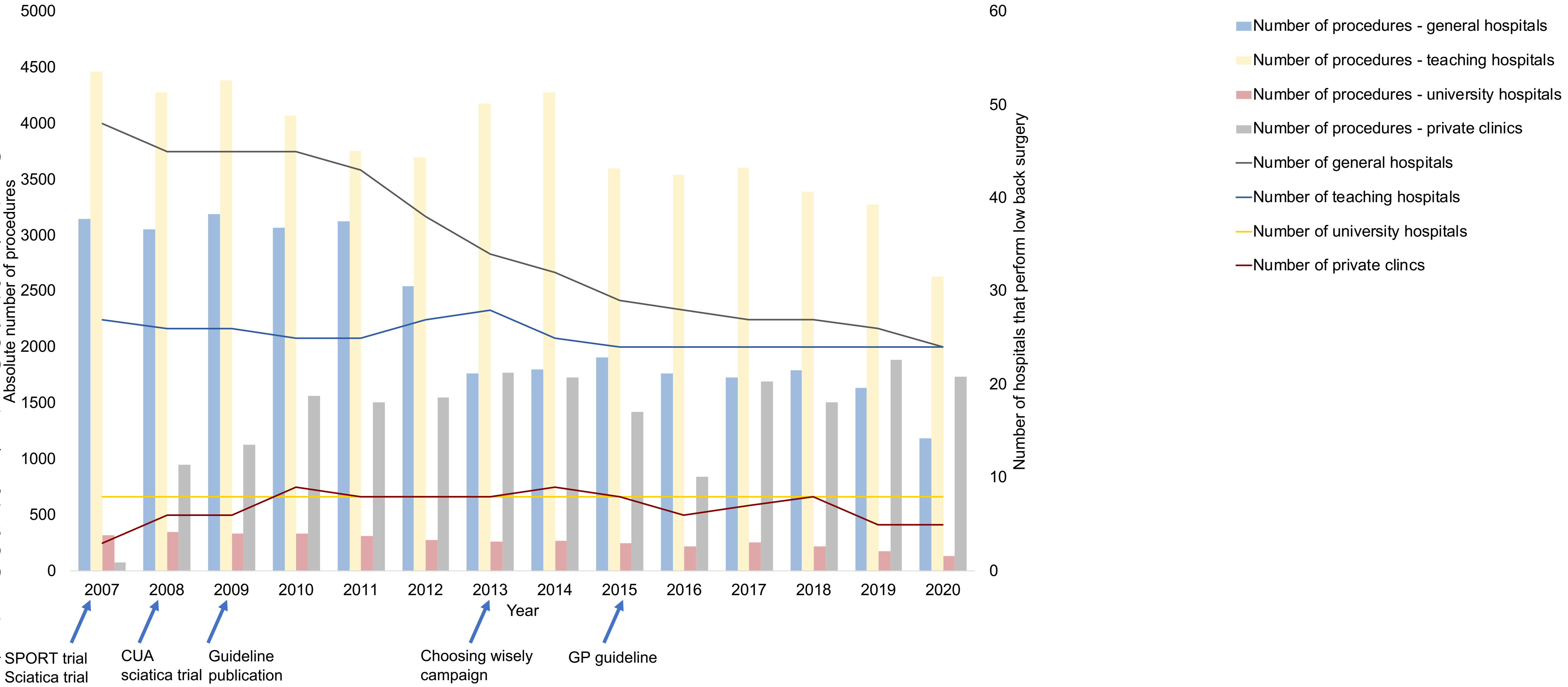
5000
4500
4000
3500
3000
2500
2000
1500
1000
500
0
Absolute number of procedures
60
50
40
30
20
10
0
Number of hospitals that perform low back surgery
2007
2008
2009
2010
2011
2012
2013
2014
2015
2016
2017
2018
2019
2020
Year
Number of procedures - general hospitals
Number of procedures - teaching hospitals
Number of procedures - university hospitals
Number of procedures - private clinics
Number of general hospitals
Number of teaching hospitals
Number of university hospitals
Number of private clincs
SPORT trial
Sciatica trial
CUA
sciatica trial
Guideline
publication
Choosing wisely
campaign
GP guideline

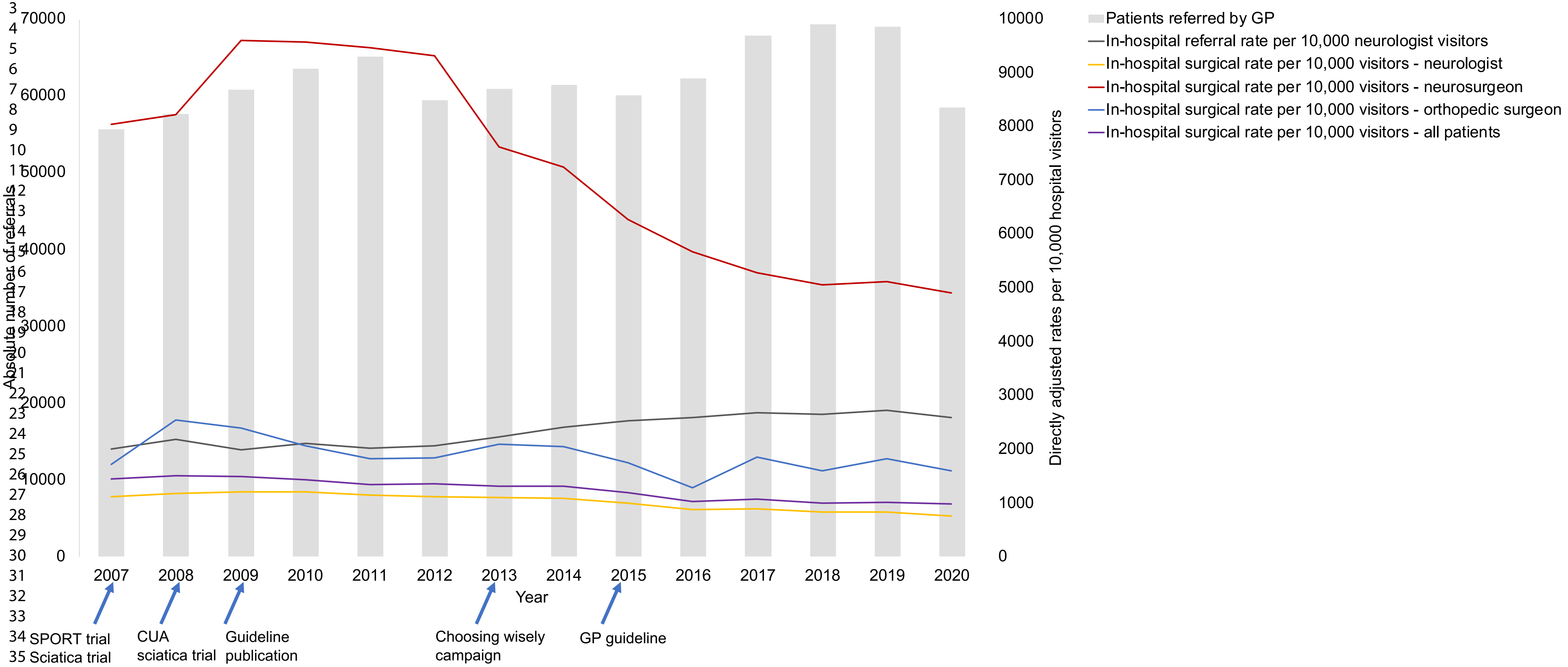
70000
60000
50000
40000
30000
20000
10000
0
Absolute number of referrals
10000
9000
8000
7000
6000
5000
4000
3000
2000
1000
0
Directly adjusted rates per 10,000 hospital visitors
2007
2008
2009
2010
2011
2012
2013
2014
2015
2016
2017
2018
2019
2020
Year
Patients referred by GP
In-hospital referral rate per 10,000 neurologist visitors
In-hospital surgical rate per 10,000 visitors - neurologist
In-hospital surgical rate per 10,000 visitors - neurosurgeon
In-hospital surgical rate per 10,000 visitors - orthopedic surgeon
In-hospital surgical rate per 10,000 visitors - all patients
SPORT trial
Sciatica trial
CUA sciatica trial
Guideline publication
Choosing wisely campaign
GP guideline

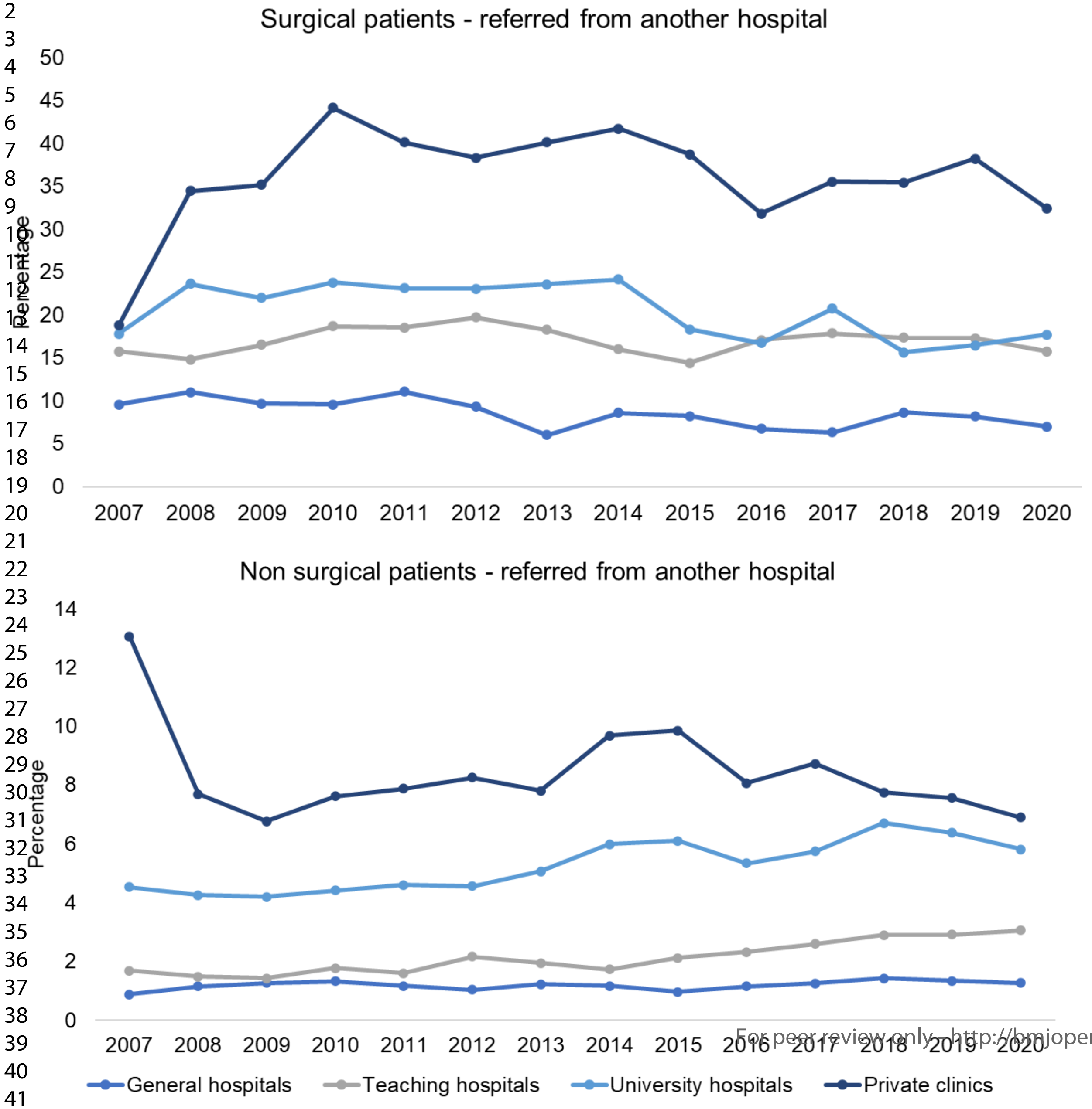
Surgical patients - referred from another hospital
Percentage
50
45
40
35
30
25
20
15
10
5
0
2007 2008 2009 2010 2011 2012 2013 2014 2015 2016 2017 2018 2019 2020
Non surgical patients - referred from another hospital
Percentage
14
12
10
8
6
4
2
0
2007 2008 2009 2010 2011 2012 2013 2014 2015 2016 2017 2018 2019 2020
General hospitals
Teaching hospitals
University hospitals
Private clinics

APPENDIX

Table S1. Used diagnosis treatment codes

Diagnosis treatment codes	Description
Included	
0330-12-00-1203	Pseudo radicular syndrome
0330-12-00-1204	Radicular syndrome
0330-12-00-1211<br>0305-13-00-1350	Spinal stenosis
0305-13-00-1360	Hernia Nuclei Pulposis
0305-13-00-1370	Spondylolisthesis
0308-02-25-2501<br>0308-02-25-2550<br>0308-02-25-2565<br>0308-02-25-2555<br>0308-02-25-2505<br>0308-02-25-2510<br>0308-02-25-2515	Degenerative disc disease
0330-12-00-1231<br>0305-13-00-1330<br>0305-13-00-1340	Low backpain
Excluded	
0330-12-00-1201<br>0330-12-00-1202<br>0330-05-00-0543<br>0305-12-00-1202<br>0305-12-00-1220<br>0305-12-00-1240<br>0308-02-25-2525<br>0308-02-25-2527<br>0308-02-25-2530<br>0308-02-25-2535	Cervical diagnosis codes
0330-02-00-0232<br>0330-02-00-0233<br>0330-02-00-0221<br>0330-02-00-0222<br>0330-02-00-0223<br>0308-02-21-2105<br>0308-02-21-2110	Tumors, malignancies of the spine

For peer review only

0308-02-21-2115	
0308-02-24-2405<br>0308-02-24-2411<br>0305-13-00-1301<br>0305-13-00-1302	Infectious spine disease
0305-13-00-1396<br>0308-02-23-2305<br>0308-02-23-2311	Spine trauma, fractures
0305-13-00-1397<br>0305-13-00-1380<br>0305-13-00-1381<br>0305-13-00-1383<br>0305-13-00-1391	Congenital disc disease, kyphosis, scoliosis

Table S2. Used care product codes

**Care product code**	**Description**
Included	
38437<br>38444<br>38438<br>38467	Discectomy
30327<br>30329	Laminectomy
38458<br>38459<br>38460<br>38464<br>38468<br>38469	Fusion

Table S3. Inclusion criteria for the LDH subgroups

**Lumbar disc herniation diagnosis group**	
Included diagnosis codes	
0305-13-00-1360	Hernia Nuclei Pulposis
Excluded diagnosis codes	
0330-12-00-1201<br>0330-12-00-1202<br>0330-05-00-0543<br>0305-12-00-1202	Cervical diagnosis codes

For peer review only

0305-12-00-1220<br>0305-12-00-1240<br>0308-02-25-2525<br>0308-02-25-2527<br>0308-02-25-2530<br>0308-02-25-2535	
0330-02-00-0232<br>0330-02-00-0233<br>0330-02-00-0221<br>0330-02-00-0222<br>0330-02-00-0223<br>0308-02-21-2105<br>0308-02-21-2110<br>0308-02-21-2115	Tumors, malignancies of the spine
0308-02-24-2405<br>0308-02-24-2411<br>0305-13-00-1301<br>0305-13-00-1302	Infectious spine disease
0305-13-00-1396<br>0308-02-23-2305<br>0308-02-23-2311	Spine trauma, fractures
0305-13-00-1397<br>0305-13-00-1380<br>0305-13-00-1381<br>0305-13-00-1383<br>0305-13-00-1391	Congenital disc disease, kyphosis, scoliosis
**Included care product codes**	
38437<br>38444<br>38438<br>38467	Discectomy
30327<br>30329	Laminectomy
38458<br>38459<br>38460<br>38464<br>38468<br>38469	Fusion

**Discectomy group**	
Included diagnosis codes	

For peer review only

0330-12-00-1203	Pseudo radicular syndrome
0330-12-00-1204	Radicular syndrome
0330-12-00-1211<br>0305-13-00-1350	Spinal stenosis
0305-13-00-1360	Hernia Nuclei Pulposis
0305-13-00-1370	Spondylolisthesis
0308-02-25-2501<br>0308-02-25-2550<br>0308-02-25-2565<br>0308-02-25-2555<br>0308-02-25-2505<br>0308-02-25-2510<br>0308-02-25-2515	Degenerative disc disease
0330-12-00-1231<br>0305-13-00-1330<br>0305-13-00-1340	Low backpain
Excluded diagnosis codes	
0330-12-00-1201<br>0330-12-00-1202<br>0330-05-00-0543<br>0305-12-00-1202<br>0305-12-00-1220<br>0305-12-00-1240<br>0308-02-25-2525<br>0308-02-25-2527<br>0308-02-25-2530<br>0308-02-25-2535	Cervical diagnosis codes
0330-02-00-0232<br>0330-02-00-0233<br>0330-02-00-0221<br>0330-02-00-0222<br>0330-02-00-0223<br>0308-02-21-2105<br>0308-02-21-2110<br>0308-02-21-2115	Tumors, malignancies of the spine
0308-02-24-2405<br>0308-02-24-2411<br>0305-13-00-1301<br>0305-13-00-1302	Infectious spine disease
0305-13-00-1396<br>0308-02-23-2305<br>0308-02-23-2311	Spine trauma, fractures

For peer review only

0305-13-00-1397<br>0305-13-00-1380<br>0305-13-00-1381<br>0305-13-00-1383<br>0305-13-00-1391	Congenital disc disease, kyphosis, scoliosis
**Included care product codes**	
38437<br>38444<br>38438<br>38467	Discectomy

**Age (<56 years) group**	
Included diagnosis codes	
0330-12-00-1203	Pseudo radicular syndrome
0330-12-00-1204	Radicular syndrome
0330-12-00-1211<br>0305-13-00-1350	Spinal stenosis
0305-13-00-1360	Hernia Nuclei Pulposis
0305-13-00-1370	Spondylolisthesis
0308-02-25-2501<br>0308-02-25-2550<br>0308-02-25-2565<br>0308-02-25-2555<br>0308-02-25-2505<br>0308-02-25-2510<br>0308-02-25-2515	Degenerative disc disease
0330-12-00-1231<br>0305-13-00-1330<br>0305-13-00-1340	Low backpain
Excluded diagnosis codes	
0330-12-00-1201<br>0330-12-00-1202<br>0330-05-00-0543<br>0305-12-00-1202<br>0305-12-00-1220<br>0305-12-00-1240<br>0308-02-25-2525<br>0308-02-25-2527<br>0308-02-25-2530<br>0308-02-25-2535	Cervical diagnosis codes
0330-02-00-0232	Tumors, malignancies of the spine

For peer review only

0330-02-00-0233<br>0330-02-00-0221<br>0330-02-00-0222<br>0330-02-00-0223<br>0308-02-21-2105<br>0308-02-21-2110<br>0308-02-21-2115	
0308-02-24-2405<br>0308-02-24-2411<br>0305-13-00-1301<br>0305-13-00-1302	Infectious spine disease
0305-13-00-1396<br>0308-02-23-2305<br>0308-02-23-2311	Spine trauma, fractures
0305-13-00-1397<br>0305-13-00-1380<br>0305-13-00-1381<br>0305-13-00-1383<br>0305-13-00-1391	Congenital disc disease, kyphosis, scoliosis
**Included care product codes**	
38437<br>38444<br>38438<br>38467	Discectomy
30327<br>30329	Laminectomy
38458<br>38459<br>38460<br>38464<br>38468<br>38469	Fusion

For peer review only

Table S4. Full table of surgical trends between 2007 and 2020 for hospital visitors with a likely diagnosis of lumbar disc herniation

	2007	2008	2009	2010	2011	2012	2013	2014	2015	2016	2017	2018	2019	2020
**Patients with a registered diagnosis of LDH[a]**														
Number of procedures	8009	8638	9048	9046	8708	8077	7987	8085	7183	6372	7290	6917	6983	5693
Per 10,000 inhabitants	6.3	6.7	7.0	6.9	6.6	6.1	6.0	6.0	5.3	4.7	5.3	5.0	5.0	4.0
Per 10,000 hospital visitors	1441	1500	1489	1425	1339	1361	1312	1317	1196	1024	1076	998	1012	974
Male	4278	4675	4822	4827	4645	4230	4177	4194	3802	3156	3737	3460	3567	2845
Female	3731	3963	4226	4219	4063	3847	3810	3891	3381	3216	3553	3457	3416	2848
Neurosurgery	6941	6905	7250	7629	7431	7004	6928	6982	6411	5935	6381	6286	6208	5211
Orthopedic surgery	1282	2023	2078	1735	1602	1364	1383	1422	1063	652	1216	932	1088	773
Teaching hospitals	4463	4279	4389	4074	3757	3698	4177	4281	3598	3542	3607	3394	3277	2634
General hospitals	3150	3059	3193	3071	3127	2550	1769	1801	1913	1770	1732	1794	1640	1187
Private clinics	75	950	1132	1565	1509	1554	1775	1734	1421	840	1698	1511	1888	1741
University hospitals	321	350	334	336	315	275	266	269	251	220	253	218	178	131
**Patients who had a discectomy**														
Number of procedures	7586	8673	9204	9403	9172	8561	8443	8425	6872	5931	6828	6522	6585	5344
Per 10,000 inhabitants	5.9	6.7	7.1	7.2	7.0	6.5	6.3	6.3	5.1	4.4	5.0	4.7	4.7	3.8
Per 10,000 hospital visitors	656	702	719	687	658	689	652	625	524	436	472	447	442	427
Male	4084	4714	4898	5021	4944	4504	4438	4379	3676	2951	3489	3259	3348	2645
Female	3502	3959	4306	4382	4228	4057	4005	4046	3196	2980	3339	3263	3237	2699
Neurosurgery	6727	6973	7380	7909	7832	7507	7463	7445	6271	5605	6153	6079	5992	5015
Orthopedic surgery	1083	2040	2167	1888	1736	1445	1403	1400	929	593	1062	806	1023	701
Teaching hospitals	4261	4263	4493	4255	3939	3967	4482	4508	3634	3530	3634	3392	3276	2616
General hospitals	2963	3079	3210	3151	3353	2711	1832	1851	1683	1407	1447	1616	1510	1055
Private clinics	8	960	1152	1634	1561	1589	1822	1775	1289	750	1495	1305	1604	1557
University hospitals	354	371	349	363	319	294	307	291	266	244	252	209	195	116
**Patients age < 56 years**														
Number of procedures	7192	8052	8363	8683	8383	7708	7414	7742	6827	6052	6691	6243	6484	5184
Per 10,000 inhabitants	8.6	9.6	10.0	10.4	10.0	9.2	8.9	9.3	8.3	7.3	8.1	7.6	7.8	6.2
Per 10,000 hospital visitors	1103	1187	1204	1181	1139	1200	1139	1183	1107	971	1028	969	1016	977
Male	3881	4386	4489	4638	4486	4060	3876	4011	3583	3019	3426	3105	3274	2540
Female	3311	3666	3874	4045	3897	3648	3538	3731	3244	3033	3265	3138	3210	2644
Neurosurgery	6198	6376	6632	7152	7054	6619	6371	6633	6085	5575	5807	5625	5694	4732
Orthopedic surgery	1230	2010	2059	1927	1711	1416	1425	1509	1093	753	1242	922	1158	754
Teaching hospitals	4026	3962	4098	3989	3651	3589	3868	4091	3434	3435	3362	3141	3150	2401
General hospitals	2703	2786	2846	2802	2927	2305	1589	1672	1759	1536	1538	1521	1443	1077
Private clinics	58	864	994	1467	1415	1404	1599	1582	1290	758	1473	1304	1625	1509
University hospitals	405	440	425	425	390	410	358	397	344	323	318	277	266	197

For peer review only

Table S5. Full table of time between diagnosis, first hospital-visit and surgery between 2012 and 2020 (Nivel and NZA database)

	2012 (n=697)	2013 (n=958)	2014 (n=1174)	2015 (n=986)	2016 (n=804)	2017 (n=947)	2018 (n=902)	2019 (n=1031)	2020 (n=744)
Days between first GP[b] visit and first hospital visit (average)	158	155	163	179	180	174	184	169	193
**First GP visit to surgery**									
Days between first GP visit and surgery, median (25th-75th)	154 (80 – 361)	154 (77 – 328)	166 (87 – 351)	183 (91 – 361)	174 (92 – 371)	187 (103 – 354)	202 (104 – 386)	196 (104 – 383)	239 (137 – 451)
Days between first GP visit and surgery, 5th-95th	15 - 695	22 - 756	29 - 795	34 - 803	26 - 822	35 - 824	35 - 857	36 - 803	43 - 840
Days between first GP visit and surgery for patients <56 years, median (25th-75th)	137 (74-332)	147 (71-310)	142 (79-280)	167 (82-341)	161 (86-337)	160 (85-301)	172 (91-346)	183 (91-347)	230 (111-415)
**Time from first hospital visit to surgery**									
Days between first hospital visit and surgery, median (25th-75th)	51 (25-91)	51 (25-91)	56 (26-99)	62 (32-106)	59 (29-96)	70 (35-118)	67 (35-119)	70 (35-125)	80 (40-141)
Days between first hospital visit and surgery, 5th-95th	2 - 223	6 - 207	7 - 223	7 - 201	8 - 212	8 - 223	7 - 223	7 - 239	8 – 264
Days between first hospital visit and surgery for patients <56 years, median (25th-75th)	40 (21-78)	45 (21-81)	45 (21-81)	48 (23-86)	46 (22-78)	63 (31-105)	56 (23-108)	59 (28-110)	63 (31-118)
**Following the guideline?**									
Patients that had surgery within 2 days from diagnosis (GP) (%)	0.7	0.8	0.5	0.5	0.5	0.4	0.3	0.8	0.4
Patients that had surgery between 2 days and 12 weeks from diagnosis (GP) (%)	26.2	26.3	23.2	22.1	21.8	18.3	17.3	17.6	12.4
Patients that had surgery after 12 weeks and before 20 weeks from diagnosis (GP) (%)	20.2	18.6	19.7	16.3	19.4	17.3	17.3	15.7	13.0
Patients that had surgery after 20 weeks from diagnosis (GP) (%)	52.8	54.3	56.4	61.1	58.3	63.9	65.0	65.9	74.2
Patients <56 years that had surgery within 2 days from diagnosis (GP) (%)	1.4	0.8	0.3	0.7	0.3	0.5	0.3	1.1	0.0
Patients <56 years that had surgery between 2 days and 12 weeks from diagnosis (GP) (%)	30.7	28.7	26.8	25.5	24.6	24.3	20.3	20.0	17.2
Patients <age 56 years that had surgery after 12 weeks and before 20 weeks from diagnosis (GP) (%)	18.9	18.8	22.0	15.1	20.3	17.4	19.0	17.2	14.3
Patients <age 56 years that had surgery after 20 weeks from diagnosis (GP) (%)	49.0	51.7	50.8	58.7	54.8	57.7	60.4	61.6	68.5
Waiting time for surgery (days)	31	29	32	34	36	39	38	43	44

[a] number of surgical treated patients that could be linked between the NIVEL and NZA database [b] General Practitioner

For peer review only

	2007	2008	2009	2010	2011	2012	2013	2014	2015	2016	2017	2018	2019	2020
**Patients with a registered diagnosis of LDH[a]**														
Hospital visitors	55581	57597	60777	63493	65023	59365	60883	61396	60040	62217	67771	69296	68997	58442
Per 10,000 inhabitants	43	45	47	49	49	45	46	46	45	46	50	50	50	42
Male	28528	29674	31064	32271	32624	29446	29838	30190	29260	30104	32362	32836	32934	27733
Female	27053	27923	29713	31222	32399	29919	31045	31206	30780	32113	35409	36460	36063	30709
Neurology	48447	50803	53529	56499	57929	52870	54380	54382	53327	56397	60410	62255	62047	52314
Neurosurgery	8643	8409	7560	7970	7859	7522	9099	9644	10240	10487	12084	12444	12145	10648
Orthopedic surgery	7490	7944	8678	8437	8764	7435	6605	6954	6071	5097	6595	5840	5976	4843
Referred by neurologist to surgical department	7155	7526	7005	7506	7346	6896	7541	7790	7991	8180	9521	9457	9398	7838
Teaching hospitals	23509	23736	25298	25115	25964	24972	30872	30979	30855	31844	33391	34414	33453	28581
General hospitals	27714	26461	27189	28910	29079	24980	21083	22322	22026	22598	22525	22241	22224	18534
Private clinics	1774	4806	5797	6912	7461	7005	6518	5547	4963	5334	9394	10315	11097	9301
University hospitals	2584	2594	2493	2556	2519	2408	2410	2548	2196	2441	2461	2326	2223	2026
Mean per hospital														
Teaching hospitals	871	913	973	966	999	892	1065	1192	1234	1274	1336	1377	1338	1143
General hospitals	462	448	486	535	539	480	449	507	551	595	644	635	635	545
Private clinics	197	267	290	288	298	280	251	264	216	254	447	491	555	443
University hospitals	323	324	312	320	315	301	301	319	275	305	308	291	278	253
**Patients age < 56 years**														
Hospital visitors	65185	67861	69449	73503	73569	64257	65093	65463	61695	62308	65056	64399	63808	53077
Per 10,000 inhabitants	78	81	83	88	88	77	78	79	75	75	79	78	77	64
Male	32213	33336	34010	35468	35050	30228	30358	30501	28412	28623	29788	29004	28938	23817
Female	32972	34525	35439	38035	38519	34029	34735	34962	33283	33685	35268	35395	34870	29260
Neurology	48448	51585	54337	57659	57969	50346	50583	50188	47716	48789	50783	50658	11571	40686
Neurosurgery	8902	8747	7131	7665	7615	7342	9499	10158	10629	10300	11270	11415	49746	10581
Orthopedic surgery	15865	16559	16607	17370	17256	14589	13514	14121	11813	11584	12328	11343	11537	9144
Referred by neurologist to surgical department	5164	5562	5205	5574	5507	5021	5410	5488	5414	5281	6071	5928	5763	4656
Teaching hospitals	27871	28569	29173	30248	30182	27595	33030	32593	31141	31510	32770	32911	31988	25935
General	31533	30973	31641	33444	33092	26749	21881	22868	21473	21901	21370	20161	19668	15801
University hospitals	3969	4037	3655	3541	3437	3224	3153	3200	2873	3218	2897	2548	2480	2101
Private clinics	1812	4282	4980	6270	6858	6689	7029	6802	6208	5679	8019	8779	9672	9240
Mean per hospital														
Teaching hospital	214	284	311	338	337	317	332	372	348	348	413	425	467	422
General	513	525	565	627	620	527	482	523	533	574	628	589	586	491
University hospital	1072	1106	1133	1051	1020	884	1070	1226	1300	1314	1262	1225	1347	1006
Private clinics	496	505	457	443	430	403	394	400	359	402	362	319	310	263

[a] Lumbar Disc Herniation

Table S6. Trends between 2007 and 2020 for hospital visitors with a likely diagnosis of lumbar disc herniation

For peer review only

For peer review only

STROBE Statement—Checklist of items that should be included in reports of ***cross-sectional studies***

	Item No	Recommendation
**Title and abstract**	1	(*a*) Indicate the study's design with a commonly used term in the title or the abstract (p1, l1)
		(*b*) Provide in the abstract an informative and balanced summary of what was done and what was found (p2)
**Introduction**		
Background/rationale	2	Explain the scientific background and rationale for the investigation being reported (p3)
Objectives	3	State specific objectives, including any prespecified hypotheses (p3, p4; l89-94)
**Methods**		
Study design	4	Present key elements of study design early in the paper (p4; l96)
Setting	5	Describe the setting, locations, and relevant dates, including periods of recruitment, exposure, follow-up, and data collection (p4; section data sources)
Participants	6	(*a*) Give the eligibility criteria, and the sources and methods of selection of participants (p4; section population)
Variables	7	Clearly define all outcomes, exposures, predictors, potential confounders, and effect modifiers. Give diagnostic criteria, if applicable (p5; section analyses)
Data sources/ measurement	8*	For each variable of interest, give sources of data and details of methods of assessment (measurement). Describe comparability of assessment methods if there is more than one group (p5, p6; section data collection, analyses, costa analysis and analytic approach)
Bias	9	Describe any efforts to address potential sources of bias (p5, p6; section analyses, cost analysis and analytic approach)
Study size	10	Explain how the study size was arrived at (n.a. (population-based))
Quantitative variables	11	Explain how quantitative variables were handled in the analyses. If applicable, describe which groupings were chosen and why (p6; section analytic approach)
Statistical methods	12	(*a*) Describe all statistical methods, including those used to control for confounding (p6; analytic approach)
		(*b*) Describe any methods used to examine subgroups and interactions (n.a.)
		(*c*) Explain how missing data were addressed (we had no missing data due to the administrative study design)
		(*d*) If applicable, describe analytical methods taking account of sampling strategy (n.a.)
		(*e*) Describe any sensitivity analyses (n.a.)
**Results**		
Participants	13*	(a) Report numbers of individuals at each stage of study—eg numbers potentially eligible, examined for eligibility, confirmed eligible, included in the study, completing follow-up, and analysed (p6; section study population)
		(b) Give reasons for non-participation at each stage (n.a.)
		(c) Consider use of a flow diagram (n.a.)
Descriptive data	14*	(a) Give characteristics of study participants (eg demographic, clinical, social) and information on exposures and potential confounders (p6; section study population)
		(b) Indicate number of participants with missing data for each variable of interest (n.a.)
Outcome data	15*	Report numbers of outcome events or summary measures (p7; l179 – p8 l211)

For peer review only

Main results	16	(*a*) Give unadjusted estimates and, if applicable, confounder-adjusted estimates and their precision (eg, 95% confidence interval). Make clear which confounders were adjusted for and why they were included (n.a. due to the study design)
		(*b*) Report category boundaries when continuous variables were categorized (n.a.)
		(*c*) If relevant, consider translating estimates of relative risk into absolute risk for a meaningful time period (not relevant)
Other analyses	17	Report other analyses done—eg analyses of subgroups and interactions, and sensitivity analyses (p8; section cost analysis)
**Discussion**		
Key results	18	Summarise key results with reference to study objectives (p8; l218-l224)
Limitations	19	Discuss limitations of the study, taking into account sources of potential bias or imprecision. Discuss both direction and magnitude of any potential bias (p10; l283-l294)
Interpretation	20	Give a cautious overall interpretation of results considering objectives, limitations, multiplicity of analyses, results from similar studies, and other relevant evidence (p11; section conclusion)
Generalisability	21	Discuss the generalisability (external validity) of the study results (not important due to the population-based design (all inhabitants are included)
**Other information**		
Funding	22	Give the source of funding and the role of the funders for the present study and, if applicable, for the original study on which the present article is based (p1)

*Give information separately for exposed and unexposed groups.

Note: An Explanation and Elaboration article discusses each checklist item and gives methodological background and published examples of transparent reporting. The STROBE checklist is best used in conjunction with this article (freely available on the Web sites of PLoS Medicine at http://www.plosmedicine.org/, Annals of Internal Medicine at http://www.annals.org/, and Epidemiology at http://www.epidem.com/). Information on the STROBE Initiative is available at www.strobe-statement.org.

For peer review only

BMJ Open

the impact of evidence-based guidelines on healthcare utilization and costs for disc related sciatica in the Netherlands: a population-based, cross-sectional study

Journal:	*BMJ Open*
Manuscript ID	bmjopen-2023-078459.R2
Article Type:	Original research
Date Submitted by the Author:	08-Jan-2024
Complete List of Authors:	van Munster, Juliëtte; Leiden University, Department of Otorhinolaryngology and Head and Neck Surgery<br>Noordenbos, Mark; Dutch Healthcare Authority<br>Halperin, I.J.Y.; Leiden University, Department of Otorhinolaryngology and Head and Neck Surgery<br>van den Hout, Wilbert; Leiden University Medical Center, Medical Decision Making<br>van Benthem, Peter Paul; Leiden University Medical Center, Otorhinolaryngology - Head and Neck Surgery<br>Seinen, Ingrid; Dutch Healthcare Authority<br>Moojen, Wouter; Medical Center Haaglanden, Neurosurgery; HAGA hospital, Neurosurgery<br>Peul, Wilco; Leiden University Medical Center, Neurosurgery; Medical Center The Hague, Neurosurgery
**Primary Subject Heading**:	Evidence based practice
Secondary Subject Heading:	Epidemiology, General practice / Family practice, Neurology, Surgery
Keywords:	Clinical Decision-Making, Protocols & guidelines < HEALTH SERVICES ADMINISTRATION & MANAGEMENT, Neuromuscular disease < NEUROLOGY

SCHOLARONE™
Manuscripts

I, the Submitting Author has the right to grant and does grant on behalf of all authors of the Work (as defined in the below author licence), an exclusive licence and/or a non-exclusive licence for contributions from authors who are: i) UK Crown employees; ii) where BMJ has agreed a CC-BY licence shall apply, and/or iii) in accordance with the terms applicable for US Federal Government officers or employees acting as part of their official duties; on a worldwide, perpetual, irrevocable, royalty-free basis to BMJ Publishing Group Ltd ("BMJ") its licensees and where the relevant Journal is co-owned by BMJ to the co-owners of the Journal, to publish the Work in this journal and any other BMJ products and to exploit all rights, as set out in our licence.

The Submitting Author accepts and understands that any supply made under these terms is made by BMJ to the Submitting Author unless you are acting as an employee on behalf of your employer or a postgraduate student of an affiliated institution which is paying any applicable article publishing charge ("APC") for Open Access articles. Where the Submitting Author wishes to make the Work available on an Open Access basis (and intends to pay the relevant APC), the terms of reuse of such Open Access shall be governed by a Creative Commons licence – details of these licences and which Creative Commons licence will apply to this Work are set out in our licence referred to above.

Other than as permitted in any relevant BMJ Author's Self Archiving Policies, I confirm this Work has not been accepted for publication elsewhere, is not being considered for publication elsewhere and does not duplicate material already published. I confirm all authors consent to publication of this Work and authorise the granting of this licence.

For peer review only

Title: the impact of evidence-based guidelines on healthcare utilization and costs for disc related sciatica in the Netherlands: a population-based, cross-sectional study

Authors: Juliëtte J.C.M. van Munster[1,2] MD, Mark W. Noordenbos, PhD[3], I.J.Y. Halperin[1,2] MD, Wilbert B. van den Hout[4] PhD, Peter Paul G. van Benthem[2] MD, PhD, Ingrid Seinen, MSc[3], Wouter Moojen[1] MD, PhD, Wilco C. Peul[1,5] MD, PhD

[1] University Neurosurgical Centre Holland (UNCH), LUMC | HMC | HAGA, Leiden & The Hague, the Netherlands

[2] Department of Otorhinolaryngology and Head and Neck Surgery, Leiden University Medical Center, Leiden University, the Netherlands

[3] Dutch Healthcare Authority, Utrecht, the Netherlands.

[4] Department of Biomedical Data Sciences, Leiden University Medical Center, Leiden, the Netherlands

[5] Netherlands Organization for Health Research and Innovation (ZonMW)

Corresponding author:

Juliëtte van Munster, Dept. of Neurosurgery, Leiden University Medical Centre, P.O. Box 9600, 2300 RC Leiden, the Netherlands, Phone: +3171 526 5870, e-mail: j.j.c.m.van_munster@lumc.nl

Conflict of interest: Prof. dr. Peul: Boardmember ZonMW, former Chair Netherlands Choosing Wisely FMS committee

Prof. dr. van Benthem: Former president of the Federation Medical Specialists (FMS)

Dr. W. Moojen is a Board member of the Dutch Neurosurgical Society.

Supported by a grant from: the Netherlands Organization for Health Research and Development (Citrienfonds), grant number: 839205002. The funding organization had no role in the present study.

For peer review only - http://bmjopen.bmj.com/site/about/guidelines.xhtml

Prof. dr. Peul and dr. W. van den Hout were involved in the design and execution of the sciatica trial. Furthermore, prof dr. Peul was also involved in the Guideline construction and leader of the Netherlands Choosing Wisely campaign on behalf of FMS and ZonMW.

Ethical approval: our study was approved by the Medical Ethical Committee of Leiden and the Hague (metc-ldd@lumc.nl; N20.075)

Word count: 4194

Target journal: BMJ Open

For peer review only

ABSTRACT

Objective: The aim of this study was to assess the impact of high-quality evidence supporting surgical treatment of lumbar disc herniation (LDH) on healthcare practice in the Netherlands by examining changes in healthcare utilization, including the timing of surgery, and healthcare costs for LDH patients.

Design: A retrospective, cross-sectional study was performed utilizing population-based, longitudinal data obtained from the Dutch Healthcare Authority (2007-2020) and NIVEL's primary care (2012-2020) administrative databases.

Setting: The study was conducted within the healthcare system of the Netherlands.

Participants: We included adults (≥18 years) who visited a Dutch hospital or a General Practitioner for lumbar degenerative disc disease. LDH patients were identified based on registered diagnosis code, type of surgery (discectomy), and age (<56 years).

Main outcome measures: The primary outcome measure was the difference in the annual number of LDH procedures following the publication of evidence-based guidelines in 2009 (comparing the periods 2007-2009 to 2017-2019). Secondary outcome measures focused on the timing of surgery and associated healthcare costs. To validate the outcomes, secondary outcomes also include number of discectomies and number of procedures in the younger age group (discectomies, laminectomies, and fusion surgery).

Results: Number of patients suffering from LDH increased from 55.581 to 68.997 (+24%) between 2007 and 2019. A decrease was observed in annual number of LDH procedures (-18%), in number of discectomies (-22%), and in number of procedures for patients age <56 years (-18%). This resulted in lower

healthcare costs by €12 million annually. In 2012, 31% of all patients <56 years had surgery before 12 weeks from diagnosis at the GP, whereas 20% did in 2019.

Conclusions: Healthcare utilization for LDH changed tremendously in the Netherlands between 2007 and 2020, and seemed to be associated with the publication and implementation of evidence-based guidelines. The observed decrease in the number of procedures has been accompanied by a corresponding reduction in healthcare costs. These findings underscore the importance of adhering to evidence-based guidelines to optimize the management of LDH patients.

Strengths and limitations of this study

- A strength of this study is the nationwide coverage of the database.
- Another strength is the fact that sciatica patients were identified by analyzing three patient identifiers (diagnosis code, age, and type of surgery) to validate our findings and minimize administrative errors.
- Furthermore, a strength of this study is the fact that policy makers and healthcare professionals performed this study together, supporting the independence of the study results.

A limitation of the study is that causation between guideline publication and changes in healthcare practice cannot be proven due to lack of data availability from before 2007.

INTRODUCTION

Lumbosacral Radicular Syndrome (LRS), or sciatica due to a lumbar disc herniation (LDH) is common in everyday healthcare practices with a substantial burden for healthcare and society(1). Intolerable pain and cauda equina syndrome are definite indications for surgery, but most patients suffering from

For peer review only

sciatica recover without the need of surgery within three months from onset(2), after which period the natural course is less favorable with a longer wait-and-see strategy(3).

In 1999, in response to a provoking practice variation publication(4), the Health Council of the Netherlands published an evidence-based report on this topic and concluded that there was lack of evidence on effectiveness and timing of surgery, but also serious signs of overtreatment in the Netherlands compared to the United Kingdom (UK)(5). In response, a Randomized Controlled Trial was designed and performed on this topic in the Netherlands (Sciatica trial; 2007)(6). Comparing the Netherlands with UK guidelines in timing of discectomy, patients suffering from severe sciatica for 6 to 12 weeks were randomly assigned to a timing of early surgery or to prolonged conservative treatment up to 6 months with possible delayed surgery. Around the same time, the SPORT trial (Weinstein et al.; 2006) was performed in the United States of America (USA)(7). Both trials showed no more differences in pain scores beyond one year, but with faster initial pain relief and better quality-adjusted life years after early surgery compared to conservative treatment(6, 8). As a result, early surgery was a cost-effective option that need not to be withheld for economic reasons, although it was associated with higher healthcare costs(9).

These trials led to the development of the first multidisciplinary, evidence-based guideline for LRS in the Netherlands in 2009(10) and several other countries(11, 12). An initial conservative treatment strategy is recommended in the first three months after onset of sciatica (in absence of progressive neurological deficit and cauda equina syndrome), whereas a shared decision-making treatment strategy between patient and physician is advised after three months. Guideline recommendations were incorporated in 'Wise Choices' in the Dutch version of the North American initiated Choosing Wisely campaign in 2014(13, 14). In these 'Wise Choices', it is also advised to proceed to surgery within 9 months after disease onset. These recommendations were integrated in the General Practitioner (GP) guideline in 2015(15).

It was decided by the original researchers in collaboration with the Federation Medical Specialists (FMS) and the Dutch Healthcare Authority that now is the right time to evaluate whether the evidence-based guideline recommendations changed healthcare utilization in the Netherlands between 2007 and 2020. We focused on number of procedures, timing of surgical treatment for LDH inflicted sciatica, and healthcare pathways. Secondary, we were interested in changes in societal and healthcare costs.

For peer review only - http://bmjopen.bmj.com/site/about/guidelines.xhtml

METHODS

A population-based, longitudinal, cross-sectional study was performed. The objective of this analysis was to evaluate the impact of guideline implementation on patients with sciatica attributed to lumbar disc herniation (LDH).

Data sources

Two databases were analyzed; firstly, a comprehensive nationwide, administrative healthcare database provided by the Dutch Healthcare Authority. This database contains detailed information on diagnosis by diagnosis-related groups, healthcare activities (e.g. surgical procedures) and fundamental patient characteristics (e.g. gender, year of birth) covering all patients treated in hospitals throughout the Netherlands(16). Secondly, NIVEL's (Netherlands Institute for Health Services Research) Primary Care Database was analyzed. This database comprises routine healthcare data recorded between 2012 and 2020 and represents a 10% sample of all general practitioner (GP) practices in the country (17). To ensure patient confidentiality, individuals in both databases were linked using a unique anonymized identifier, thereby preventing access to sensitive personal patient data by the researchers.

Population

This study focused on Dutch individuals aged 18 years and above who sought medical care at hospitals for sciatica due to a lumbar disc herniation between 2007 and 2020. The authors hypothesized that these patients would possess a registered diagnosis of LDH, have undergone discectomy as a treatment modality, and belong to a younger age group (<56 years) in comparison to individuals with spinal stenosis—a degenerative condition commonly observed in the elderly. This last hypothesis was based on the age distribution of patients included in the SPORT trial and Sciatica trial(6, 18). However, to mitigate potential data incompleteness, as different healthcare providers may employ varying registration practices, the initial study group incorporated multiple hospital diagnosis codes for lumbar degenerative disc disease (DDD), including the code for spinal stenosis, and corresponding surgical procedure codes (discectomy, laminectomy, and instrumented spinal fusion) (Table S1 and S2). Exclusion criteria included patients with cervical degenerative disc disease, spinal infection, traumatic or oncological fractures, spinal deformities, and those who had undergone previous back surgery within the past year. From this

For peer review only

For peer review only - http://bmjopen.bmj.com/site/about/guidelines.xhtml

initial study group, we selected the three subgroups for lumbar disc herniations based on type of surgery, age, and diagnosis code as stated above (For details, see table S3). In summary, the three subgroups consisted of the following patients:

1) Lumbar disc herniation group: this group only included diagnosis codes '1360' (Hernia Nuclei Pulposis) and '1203' (Radicular syndrome), while keeping the same exclusion criteria as the initial study group. All surgical procedure codes were included in this group. These diagnosis codes are registered by orthopedic surgeons and neurologist respectively after confirmation of the diagnosis. Neurologists refer patients if they might be eligible for surgery to a neurosurgeon. Orthopedic surgeons also refer patients to neurosurgeons, but in some clinics they perform surgeries themselves or together with neurosurgeons.
2) Discectomy group: this group used the same inclusion and exclusion criteria as the initial study group, but only included surgical procedure codes for discectomy.
3) Age <56 years group: this group was based on the initial study group, but only included patients younger than 56 years. All surgical procedure codes were included in this group.

In the NIVEL database, adult patients diagnosed with lumbo-radicular syndrome (coded as L86) were included.

Data collection

Firstly, annual number of hospital visits and procedures were collected. Furthermore, we collected patients' age and gender, hospital type (general hospital, (top clinical) teaching hospital, university hospital, or private clinic), attending medical specialist (neurology, neurosurgery, or orthopedic surgery), date of first hospital visit, date of surgery and information on hospital waiting time for hernia-related procedures (days between order request and day of procedure).

For peer review only

Surgical patients were assigned to the hospital where the surgery was performed. Non-surgical patients were assigned to the last hospital they visited. From the NIVEL database, date of first visit to the GP was collected.

Analyses

Our primary outcome included all patients with a registered diagnosis of LDH. Absolute numbers of diagnoses and procedures were calculated between 2007 and 2020. To provide a more accurate representation, surgical rates were adjusted based on the population of individuals aged 18 years and older residing in the Netherlands. Adjusted surgical rates were calculated per 10,000 inhabitants, thereby normalizing the data. Additionally, we calculated in-hospital surgical rates to assess the proportion of referrals with an indication for surgical intervention. Hospital visitors for the primary outcome only included visitors with a registered LDH diagnosis code. Secondary outcomes to validate our findings included the number of discectomies and the number of procedures (discectomies, laminectomies, and fusion) in the age group < 56 years, for which hospitals visitors included all LDD patients. Directly adjusted surgical rates per 10,000 inhabitants or 10,000 hospital visitors were calculated using the following formula:

$\frac{Number\ of\ surgical\ procedures}{Number\ of\ inhabitants\ \geq 18\ years\ /\ hospital\ visitors} \times 10{,}000$Furthermore, the timing of surgical interventions was analyzed by calculating the median number of days between the initial visit to the general practitioner (GP) and the date of surgery. Lastly, we assessed the median number of days between first visit hospital consultation and date of surgical procedure.

Cost analysis

Differences in national healthcare and societal costs were calculated by comparing the annual number of LDH patients per 10,000 over 2007-2009 to that number over pre-covid years 2017-2019. We updated our previous economic evaluation(9) to the population size and price level in 2022, using current surgical prices and updating other costs by the Dutch consumer price index(16, 19). Thus, the difference between a surgical and a conservative policy was estimated at €2596 in healthcare costs and €107 in societal costs per patient. With a 49% difference in surgery probability between both policies(9), these

For peer review only - http://bmjopen.bmj.com/site/about/guidelines.xhtml

estimates translate to the slightly more than double €5298 in healthcare savings and €218 societal savings per prevented surgery. Societal savings are negligible, because the conservative policy has higher productivity costs.

Analytic approach

All analyses were conducted at the individual patient level. To assess differences in annual healthcare utilization, a comparison was made between the years preceding the publication of the guidelines (2007-2009) and the most recent years (2017-2019). Due to the limited usefulness of the data from the COVID-impacted year 2020, it was included in the database but not used for direct comparisons. However, the inclusion of 2020 was deemed important to demonstrate the potential impact of the pandemic on healthcare utilization. A larger decrease in procedures in 2020 might reflect undertreatment, which can lead to chronic disability of patients waiting too long with persistent sciatica leading to chronic disease. We chose for a descriptive analysis, considering the anticipated statistical significance of even small differences given the large size of the database. All analyses were performed using R version 4.1.3(20).

Patient and public involvement in research

Patients were not involved in this research.

RESULTS

Study population

During the period from 2007 to 2020, a total of 1.9 million patients sought hospital care for lumbar degenerative disc disease (DDD). Median age of these patients was 56 years and 54% was female (Table 1). Among these patients, 12% underwent discectomy, laminectomy or fusion surgery (n=217.166). The most common surgical procedure was a discectomy (6%) or laminectomy (6%), while instrumented spinal fusion was less frequently utilized. In 53% of all cases, surgical patients were older than 56 years old and 51% was male. Teaching hospitals accounted for 52% of all procedures. Between 2012 and 2020,

For peer review only

the NIVEL database, specifically the L86 code representing lumbo-radicular syndrome, matched with the hospital database, resulting in 52,057 patients being included in the analysis. Of these patients, 53% was female (compared to 54% in the hospital database), and the mean age was 56 years (compared to 52.4 in 2007 and 57.6 in 2020 in the hospital database). Moreover, among these patients, 16% (8,267 individuals) underwent surgery, showing a slightly higher surgical rate compared to the hospital database, which reported a 12% surgical rate.

Changes in healthcare utilization between 2007-2009 and 2017-2019

Absolute number of procedures for sciatica patients decreased 18% in the LDH group, 22% in the discectomy group, and 18% in the age <56 years group, although an initial increase in number of procedures was observed in all three groups between 2007 and 2010 (Figure 1, Table S4). Annual number of surgical procedures for LDH patients in private clinics increased from 719 in the years 2007-2009 to 1699 in the years 2017-2019 (Figure 2, Table S4). Number of LDH patients in teaching hospitals increased as well (+40%), whereas numbers of surgical procedures for LDH patients decreased in teaching hospitals (-22%). Number of visitors and procedures for LDH patients decreased in general hospitals (-18%; -45%) and university hospitals (-9%; -35%) in the period 2017-2019 compared to 2007-2009.

Changes in healthcare pathways between 2007 and 2019

The number of hospital visitors diagnosed with LDH increased from 55,581 in 2007 to 68,997 in 2019, as shown in Figure 3 and detailed in Table S5. Concurrently, the absolute number of LDH patients referred by neurologists to surgical departments rose from 7,155 in 2007 to 9,398 in 2019, representing a 31% increase over the study period. However, the surgical rate per 10,000 LDH hospital visitors declined from 1,441 per 10,000 in 2007 to 1,012 per 10,000 in 2019 (Figure 3, Table S4). Notably, this decrease was more pronounced among neurosurgeons, dropping from 80% in 2007 to 51% in 2019, compared to orthopedic surgeons, whose surgical rate decreased from 11% in 2007 to 8% in 2019. In terms of referrals between hospitals for surgical and non-surgical LDH patients, there was no substantial change observed between 2007 and 2020 across different hospital types, as depicted in Figure 4.

For peer review only

For peer review only - http://bmjopen.bmj.com/site/about/guidelines.xhtml

Timing of surgery between 2012 and 2020

The median duration in days between the initial diagnosis at the general practitioner (GP) and the date of surgery exhibited an increase from 154 days in 2012 to 196 days in 2019, as shown in Table S6. However, the time interval between the first GP visit and the first hospital visit, also known as the time to referral, remained stable (155 days). Moreover, days between first hospital visit and date of surgery increased from 71 days to 93 days (+22 days). Concurrently, the waiting time for hernia-related procedures in hospitals also increased, with an interval of 31 days in 2012 expanding to 43 days in 2019, reflecting a 12-day prolongation. Among patients below the age of 56 years, there was a decrease in the percentage of individuals who underwent surgery within 12 weeks from the time of diagnosis, declining from 31% in 2012 to 20% in 2019. Conversely, there was an increase in the proportion of patients who received surgery after 20 weeks from diagnosis, rising from 49% in 2012 to 62% in 2019. These findings highlight a shift towards longer waiting periods for surgical intervention in this patient group.

Cost analysis

Changes in healthcare utilization for sciatica due to LDH led to an estimated decrease of 1.5 per 10,000 inhabitants aged 18 years and older living in the Netherlands. For 2022, the associated savings were estimated at a considerable 10 million euro on healthcare costs and only 0.5 million euro on societal costs.

DISCUSSION

Immediately following the publication of the Sciatica trial(6), an initial increase in surgical procedures for LDH patients was observed in the Netherlands. However, after 2010, a gradual decrease in the total numbers of procedures occurred, resulting in substantial annual reductions in healthcare costs and in societal costs. The largest decrease was observed between 2014 and 2016. Noteworthy changes in healthcare pathways revealed an increase in the number

For peer review only - http://bmjopen.bmj.com/site/about/guidelines.xhtml

of referrals per surgeon, while the in-hospital surgical rate decreased. These findings suggest that both surgeons and patients are increasingly opting for an initial conservative treatment rather than early surgery.

Regarding the timing of disc surgery for sciatica, there were notable changes between 2012 and 2019. The median time interval between the initial GP visit and the date of surgery increased, resulting in 82% of all patients undergoing surgery more than 12 weeks after disease onset in 2019. It is important to note that the recommended time window for surgery, as advised in the 2009 Lumbo-Radicular Syndrome (LRS) guideline, indicating surgery at least 12 weeks after disease onset for the expectant wait-and-see period, was already met in 2012. Additionally, hospital waiting times for surgery also increased between 2012 and 2019, which does not necessarily align with surgeon's treatment choices. Moreover, there was an increase in the percentage of patients who received surgery after more than 20 weeks. Although not outlined in the current guideline, the Dutch choosing wisely campaign states that surgery should be performed between 3 and 9 months after disease onset. Although some patients might experience a longer waiting time, our results indicate that most people receive surgery within six months after disease onset. However, recent research showed that 20 weeks after disease onset, outcomes are significantly worse in the even more prolonged conservative care group beyond the guideline time window compared to surgical treatment(3). Therefore, a careful caution in patient guidance is warranted to prevent chronic disabling sciatica, due to irreversible damage to lumbosacral nerve roots by chronic compression(21). As this Canadian study was published during Covid times and not yet implemented in Dutch evidence-based guidelines(3), it is not possible to do a valid investigation in the proportion of patients with a too late indication for surgery. These study findings should be incorporate in a new set of guideline recommendations in the Netherlands.

Our findings are important, because the Netherlands used to perform the second-most operations of the lumbar spine per 100,000 inhabitants compared to other high-income countries(4). The number of procedures for LDH, however, increased in the first years after publication of the Sciatica trial(6). This increasing rate probably resulted from the published fact that study outcomes showed that surgery leads to faster pain relief compared to conservative treatment and was cost-effective compared to conservative care with possibly delayed surgery(9). The decrease after 2010 might have been initiated by the publication of guideline recommendations in 2009, but also by the observation of high practice variation in surgical treatment for LDH in the Netherlands in

For peer review only

For peer review only - http://bmjopen.bmj.com/site/about/guidelines.xhtml

2010(22) leading to a task force of the medical specialist federation (FMS in collaboration with ZonMW) starting the Netherlands version of the international Choosing Wisely campaign. This campaign was launched in 2013 and potentially had a huge impact, since the largest decrease in number of procedures was observed between 2014 and 2016. The active dissemination of the guideline, collaboration of FMS/ZonmW with insurance companies and Patient Federation in the development of wise choices led to awareness of the favorable natural course in most sciatica patients. Not unimportantly the growing attention for evidence-based surgery(23) within the medical specialist scientific societies, probably speeded up the implementation of the guideline in clinical practice(24).

A notable shift in decision-making was observed among neurosurgeons and orthopedic surgeons, who increasingly adhered to the guideline recommendations. In contrast, general practitioners (GPs) and neurologists showed a tendency to refer even more patients with lumbar disc herniation (LDH) than before. Furthermore, the time interval between the first GP visit and the first hospital visit remained unchanged between 2012 and 2019. This observation may indicate that patients have a preference for consulting a surgeon to discuss treatment options for LDH or that healthcare delivery for LDH patients is increasingly organized through multidisciplinary LDH teams, as described by Michael Porter(25). Many hospitals in the Netherlands have implemented Integrated Practice Units, so called ‘Herniated Disc-Diagnosis-Treatment Streets’, where patients receive a joint appointment with both a neurologist and a surgeon, facilitating collaborative decision-making and comprehensive care.

In contrast to other hospital types, a tremendous increase in numbers of procedures for LDH patients was observed in private clinics. This might be attributed to a nationwide strategy aimed at redistributing and decentralizing high-volume, low-complexity diagnoses such as sciatica from university and large teaching hospitals to private clinics(26). This approach helps to concentrate resources and expertise in the larger hospitals, which primarily handle more complex cases. This results in a relief of pressure on the large hospitals, which was especially important during the COVID-19 pandemic: care could be maintained for these patients that required urgent or emergency neurosurgical care(27).

A strength of this study is the nationwide coverage of the database. The fact that academic physicians performed this analysis together with policy makers from the Dutch Healthcare Authority is another strength of this study and supports the independence of the study results.

For peer review only

It must be mentioned, however, that two of the current supervising investigators were the Sciatica Trial's health economist (WH) and principal investigator (WP), while the latter was also involved in the Guideline construction and leader of the Netherlands Choosing Wisely campaign on behalf of FMS and ZonMW. Although both researchers were initially kept out of the data analysis of the current study, some influence might be inevitable. Another limitation of this study is the fact that it is not possible to prove causation between guideline publication and changes in healthcare utilization. Measuring the true impact of the Sciatica trial was challenging due to the unavailability of high-quality data prior to 2007. The most recent available data on the total number of lumbar disc surgeries for sciatica in the Netherlands dates back to 1996, with 11,323 patients per year(5). Similarly, in Finland the largest reduction in discectomies occured before 2007(28), probably caused by the starting awareness of ongoing studies in USA and Europe and the earlier landmark publication by Cherkin and Deyo warning against overtreatment in spine surgery(4). The last limitation of this study is the use of administrative healthcare data. The use of these data enables to perform this investigation, but data might reflect differences in registration or coding. For example, the GP code L86 might also include lumbar stenosis. By using the three patient identifiers (i.e. age, discectomy, and registered code for LDH), we tried to minimize this limitation and validate our findings.

CONCLUSION

Although causation cannot be proven, this study highlights the substantial impact of evidence-based guidelines on clinical practice. We observed a decrease in the number of discectomy procedures, with a longer timeframe between the initial visit to the general practitioner and surgery and subsequently a reduction in healthcare costs. The decrease in number of procedures suggest that the 2008 guideline recommendations for surgical treatment for sciatica resulting from lumbar disc herniation (LDH) were effectively implemented in clinical practice in the Netherlands, especially after the dissemination of evidence-based guidelines and the Choosing Wisely Campaign. The observed longer-time frame observed in this study, agrees with the current Dutch guideline, but recent research showed that waiting too long might lead to chronic disability. To ensure the continued improvement of quality of care, it is essential to incorporate the findings of studies such as Bailey et al. into evidence-based guidelines and clinical practice, thereby preventing undertreatment of LDH patients in the future.

CONTRIBUTORSHIP STATEMENT

For peer review only - http://bmjopen.bmj.com/site/about/guidelines.xhtml

JJCMvM designed the data-analysis and data-collection plan, wrote the statistical analysis plan, and drafted and revised the paper. She is guarantor. MWN analyzed the data and revised the draft paper. IJYH revised the draft paper. WBvdH devised and supervised the cost analysis plan and revised the draft paper. PPGvB revised the draft paper. IS monitored the data collection and analysis and revised the draft paper. WM monitored the data-analysis and data-collection plan and revised the draft paper. WCP initiated the collaborative project, monitored the data-analysis and data-collection plan and revised the draft paper.

COMPETING INTERESTS

Prof. dr. Peul: Boardmember ZonMW, former Chair Netherlands Choosing Wisely FMS committee.

Prof. dr. van Benthem: Former president of the Dutch Association of Medical Specialists (FMS).

Dr. W. Moojen is a Board member of the Dutch Neurosurgical Society.

Prof. dr. Peul and dr. W. van den Hout were involved in the design and execution of the sciatica trial. Furthermore, prof dr. Peul was also involved in the Guideline construction and leader of the Netherlands Choosing Wisely campaign on behalf of FMS and ZonMW.

FUNDING

Supported by a grant from: the Netherlands Organization for Health Research and Development (Citrienfonds), grant number: 839205002. The funding organization had no role in the present study.

DATA SHARING STATEMENT

Datasets utilized for this study were constructed on data servers hosted by the Dutch Healthcare Authority. Notably, the dataset used is not available for external parties. However, the data originate from non-public microdata obtained from Statistics Netherlands (CBS). Under certain conditions, these microdata are accessible for statistical and scientific research. For further information: microdata@cbs.nl.

REFERENCES

For peer review only

1. Ravindra VM, Senglaub SS, Rattani A, Dewan MC, Härtl R, Bisson E, et al. Degenerative Lumbar Spine Disease: Estimating Global Incidence and Worldwide Volume. Global Spine J. 2018;8(8):784-94.
2. Hofstee DJ, Gijtenbeek JM, Hoogland PH, van Houwelingen HC, Kloet A, Lötters F, et al. Westeinde sciatica trial: randomized controlled study of bed rest and physiotherapy for acute sciatica. J Neurosurg. 2002;96(1 Suppl):45-9.
3. Bailey CS, Rasoulinejad P, Taylor D, Sequeira K, Miller T, Watson J, et al. Surgery versus Conservative Care for Persistent Sciatica Lasting 4 to 12 Months. New England Journal of Medicine. 2020;382(12):1093-102.
4. Cherkin DC, Deyo RA, Loeser JD, Bush T, Waddell G. An international comparison of back surgery rates. Spine (Phila Pa 1976). 1994;19(11):1201-6.
5. Gezondheidsraad. Diagnostiek en behandeling van het radiculaire syndroom. In: Gezondheidsraad, editor. Den Haag1999.
6. Peul WC, van Houwelingen HC, van den Hout WB, Brand R, Eekhof JA, Tans JT, et al. Surgery versus prolonged conservative treatment for sciatica. N Engl J Med. 2007;356(22):2245-56.
7. Weinstein JN, Tosteson TD, Lurie JD, Tosteson AN, Hanscom B, Skinner JS, et al. Surgical vs nonoperative treatment for lumbar disk herniation: the Spine Patient Outcomes Research Trial (SPORT): a randomized trial. Jama. 2006;296(20):2441-50.
8. Weinstein JN, Tosteson TD, Lurie JD, Tosteson ANA, Hanscom B, Skinner JS, et al. Surgical vs Nonoperative Treatment for Lumbar Disk HerniationThe Spine Patient Outcomes Research Trial (SPORT): A Randomized Trial. JAMA. 2006;296(20):2441-50.
9. van den Hout WB, Peul WC, Koes BW, Brand R, Kievit J, Thomeer RT. Prolonged conservative care versus early surgery in patients with sciatica from lumbar disc herniation: cost utility analysis alongside a randomised controlled trial. Bmj. 2008;336(7657):1351-4.
10. CBO KvdG. Lumbosacraal Radiculair Syndroom 2008.
11. Krendl R. Lumbale Radikulopathie. Springer; 2014.
12. Kreiner DS, Hwang S, Easa J, Resnick DK, Baisden J, Bess S, et al., editors. Diagnosis and treatment of lumbar disc herniation with radiculopathy. North American Spine Society; 2012.
13. Verstandige keuzes bij een lage rughernia.
14. Levinson W, Kallewaard M, Bhatia RS, Wolfson D, Shortt S, Kerr EA. 'Choosing Wisely': a growing international campaign. BMJ Qual Saf. 2015;24(2):167-74.
15. Schaafstra AS, W.E.M.; Bons, S.C.S.; Borg, M.A.J.P.; Koes, B.W.; Ostelo, R.W.J.G.; Spijker-Huiges, A.; Burgers, J.S.; Bouma, M.; Verburg-Oorthuizen, A.F.E. Lumbosacraal radiculair syndroom. In: NHG-werkgroep, editor. 2015.
16. Statistics Netherlands Netherlands [Available from: https://www.cbs.nl/en-gb.
17. Netherlands Institute for Health Services Research [Available from: https://www.nivel.nl/en.
18. Cummins J, Lurie JD, Tosteson TD, Hanscom B, Abdu WA, Birkmeyer NJO, et al. Descriptive Epidemiology and Prior Healthcare Utilization of Patients in the Spine Patient Outcomes Research Trial's (SPORT) Three Observational Cohorts: Disc Herniation, Spinal Stenosis, and Degenerative Spondylolisthesis. Spine. 2006;31(7):806-14.
19. Hakkaart-van Roijen L, van der Linden N, Bouwmans C, Kanters T, Tan SS. Kostenhandleiding: Methodologie van kostenonderzoek enreferentieprijzen voor economische evaluatiesin de gezondheidszorg. Institute for Medical Technology Assessment, Erasmus Universiteit Rotterdam; 2012.
20. studio R. 4.1.3. ed2022.
21. Nygaard OP, Romner B, Trumpy JH. Duration of symptoms as a predictor of outcome after lumbar disc surgery. Acta Neurochir (Wien). 1994;128(1-4):53-6.

For peer review only - http://bmjopen.bmj.com/site/about/guidelines.xhtml

22. van Beek EL, K.; van Schooten, G.; Vlieger, E.J. Reduceren van praktijkvariatie: budgettaire effecten van scherpere indicatiestelling. In: Ministerie van Volksgezondheid WeS, editor. 2010.
23. McCulloch P, Altman DG, Campbell WB, Flum DR, Glasziou P, Marshall JC, et al. No surgical innovation without evaluation: the IDEAL recommendations. Lancet. 2009;374(9695):1105-12.
24. Lomas J. Words without action? The production, dissemination, and impact of consensus recommendations. Annu Rev Public Health. 1991;12:41-65.
25. Porter ME, Teisberg EO. Redefining competition in health care. Harv Bus Rev. 2004;82(6):64-76, 136.
26. Aandeel zelfstandige behandelcentra blijft toenemen 2018 [Available from: https://www.vektis.nl/actueel/aandeel-zelfstandige-behandelcentra-blijft-toenemen.
27. Ashkan K, Jung J, Velicu AM, Raslan A, Faruque M, Kulkarni P, et al. Neurosurgery and coronavirus: impact and challenges—lessons learnt from the first wave of a global pandemic. Acta Neurochirurgica. 2021;163(2):317-29.
28. Ponkilainen VT, Mäntymäki H, Huttunen TT, Mattila VM. Decreasing Incidence of Lumbar Discectomy Surgery in Finland in 1997-2018. Spine (Phila Pa 1976). 2021;46(6):383-90.

For peer review only

Table 1. baseline characteristics of the total lumbar degenerative disc disease population between 2007 and 2020

Characteristic	Total number of lumbar DDD patients (2007-2020, n=1863627 (100%[a]))	Surgically treated patients (2007-2020, n=217166 (100%))
**Age group (n, %)**		
18-45 years	523431 (28)	58648 (27)
46-55 years	391292 (21)	42370 (20)
56 years and older	948904 (51)	116098 (53)
**Gender, (n, %)**		
Male	856732 (46)	110922 (51)
Female	1006895 (54)	106194 (49)
**Diagnosis, (n, %)**		
Lumbar disc herniation	870878 (47)	108036 (50)
Lumbar stenosis	317360 (17)	65977 (30)
Lumbar disc herniation and lumbar stenosis	46050 (2)	31045 (14)
Low back pain	587695 (32)	4899 (2)
Unknown	41644 (2)	7159 (3)
**Hospital type, (n, %)**		
General hospital	736250 (40)	63892 (29)
Teaching hospital	884234 (47)	113444 (52)
University hospital	94071 (5)	13627 (6)
Private clinic	149072 (8)	26153 (12)
**Department, (n, %)**		
Neurology department	1464011 (79)	142786 (66)
Neurosurgery department	303626 (16)	189418 (87)
Orthopedic surgery department	356674 (19)	39172 (18)

For peer review only

For peer review only - http://bmjopen.bmj.com/site/about/guidelines.xhtml

**Type of treatment[b], (n, %)**		
Discectomy	107549 (6)	107549 (50)
Laminectomy	109477 (6)	109477 (9)
Fusion	20449 (1)	20449 (50)
No surgical treatment	1626152 (88)	-
**Referred from another hospital (n=1777532[c]), (n, %)**		
Yes	85164 (5)	44793 (26)
No	1692368 (95)	126835 (74)
[a] due to rounding, the number of percentages might not add up to 100% [b] due to the fact that patients might receive two types of surgery, the percentages might not add up to 100% [c] Patients who visited more than one hospital on the same day were not included in this group		

For peer review only

Figures

Figure 1. trend the absolute number of procedures and the directly standardized number of procedures; the left Y-axis and bars show the absolute number of procedures. The right Y-axis and lines show the number of procedures per 10,000 inhabitants (age > 18 years).

Figure 2. trends in absolute number of procedures per hospital type. The left Y-axis and bars show the absolute number of surgical procedures, the right Y-axis and lines show the number of hospitals performing back surgeries.

Figure 3. GP referrals and in-hospital surgical rates. The left Y-axis and bars show the absolute number of surgical procedures, the right Y-axis and lines show the number of procedures per 10,000 hospital visitors (in-hospital surgical rate; age 18 years and older)

Figure 4. number of referrals from another hospital for surgical and non-surgical patients per hospital type. The Y-axis shows the percentage of patients that were referred from another hospital per hospital type.

For peer review only - http://bmjopen.bmj.com/site/about/guidelines.xhtml

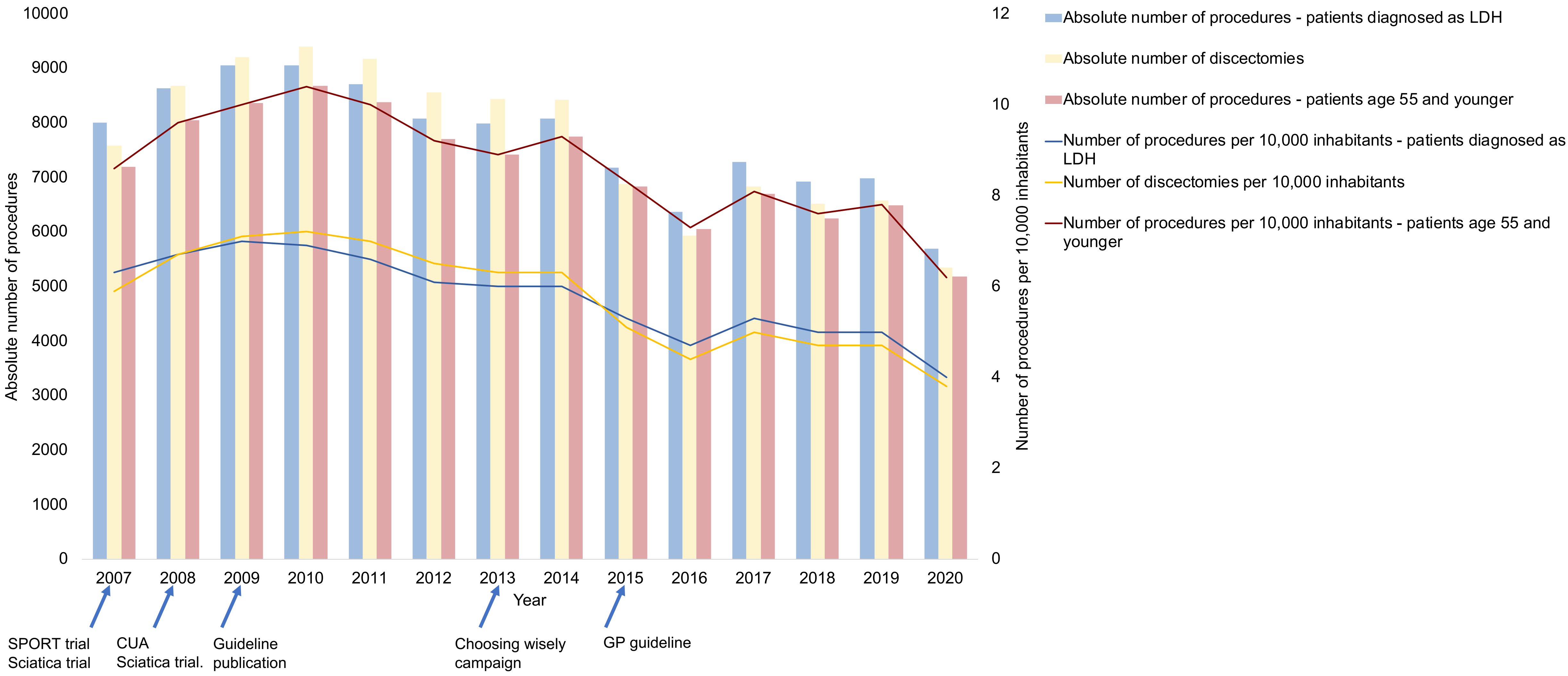
10000
9000
8000
7000
6000
5000
4000
3000
2000
1000
0
Absolute number of procedures
12
10
8
6
4
2
0
Number of procedures per 10,000 inhabitants
2007
2008
2009
2010
2011
2012
2013
2014
2015
2016
2017
2018
2019
2020
Year
SPORT trial Sciatica trial
CUA Sciatica trial.
Guideline publication
Choosing wisely campaign
GP guideline
Absolute number of procedures - patients diagnosed as LDH
Absolute number of discectomies
Absolute number of procedures - patients age 55 and younger
Number of procedures per 10,000 inhabitants - patients diagnosed as LDH
Number of discectomies per 10,000 inhabitants
Number of procedures per 10,000 inhabitants - patients age 55 and younger

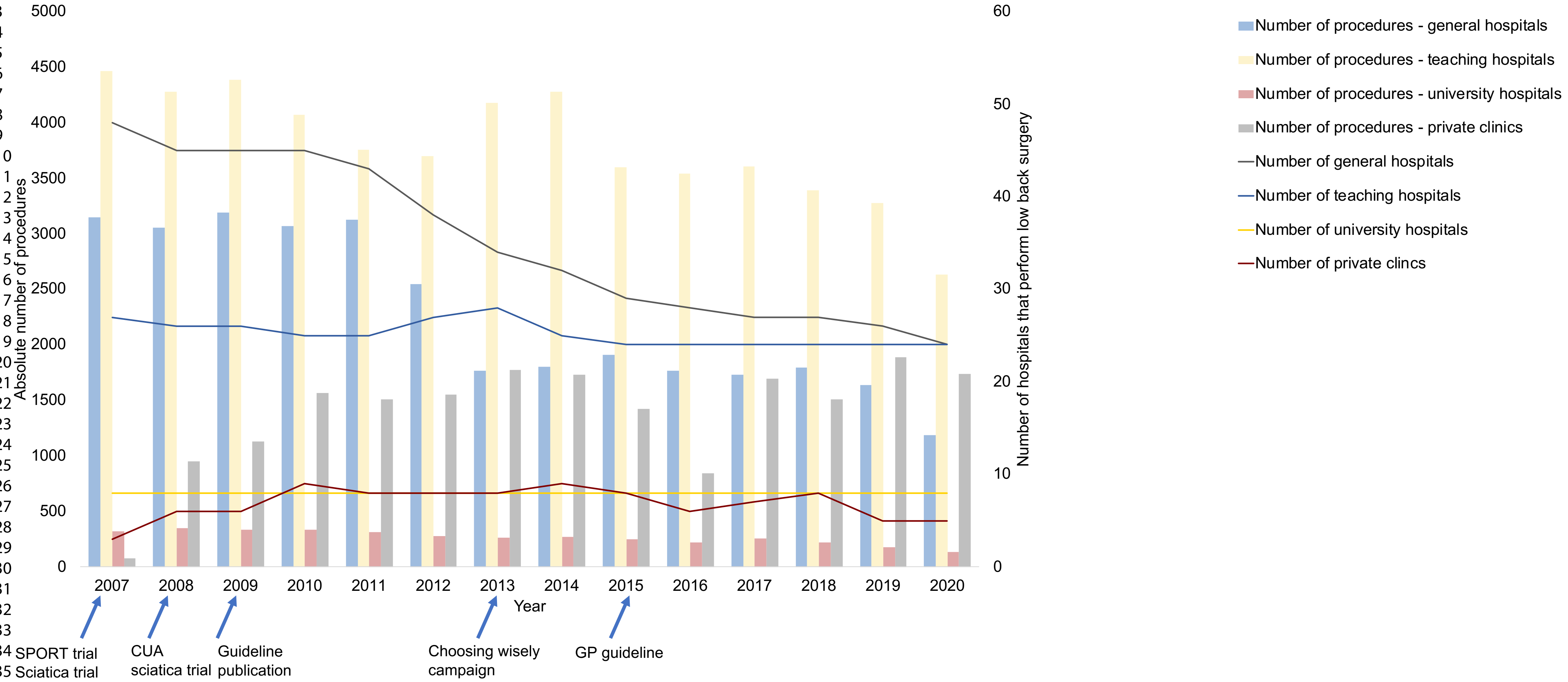
5000
4500
4000
3500
3000
2500
2000
1500
1000
500
0
Absolute number of procedures
60
50
40
30
20
10
0
Number of hospitals that perform low back surgery
2007
2008
2009
2010
2011
2012
2013
2014
2015
2016
2017
2018
2019
2020
Year
Number of procedures - general hospitals
Number of procedures - teaching hospitals
Number of procedures - university hospitals
Number of procedures - private clinics
Number of general hospitals
Number of teaching hospitals
Number of university hospitals
Number of private clincs
SPORT trial
Sciatica trial
CUA
sciatica trial
Guideline
publication
Choosing wisely
campaign
GP guideline

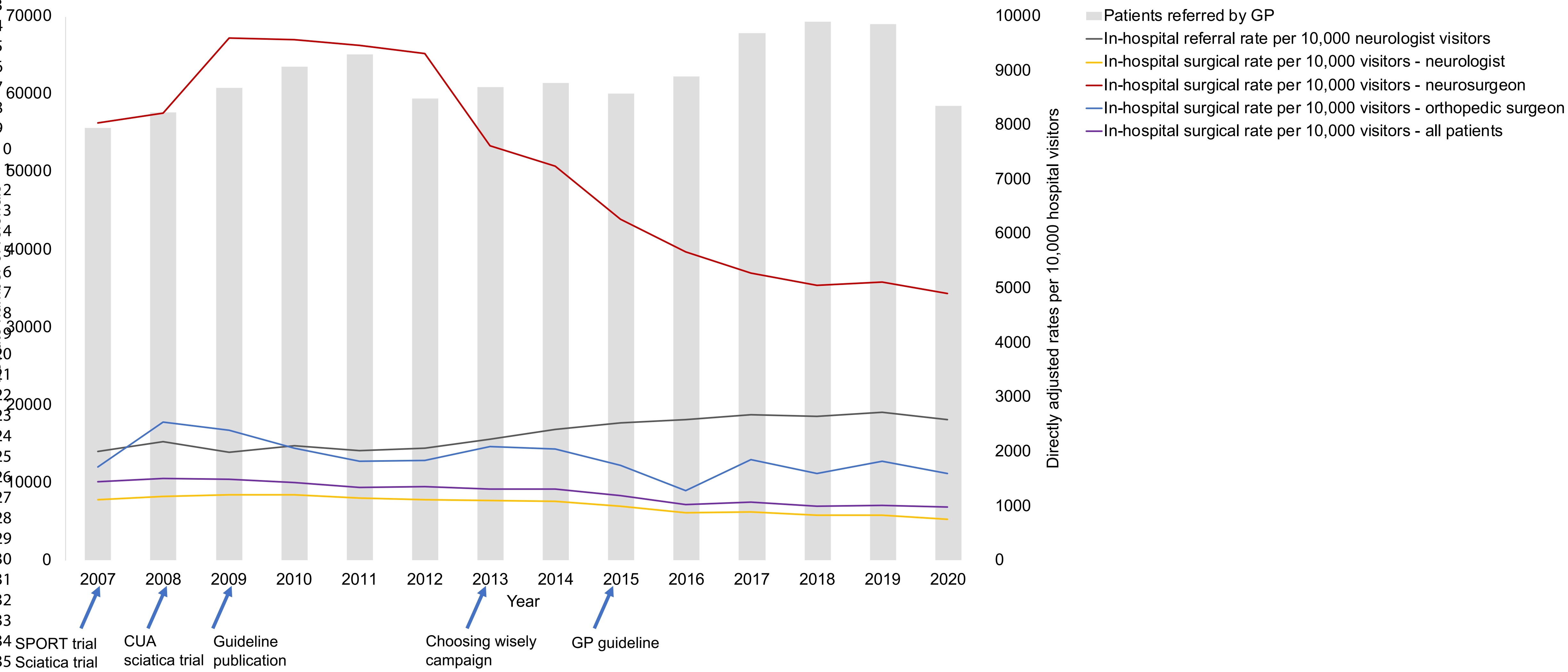
70000
60000
50000
40000
30000
20000
10000
0
Absolute number of referrals
10000
9000
8000
7000
6000
5000
4000
3000
2000
1000
0
Directly adjusted rates per 10,000 hospital visitors
2007
2008
2009
2010
2011
2012
2013
2014
2015
2016
2017
2018
2019
2020
Year
Patients referred by GP
In-hospital referral rate per 10,000 neurologist visitors
In-hospital surgical rate per 10,000 visitors - neurologist
In-hospital surgical rate per 10,000 visitors - neurosurgeon
In-hospital surgical rate per 10,000 visitors - orthopedic surgeon
In-hospital surgical rate per 10,000 visitors - all patients
SPORT trial
Sciatica trial
CUA
sciatica trial
Guideline
publication
Choosing wisely
campaign
GP guideline

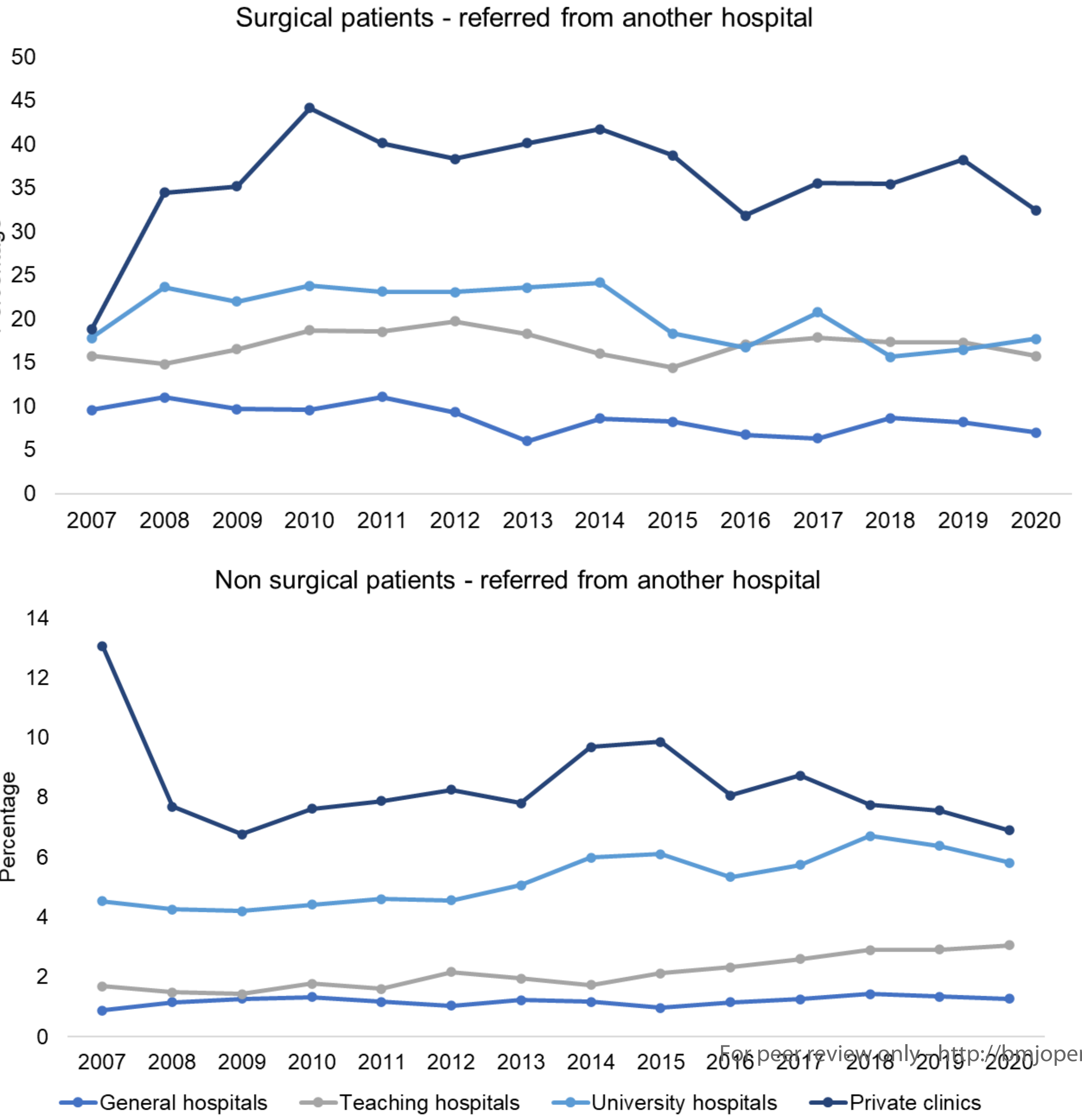

For peer review only - http://bmjopen.bmj.com/site/about/guidelines.xhtml

APPENDIX

Table S1. Used diagnosis treatment codes

Diagnosis treatment codes	Description
Included	
0330-12-00-1203	Pseudo radicular syndrome
0330-12-00-1204	Radicular syndrome
0330-12-00-1211<br>0305-13-00-1350	Spinal stenosis
0305-13-00-1360	Hernia Nuclei Pulposis
0305-13-00-1370	Spondylolisthesis
0308-02-25-2501<br>0308-02-25-2550<br>0308-02-25-2565<br>0308-02-25-2555<br>0308-02-25-2505<br>0308-02-25-2510<br>0308-02-25-2515	Degenerative disc disease
0330-12-00-1231<br>0305-13-00-1330<br>0305-13-00-1340	Low backpain
Excluded	
0330-12-00-1201<br>0330-12-00-1202<br>0330-05-00-0543<br>0305-12-00-1202<br>0305-12-00-1220<br>0305-12-00-1240<br>0308-02-25-2525<br>0308-02-25-2527<br>0308-02-25-2530<br>0308-02-25-2535	Cervical diagnosis codes
0330-02-00-0232<br>0330-02-00-0233<br>0330-02-00-0221<br>0330-02-00-0222<br>0330-02-00-0223<br>0308-02-21-2105<br>0308-02-21-2110	Tumors, malignancies of the spine

For peer review only

0308-02-21-2115	
0308-02-24-2405<br>0308-02-24-2411<br>0305-13-00-1301<br>0305-13-00-1302	Infectious spine disease
0305-13-00-1396<br>0308-02-23-2305<br>0308-02-23-2311	Spine trauma, fractures
0305-13-00-1397<br>0305-13-00-1380<br>0305-13-00-1381<br>0305-13-00-1383<br>0305-13-00-1391	Congenital disc disease, kyphosis, scoliosis

Table S2. Used surgical procedure codes

**Care product code**	**Description**
Included	
38437<br>38444<br>38438<br>38467	Discectomy
30327<br>30329	Laminectomy
38458<br>38459<br>38460<br>38464<br>38468<br>38469	Fusion

Table S3. Inclusion criteria for the LDH subgroups

**Lumbar disc herniation diagnosis group**	
Included diagnosis codes	
0305-13-00-1360	Hernia Nuclei Pulposis
0330-12-00-1203	Radicular syndrome
Excluded diagnosis codes	
0330-12-00-1201<br>0330-12-00-1202<br>0330-05-00-0543	Cervical diagnosis codes

For peer review only

0305-12-00-1202<br>0305-12-00-1220<br>0305-12-00-1240<br>0308-02-25-2525<br>0308-02-25-2527<br>0308-02-25-2530<br>0308-02-25-2535	
0330-02-00-0232<br>0330-02-00-0233<br>0330-02-00-0221<br>0330-02-00-0222<br>0330-02-00-0223<br>0308-02-21-2105<br>0308-02-21-2110<br>0308-02-21-2115	Tumors, malignancies of the spine
0308-02-24-2405<br>0308-02-24-2411<br>0305-13-00-1301<br>0305-13-00-1302	Infectious spine disease
0305-13-00-1396<br>0308-02-23-2305<br>0308-02-23-2311	Spine trauma, fractures
0305-13-00-1397<br>0305-13-00-1380<br>0305-13-00-1381<br>0305-13-00-1383<br>0305-13-00-1391	Congenital disc disease, kyphosis, scoliosis
**Included surgical procedure codes**	
38437<br>38444<br>38438<br>38467	Discectomy
30327<br>30329	Laminectomy
38458<br>38459<br>38460<br>38464<br>38468<br>38469	Fusion

**Discectomy group**

For peer review only

Included diagnosis codes	
0330-12-00-1203	Radicular syndrome
0330-12-00-1204	Pseudo radicular syndrome
0330-12-00-1211<br>0305-13-00-1350	Spinal stenosis
0305-13-00-1360	Hernia Nuclei Pulposis
0305-13-00-1370	Spondylolisthesis
0308-02-25-2501<br>0308-02-25-2550<br>0308-02-25-2565<br>0308-02-25-2555<br>0308-02-25-2505<br>0308-02-25-2510<br>0308-02-25-2515	Degenerative disc disease
0330-12-00-1231<br>0305-13-00-1330<br>0305-13-00-1340	Low backpain
Excluded diagnosis codes	
0330-12-00-1201<br>0330-12-00-1202<br>0330-05-00-0543<br>0305-12-00-1202<br>0305-12-00-1220<br>0305-12-00-1240<br>0308-02-25-2525<br>0308-02-25-2527<br>0308-02-25-2530<br>0308-02-25-2535	Cervical diagnosis codes
0330-02-00-0232<br>0330-02-00-0233<br>0330-02-00-0221<br>0330-02-00-0222<br>0330-02-00-0223<br>0308-02-21-2105<br>0308-02-21-2110<br>0308-02-21-2115	Tumors, malignancies of the spine
0308-02-24-2405<br>0308-02-24-2411<br>0305-13-00-1301<br>0305-13-00-1302	Infectious spine disease
0305-13-00-1396<br>0308-02-23-2305	Spine trauma, fractures

For peer review only

0308-02-23-2311	
0305-13-00-1397<br>0305-13-00-1380<br>0305-13-00-1381<br>0305-13-00-1383<br>0305-13-00-1391	Congenital disc disease, kyphosis, scoliosis
**Included surgical procedure codes**	
38437<br>38444<br>38438<br>38467	Discectomy

**Age (<56 years) group**	
Included diagnosis codes	
0330-12-00-1203	Pseudo radicular syndrome
0330-12-00-1204	Radicular syndrome
0330-12-00-1211<br>0305-13-00-1350	Spinal stenosis
0305-13-00-1360	Hernia Nuclei Pulposis
0305-13-00-1370	Spondylolisthesis
0308-02-25-2501<br>0308-02-25-2550<br>0308-02-25-2565<br>0308-02-25-2555<br>0308-02-25-2505<br>0308-02-25-2510<br>0308-02-25-2515	Degenerative disc disease
0330-12-00-1231<br>0305-13-00-1330<br>0305-13-00-1340	Low backpain
Excluded diagnosis codes	
0330-12-00-1201<br>0330-12-00-1202<br>0330-05-00-0543<br>0305-12-00-1202<br>0305-12-00-1220<br>0305-12-00-1240<br>0308-02-25-2525<br>0308-02-25-2527<br>0308-02-25-2530<br>0308-02-25-2535	Cervical diagnosis codes

For peer review only

0330-02-00-0232<br>0330-02-00-0233<br>0330-02-00-0221<br>0330-02-00-0222<br>0330-02-00-0223<br>0308-02-21-2105<br>0308-02-21-2110<br>0308-02-21-2115	Tumors, malignancies of the spine
0308-02-24-2405<br>0308-02-24-2411<br>0305-13-00-1301<br>0305-13-00-1302	Infectious spine disease
0305-13-00-1396<br>0308-02-23-2305<br>0308-02-23-2311	Spine trauma, fractures
0305-13-00-1397<br>0305-13-00-1380<br>0305-13-00-1381<br>0305-13-00-1383<br>0305-13-00-1391	Congenital disc disease, kyphosis, scoliosis
**Included surgical procedure codes**	
38437<br>38444<br>38438<br>38467	Discectomy
30327<br>30329	Laminectomy
38458<br>38459<br>38460<br>38464<br>38468<br>38469	Fusion

For peer review only

Table S4. Full table of surgical trends between 2007 and 2020 for hospital visitors with a likely diagnosis of lumbar disc herniation

	2007	2008	2009	2010	2011	2012	2013	2014	2015	2016	2017	2018	2019	2020
**Patients with a registered diagnosis of LDH[a]**														
Number of procedures	8009	8638	9048	9046	8708	8077	7987	8085	7183	6372	7290	6917	6983	5693
Per 10,000 inhabitants	6.3	6.7	7.0	6.9	6.6	6.1	6.0	6.0	5.3	4.7	5.3	5.0	5.0	4.0
Per 10,000 hospital visitors	1441	1500	1489	1425	1339	1361	1312	1317	1196	1024	1076	998	1012	974
Male	4278	4675	4822	4827	4645	4230	4177	4194	3802	3156	3737	3460	3567	2845
Female	3731	3963	4226	4219	4063	3847	3810	3891	3381	3216	3553	3457	3416	2848
Neurosurgery	6941	6905	7250	7629	7431	7004	6928	6982	6411	5935	6381	6286	6208	5211
Orthopedic surgery	1282	2023	2078	1735	1602	1364	1383	1422	1063	652	1216	932	1088	773
Teaching hospitals	4463	4279	4389	4074	3757	3698	4177	4281	3598	3542	3607	3394	3277	2634
General hospitals	3150	3059	3193	3071	3127	2550	1769	1801	1913	1770	1732	1794	1640	1187
Private clinics	75	950	1132	1565	1509	1554	1775	1734	1421	840	1698	1511	1888	1741
University hospitals	321	350	334	336	315	275	266	269	251	220	253	218	178	131
**Patients who had a discectomy**														
Number of procedures	7586	8673	9204	9403	9172	8561	8443	8425	6872	5931	6828	6522	6585	5344
Per 10,000 inhabitants	5.9	6.7	7.1	7.2	7.0	6.5	6.3	6.3	5.1	4.4	5.0	4.7	4.7	3.8
Per 10,000 hospital visitors	656	702	719	687	658	689	652	625	524	436	472	447	442	427
Male	4084	4714	4898	5021	4944	4504	4438	4379	3676	2951	3489	3259	3348	2645
Female	3502	3959	4306	4382	4228	4057	4005	4046	3196	2980	3339	3263	3237	2699
Neurosurgery	6727	6973	7380	7909	7832	7507	7463	7445	6271	5605	6153	6079	5992	5015
Orthopedic surgery	1083	2040	2167	1888	1736	1445	1403	1400	929	593	1062	806	1023	701
Teaching hospitals	4261	4263	4493	4255	3939	3967	4482	4508	3634	3530	3634	3392	3276	2616
General hospitals	2963	3079	3210	3151	3353	2711	1832	1851	1683	1407	1447	1616	1510	1055
Private clinics	8	960	1152	1634	1561	1589	1822	1775	1289	750	1495	1305	1604	1557
University hospitals	354	371	349	363	319	294	307	291	266	244	252	209	195	116
**Patients age < 56 years**														
Number of procedures	7192	8052	8363	8683	8383	7708	7414	7742	6827	6052	6691	6243	6484	5184
Per 10,000 inhabitants	8.6	9.6	10.0	10.4	10.0	9.2	8.9	9.3	8.3	7.3	8.1	7.6	7.8	6.2
Per 10,000 hospital visitors	1103	1187	1204	1181	1139	1200	1139	1183	1107	971	1028	969	1016	977
Male	3881	4386	4489	4638	4486	4060	3876	4011	3583	3019	3426	3105	3274	2540
Female	3311	3666	3874	4045	3897	3648	3538	3731	3244	3033	3265	3138	3210	2644
Neurosurgery	6198	6376	6632	7152	7054	6619	6371	6633	6085	5575	5807	5625	5694	4732
Orthopedic surgery	1230	2010	2059	1927	1711	1416	1425	1509	1093	753	1242	922	1158	754
Teaching hospitals	4026	3962	4098	3989	3651	3589	3868	4091	3434	3435	3362	3141	3150	2401
General hospitals	2703	2786	2846	2802	2927	2305	1589	1672	1759	1536	1538	1521	1443	1077
Private clinics	58	864	994	1467	1415	1404	1599	1582	1290	758	1473	1304	1625	1509
University hospitals	405	440	425	425	390	410	358	397	344	323	318	277	266	197

For peer review only

Table S5. Trends between 2007 and 2020 for hospital visitors with a likely diagnosis of lumbar disc herniation

	2007	2008	2009	2010	2011	2012	2013	2014	2015	2016	2017	2018	2019	2020
**Patients with a registered diagnosis of LDH[a]**														
Hospital visitors	55581	57597	60777	63493	65023	59365	60883	61396	60040	62217	67771	69296	68997	58442
Per 10,000 inhabitants	43	45	47	49	49	45	46	46	45	46	50	50	50	42
Male	28528	29674	31064	32271	32624	29446	29838	30190	29260	30104	32362	32836	32934	27733
Female	27053	27923	29713	31222	32399	29919	31045	31206	30780	32113	35409	36460	36063	30709
Neurology	48447	50803	53529	56499	57929	52870	54380	54382	53327	56397	60410	62255	62047	52314
Neurosurgery	8643	8409	7560	7970	7859	7522	9099	9644	10240	10487	12084	12444	12145	10648
Orthopedic surgery	7490	7944	8678	8437	8764	7435	6605	6954	6071	5097	6595	5840	5976	4843
Referred by neurologist to surgical department	7155	7526	7005	7506	7346	6896	7541	7790	7991	8180	9521	9457	9398	7838
Teaching hospitals	23509	23736	25298	25115	25964	24972	30872	30979	30855	31844	33391	34414	33453	28581
General hospitals	27714	26461	27189	28910	29079	24980	21083	22322	22026	22598	22525	22241	22224	18534
Private clinics	1774	4806	5797	6912	7461	7005	6518	5547	4963	5334	9394	10315	11097	9301
University hospitals	2584	2594	2493	2556	2519	2408	2410	2548	2196	2441	2461	2326	2223	2026
Mean per hospital														
Teaching hospitals	871	913	973	966	999	892	1065	1192	1234	1274	1336	1377	1338	1143
General hospitals	462	448	486	535	539	480	449	507	551	595	644	635	635	545
Private clinics	197	267	290	288	298	280	251	264	216	254	447	491	555	443
University hospitals	323	324	312	320	315	301	301	319	275	305	308	291	278	253
**Patients age < 56 years**														
Hospital visitors	65185	67861	69449	73503	73569	64257	65093	65463	61695	62308	65056	64399	63808	53077
Per 10,000 inhabitants	78	81	83	88	88	77	78	79	75	75	79	78	77	64
Male	32213	33336	34010	35468	35050	30228	30358	30501	28412	28623	29788	29004	28938	23817
Female	32972	34525	35439	38035	38519	34029	34735	34962	33283	33685	35268	35395	34870	29260
Neurology	48448	51585	54337	57659	57969	50346	50583	50188	47716	48789	50783	50658	11571	40686
Neurosurgery	8902	8747	7131	7665	7615	7342	9499	10158	10629	10300	11270	11415	49746	10581
Orthopedic surgery	15865	16559	16607	17370	17256	14589	13514	14121	11813	11584	12328	11343	11537	9144
Referred by neurologist to surgical department	5164	5562	5205	5574	5507	5021	5410	5488	5414	5281	6071	5928	5763	4656
Teaching hospitals	27871	28569	29173	30248	30182	27595	33030	32593	31141	31510	32770	32911	31988	25935
General	31533	30973	31641	33444	33092	26749	21881	22868	21473	21901	21370	20161	19668	15801
University hospitals	3969	4037	3655	3541	3437	3224	3153	3200	2873	3218	2897	2548	2480	2101
Private clinics	1812	4282	4980	6270	6858	6689	7029	6802	6208	5679	8019	8779	9672	9240
Mean per hospital														
Teaching hospital	214	284	311	338	337	317	332	372	348	348	413	425	467	422
General	513	525	565	627	620	527	482	523	533	574	628	589	586	491
University hospital	1072	1106	1133	1051	1020	884	1070	1226	1300	1314	1262	1225	1347	1006
Private clinics	496	505	457	443	430	403	394	400	359	402	362	319	310	263

[a] Lumbar Disc Herniation

For peer review only

Table S6. Full table of time between diagnosis, first hospital-visit and surgery between 2012 and 2020 (Nivel and NZA database)

	2012 (n=697)	2013 (n=958)	2014 (n=1174)	2015 (n=986)	2016 (n=804)	2017 (n=947)	2018 (n=902)	2019 (n=1031)	2020 (n=744)
Days between first GP[b] visit and first hospital visit (average)	158	155	163	179	180	174	184	169	193
**First GP visit to surgery**									
Days between first GP visit and surgery, median (25th-75th)	154 (80 – 361)	154 (77 – 328)	166 (87 – 351)	183 (91 – 361)	174 (92 – 371)	187 (103 – 354)	202 (104 – 386)	196 (104 – 383)	239 (137 – 451)
Days between first GP visit and surgery, 5th-95th	15 - 695	22 - 756	29 - 795	34 - 803	26 - 822	35 - 824	35 - 857	36 - 803	43 - 840
Days between first GP visit and surgery for patients <56 years, median (25th-75th)	137 (74-332)	147 (71-310)	142 (79-280)	167 (82-341)	161 (86-337)	160 (85-301)	172 (91-346)	183 (91-347)	230 (111-415)
**Time from first hospital visit to surgery**									
Days between first hospital visit and surgery, median (25th-75th)	51 (25-91)	51 (25-91)	56 (26-99)	62 (32-106)	59 (29-96)	70 (35-118)	67 (35-119)	70 (35-125)	80 (40-141)
Days between first hospital visit and surgery, 5th-95th	2 - 223	6 - 207	7 - 223	7 - 201	8 - 212	8 - 223	7 - 223	7 - 239	8 – 264
Days between first hospital visit and surgery for patients <56 years, median (25th-75th)	40 (21-78)	45 (21-81)	45 (21-81)	48 (23-86)	46 (22-78)	63 (31-105)	56 (23-108)	59 (28-110)	63 (31-118)
**Following the guideline?**									
Patients that had surgery within 2 days from diagnosis (GP) (%)	0.7	0.8	0.5	0.5	0.5	0.4	0.3	0.8	0.4
Patients that had surgery between 2 days and 12 weeks from diagnosis (GP) (%)	26.2	26.3	23.2	22.1	21.8	18.3	17.3	17.6	12.4
Patients that had surgery after 12 weeks and before 20 weeks from diagnosis (GP) (%)	20.2	18.6	19.7	16.3	19.4	17.3	17.3	15.7	13.0
Patients that had surgery after 20 weeks from diagnosis (GP) (%)	52.8	54.3	56.4	61.1	58.3	63.9	65.0	65.9	74.2
Patients <56 years that had surgery within 2 days from diagnosis (GP) (%)	1.4	0.8	0.3	0.7	0.3	0.5	0.3	1.1	0.0
Patients <56 years that had surgery between 2 days and 12 weeks from diagnosis (GP) (%)	30.7	28.7	26.8	25.5	24.6	24.3	20.3	20.0	17.2
Patients <age 56 years that had surgery after 12 weeks and before 20 weeks from diagnosis (GP) (%)	18.9	18.8	22.0	15.1	20.3	17.4	19.0	17.2	14.3
Patients <age 56 years that had surgery after 20 weeks from diagnosis (GP) (%)	49.0	51.7	50.8	58.7	54.8	57.7	60.4	61.6	68.5
Waiting time for surgery (days)	31	29	32	34	36	39	38	43	44

[a] number of surgical treated patients that could be linked between the NIVEL and NZA database [b] General Practitioner

For peer review only

STROBE Statement—Checklist of items that should be included in reports of ***cross-sectional studies***

	Item No	Recommendation
**Title and abstract**	1	(*a*) Indicate the study's design with a commonly used term in the title or the abstract (p1, l1)
		(*b*) Provide in the abstract an informative and balanced summary of what was done and what was found (p2)
**Introduction**		
Background/rationale	2	Explain the scientific background and rationale for the investigation being reported (p3)
Objectives	3	State specific objectives, including any prespecified hypotheses (p3, p4; l89-94)
**Methods**		
Study design	4	Present key elements of study design early in the paper (p4; l96)
Setting	5	Describe the setting, locations, and relevant dates, including periods of recruitment, exposure, follow-up, and data collection (p4; section data sources)
Participants	6	(*a*) Give the eligibility criteria, and the sources and methods of selection of participants (p4; section population)
Variables	7	Clearly define all outcomes, exposures, predictors, potential confounders, and effect modifiers. Give diagnostic criteria, if applicable (p5; section analyses)
Data sources/ measurement	8*	For each variable of interest, give sources of data and details of methods of assessment (measurement). Describe comparability of assessment methods if there is more than one group (p5, p6; section data collection, analyses, costa analysis and analytic approach)
Bias	9	Describe any efforts to address potential sources of bias (p5, p6; section analyses, cost analysis and analytic approach)
Study size	10	Explain how the study size was arrived at (n.a. (population-based))
Quantitative variables	11	Explain how quantitative variables were handled in the analyses. If applicable, describe which groupings were chosen and why (p6; section analytic approach)
Statistical methods	12	(*a*) Describe all statistical methods, including those used to control for confounding (p6; analytic approach)
		(*b*) Describe any methods used to examine subgroups and interactions (n.a.)
		(*c*) Explain how missing data were addressed (we had no missing data due to the administrative study design)
		(*d*) If applicable, describe analytical methods taking account of sampling strategy (n.a.)
		(*e*) Describe any sensitivity analyses (n.a.)
**Results**		
Participants	13*	(a) Report numbers of individuals at each stage of study—eg numbers potentially eligible, examined for eligibility, confirmed eligible, included in the study, completing follow-up, and analysed (p6; section study population)
		(b) Give reasons for non-participation at each stage (n.a.)
		(c) Consider use of a flow diagram (n.a.)
Descriptive data	14*	(a) Give characteristics of study participants (eg demographic, clinical, social) and information on exposures and potential confounders (p6; section study population)
		(b) Indicate number of participants with missing data for each variable of interest (n.a.)
Outcome data	15*	Report numbers of outcome events or summary measures (p7; l179 – p8 l211)

For peer review only - http://bmjopen.bmj.com/site/about/guidelines.xhtml

Main results	16	(*a*) Give unadjusted estimates and, if applicable, confounder-adjusted estimates and their precision (eg, 95% confidence interval). Make clear which confounders were adjusted for and why they were included (n.a. due to the study design)
		(*b*) Report category boundaries when continuous variables were categorized (n.a.)
		(*c*) If relevant, consider translating estimates of relative risk into absolute risk for a meaningful time period (not relevant)
Other analyses	17	Report other analyses done—eg analyses of subgroups and interactions, and sensitivity analyses (p8; section cost analysis)
**Discussion**		
Key results	18	Summarise key results with reference to study objectives (p8; l218-l224)
Limitations	19	Discuss limitations of the study, taking into account sources of potential bias or imprecision. Discuss both direction and magnitude of any potential bias (p10; l283-l294)
Interpretation	20	Give a cautious overall interpretation of results considering objectives, limitations, multiplicity of analyses, results from similar studies, and other relevant evidence (p11; section conclusion)
Generalisability	21	Discuss the generalisability (external validity) of the study results (not important due to the population-based design (all inhabitants are included)
**Other information**		
Funding	22	Give the source of funding and the role of the funders for the present study and, if applicable, for the original study on which the present article is based (p1)

*Give information separately for exposed and unexposed groups.

Note: An Explanation and Elaboration article discusses each checklist item and gives methodological background and published examples of transparent reporting. The STROBE checklist is best used in conjunction with this article (freely available on the Web sites of PLoS Medicine at http://www.plosmedicine.org/, Annals of Internal Medicine at http://www.annals.org/, and Epidemiology at http://www.epidem.com/). Information on the STROBE Initiative is available at www.strobe-statement.org.

For peer review only
